# Competition of the Addition/Cycloaddition Schemes in the Reaction Between Fluorinated Nitrones and Arylacetylenes: Comprehensive Experimental and DFT Study

**DOI:** 10.3390/molecules30234578

**Published:** 2025-11-28

**Authors:** Szymon Jarzyński, Andrzej Krempiński, Anna Pietrzak, Radomir Jasiński, Emilia Obijalska

**Affiliations:** 1 Faculty of Chemistry, Department of Organic Chemistry, University of Lodz, Tamka 12, 91-403 Lodz, Poland; szymon.jarzynski@chemia.uni.lodz.pl; 2 Faculty of Chemistry, Department of Organic and Applied Chemistry, University of Lodz, 91-403 Lodz, Poland; andrzej.krempinski@edu.uni.lodz.pl; 3 Institute of General and Ecological Chemistry, Lodz University of Technology, Żeromskiego 116, 90-924 Lodz, Poland; anna.pietrzak.1@p.lodz.pl; 4 Cracow University of Technology, Department of Organic Chemistry and Technology, Warszawska 24, 31-155 Kraków, Poland

**Keywords:** fluorinated nitrones, acetylenes, enantioselective synthesis, cycloaddition, DFT calculation

## Abstract

The course of the reactions of acetylenes with fluorinated nitrones in the presence of Zn(OTf)_2_ and Et_2_Zn was investigated. The formation of hydroxylamines and/or 1,2-oxazolines as products was observed. The desired hydroxylamines were formed as main products if reactions were carried out with the usage of Et_2_Zn. In order to explain the obtained results, quantum mechanical calculations of the reaction paths leading to both products were carried out. Further research allowed us to develop the enantioselective variant of described reactions with the usage of enantiomerically pure AziPhenol ligand bearing chiral aziridine scaffold.

## 1. Introduction

The synthesis of organofluorine compounds is an intensively developed field of organic chemistry. Compounds containing fluorine atoms (in particular small fluoroalkyl substituents) in their structure exhibit interesting physicochemical and biological properties [1,2,3,4]. On the other hand, nitrones are an extremely important class of building blocks that have been used for the synthesis of various organic derivatives [5,6]. For example, propargyl *N*-hydroxylamines obtained by the addition of acetylenes to nitrones constitute an interesting class of compounds that can be easily transformed into valuable compounds e.g., 4-isoxazolines, isoxazoles, pyrimidines, acylaziridines, α,β-unsaturated compounds [7,8,9,10,11,12,13]. Fluorinated nitrones, which are now easily synthesized from commercially available reagents [14,15,16], are powerful building blocks that can be used to incorporate CF_3_ and CHF_2_ substituents into organic molecules. To date, nitrones have not been widely explored in organic synthesis. Examples of their use are cycloadditions of nitrones with alkenes, alkynes leading to the formation of oxazolines [17], oxazolidines [18] and β-lactams [15]. Other reactions described in the literature are the addition of Grignard reagents to obtain the corresponding hydroxylamines [19]. Additionally, since data on enantioselective protocols of additions of nucleophiles to fluorinated nitrones have not been reported. The only example of an enantioselective reaction using nitrones derived from fluorinated aldehydes is the Kinugasa reaction but reported enantioselectivities were very low [15]. Several examples described in the literature concern enantioselective additions of nucleophiles to fluorinated imines [20].

This article describes the results of research on the addition of various acetylenes to nitrones derived from trifluoro- and difluoroacetaldehydes. Contrary to such reactions with non-fluorinated substrates [21], additions to fluorinated nitrones have not been studied. In the extension of the research, it was also decided to develop an enantioselective variant of the reaction.

## 2. Results and Discussion

**Scheme 1:**
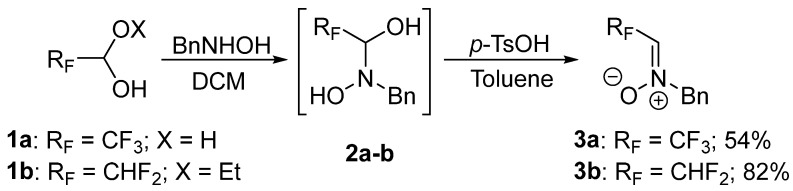
Synthesis of fluorinated nitrones.

The key starting materials, i.e., the nitrones **3a,b**, were obtained according to a previously published procedure involving the reaction of the appropriate hydroxylamine with trifluoroacetaldehyde hydrate or difluoroacetaldehyde ethyl hemiacetal (Scheme 1) [14,15,16].

### 2.1. Additions of Acetylenes to Fluorinated Nitrones

The goal of the studies was to investigate the scope and application of additions of diverse acetylenes to nitrones derived from fluorinated aldehydes and the elaboration of enantioselective protocol of this reaction. In the course of experimental work two procedures previously described in the literature for non-fluorinated substrates were applied [7,22]. Method A was based on the reaction of an appropriate nitrone with acetylene in the presence of zinc triflate and triethylamine [22]. The optimization of the amount of reagents used was performed for model substrates **3a** and **4a**. As described in the literature, it was noted that to obtain satisfactory reaction yields, a 2–3-fold excess of acetylene **4a** should be used.

**Scheme 2:**
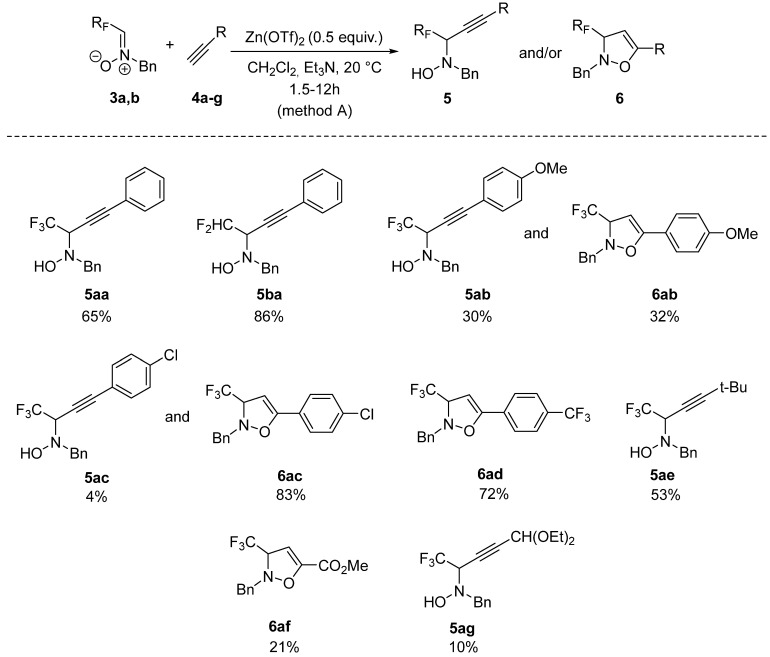
Additions of acetylenes to fluorinated nitrones in the presence of Zn(OTf)_2_.

The optimal amount of Zn(OTf)_2_ was 0.5 equivalents relative to the amount of used nitrone of type **3**. The application of larger amounts (eg. 1.2 equiv) of this catalyst did not significantly change the yield or the proportion of obtained products. Reactions of the *N*-alkyl-*C*-(fluoroalkyl) nitrones **3a,b** with the acetylenes **4a-g** were performed under optimized conditions (Scheme 2). The reaction time was monitored by TLC (the disappearance of the spot from the substrate was followed) and it varied (1.5–12 h) depending on the type of substituents in the used substrates. Longer reaction times were observed for substrates containing sterically hindered substituents or an electron-donating group on the aromatic ring. The change of the CF_3_ substituent to CHF_2_ in the molecule of the starting nitrone **3** did not change the reaction time, but the hydroxylamine **5ba** was isolated with a higher yield than product **5aa**. In the case of using phenylacetylene **4b** having an OMe substituent in the aryl ring, isoxazoline **6ab** and the expected hydroxylamine **5ab** were isolated as products in similar amounts. The incorporation of the electron-withdrawing Cl or CF_3_ group to the phenyl ring resulted in obtaining isoxazolines **6ac** and **6ad** as a major or sole product. A similar result was observed in the case of using methyl propiolate (**4g**) as a substrate. In the case of acetylene bearing a *tert*-butyl substituent, adduct **5ae** was isolated in comparable yield to that obtained in the reaction with **4a**. Using acetylenes **4h-k** with more complex substituents (CH(OMe)_2_, P(O)(OEt)_2_, piryd-2-yl, CH_2_OSiMe_3_), the expected products were obtained in low yields, or no product could be identified or isolated in pure form.

In the summary of this part of the research, it can be stated that the additions of acetylenes **4** to fluorinated nitrones **3** performed in the presence of zinc triflate did not lead to hydroxylamines **5** satisfactory results (in terms of hydroxylamine formation). Only in the case of using acetylenes with an alkyl or an unsubstituted phenyl ring attached to the carbon atom of a triple bond, the yields were satisfying. Therefore, carrying out the reaction under these conditions cannot be considered as a good general method for obtaining the expected fluorinated hydroxylamines of type **5**.

**Scheme 3:**
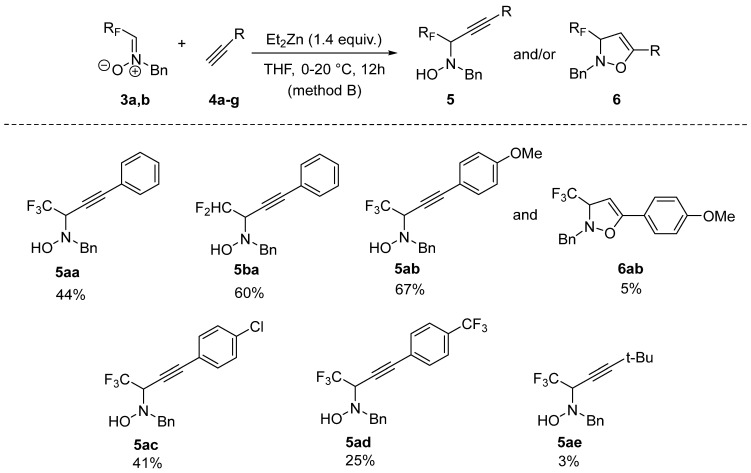
Addition of acetylenes to fluorinated nitrones in the presence of Et_2_Zn.

Due to unsatisfactory results of the reaction between *C*-(fluoroalkyl) nitrones of type **3** and acetylenes **4** obtained in the presence of a catalytic amount of Zn(OTf)_2_, it was decided to test other reaction conditions [7]. Additions were performed with a slight excess of substituted alkynylzinc generated using a stoichiometric amount of diethylzinc and the corresponding acetylenes **4a-k** (Scheme 3). It has been observed that the reactions conducted under these conditions give better results in terms of selectivity of obtained hydroxylamines **5** from aromatic acetylenes. It was noticed that in the case of the usage of acetylenes **4c,d** bearing electron-withdrawing substituents (Cl, CF_3_) in the aryl ring as substrates, the hydroxylamines **5** were isolated with lower yields than in reactions with acetylenes **4a,b** bearing an unsubstituted phenyl ring or electron-donating group attached to aryl ring. Unfortunately, the reaction carried out with the usage of the acetylene having a sterically hindered *t*-Bu substituent led to the formation of desired product **5ae** in trace amount. In the case of using of acetylenes containing substituents such as CH(OEt)_2_, CH_2_OSiMe_3_, CO_2_Me, P(O)(OEt)_2_, piryd-2-yl attached to the carbon atoms of a triple bond formation of unidentified decomposition products was observed.

**Scheme 4:**
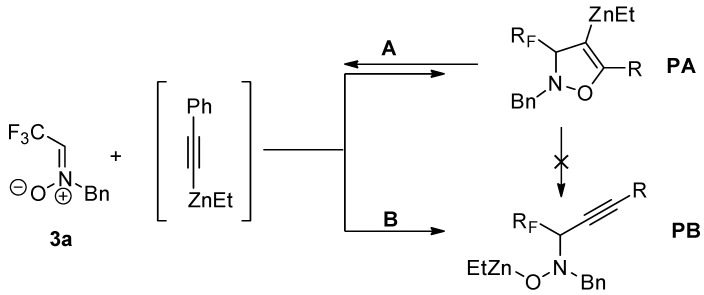
Competitive paths of the reaction between nitrone **3a** and (2-phenyletynyl)zinc considered in the framework of the DFT study.

**Figure 1:**
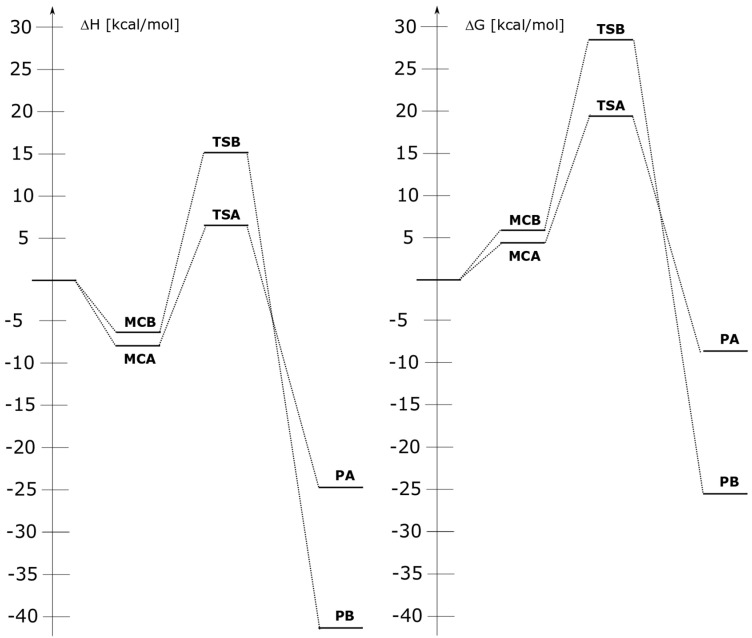
The enthalpy and Gibbs free energy profiles for the addition processes between nitrone **3a** and (2-phenyletynyl)zinc in the light of the wb97xd/6-311+G(d) (PCM) calculations.

**Figure 2:**
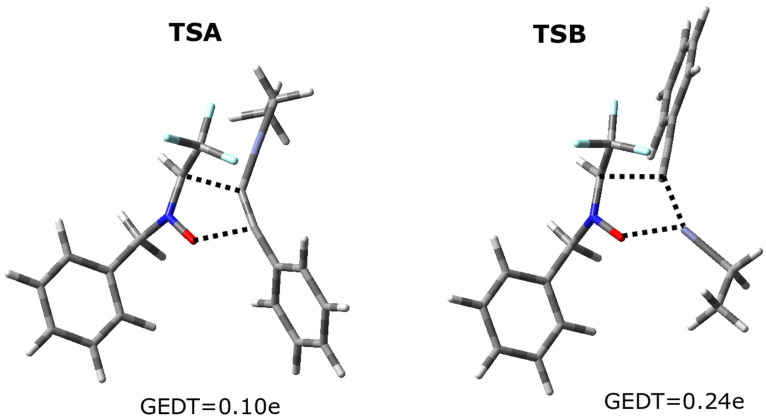
Views of TSs for the addition processes between nitrone **3a** and (2-phenyletynyl)zinc in the light of the wb97xd/6-311+G(d) (PCM) calculations.

The observed composition of postreaction mixtures can be explained on the basis of the comprehensive quantum chemical analysis of the reaction mechanism (Figure 1 and Figure 2; Scheme 4). For this purpose, results from the wb97xd/6-311+G(d) (PCM) computational study were used. Within these considerations, the addition process between nitrone **3a** and (2-phenyletynyl)zinc (Scheme 4) was used. This step is crucial because it is known from the literature that the energy of formation of (2-phenyletynyl)zinc from phenylacetylene **4a** and diethylzinc is negligible and does not limit the addition process [21,23].

Independently of the reaction path, the initial interactions between reagents lead to the formation of respective pre-reaction complex (**MCA** and **MCB** for paths **A** and **B,** respectively). This is connected with the decreasing of the enthalpy of reaction system about few kcal/mol. The entropic factor determines however, positive values of the Gibbs free energy for considered transformations. This excludes the existence of MCs as relatively stable intermediates. Within MCs, substructures of addents adopts the orientation determined the further course of transformation. It should be underlined, that any new bonds are not formed however at this stage. Next, the key interatomic distances exist beyond of the range typical for respective bonds within transition states [24]. Lastly, the electron density transfer (GEDT) between structures is not observed within both MCs. So, the localized structures can be considered as orientation, but not charge-transfer complexes [25]. The further transformation of MCs along the reaction coordinate leads to the area associated with the existence of the transition state (**TSA** and **TSB** for paths **A** and **B,** respectively). The clear increase in the energy of the reaction system is a consequence of this process. It is interesting that the favored from the kinetic point of view is the formation of the isoxazoline-type adduct via **TSA**. The structure of this TS is typical for the one-step [3 + 2] cycloaddition process with the participation of bent-type TACs [26,27,28] and exhibits a moderately polar nature (GEDT = 0.10e). For the contrast, the less kinetic favored **TSB** is evidently more polar (GEDT = 0.24e). Both TSs are connected directly with respective valleys of adducts. So, all new sigma-bonds must be formed at this stage. This was confirmed by the IRC analysis. Analysis of thermochemistry of products derived from IRC experiments show clearly that, from a thermodynamic point of view, the more favored is not [3 + 2] cycloaddition product (**PA**), but hydroxylamine derivative **PB**. So, in the light of our DFT computational study, the isoxazoline-type adduct should be treated as the primary reaction product, which is further converted to the more thermodynamically stable hydroxylamine-type product. It should be underlined-that the conversion of PA in PB is realized via dissociation to the individual nitrone–acetylene pair, and next, via the secondary addition according to the path **B**. All attempts for the localization of reaction channel leading directly from the P**A** in **PB** were not successful.

**Figure 3:**
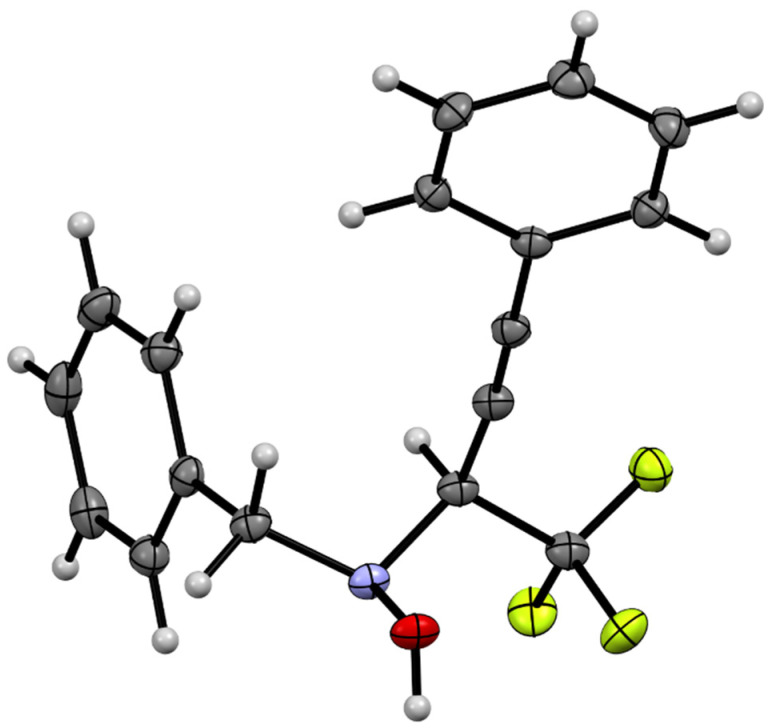
Single crystal X-ray analysis of **5aa**. Displacement ellipsoids are drawn at a 50% probability level.

The structures of all products were confirmed by spectroscopic methods. For example, a shift in the characteristic quartet derived from the hydrogen atom of the CH group from 6.87 ppm (**3a**) to 4.33 ppm (**5aa**) was observed in the ^1^H-NMR spectrum. Additionally, a singlet derived from the dynamic proton of the OH group (5.12 ppm) and two doublets (4.01 and 4.27 ppm) from the diastereotopic CH_2_ protons of the benzyl group in ^1^H-NMR spectrum of **5aa** appeared. Also, in the ^13^C-NMR spectrum, a shift in the quartet derived from the carbon atom of the C=N bond of the nitrone **3a** (122.6 ppm) to the region characteristic for the signals of the C-sp^3^ atom in the product molecule **5aa** (60.7 ppm) was observed. In the case of the ^1^H-NMR spectrum of an exemplary oxazoline **6ab**, the lack of a singlet derived from a proton of OH group was noticed. Instead, there was a doublet derived from olefinic proton of oxazoline ring (5.01 ppm). In addition, the signal of the proton of the CHCF_3_ group appeared in the form of a doublet of quartet at 4.43 ppm, which proves that there is a coupling with three fluorine atoms and with an olefinic proton. Also in the ^13^C-NMR spectrum recorded for **6ab** was a shift in the signals from carbon atoms of C≡C bond of hydroxylamine **5ab** occurring in the region characteristic for the signals of the C-sp atoms (75.0 and 89.5 ppm) to the region characteristic for the signals of the C-sp^2^ atoms (83.4 and 157.4 ppm) in the product molecule **6ab**. Eventually, the structure of the obtained hydroxylamines of type **5** was confirmed by an X-ray structure registered for one product **5aa** (Figure 3).

### 2.2. Enantioselective Protocol

**Scheme 5:**
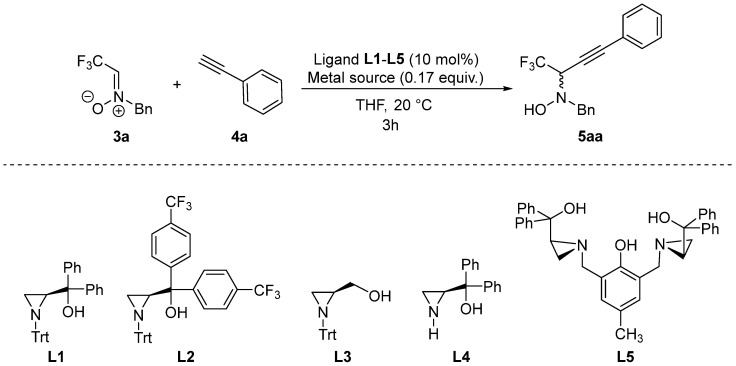
Additions of acetylene **4a** to nitrone **3a** in the presence of diverse chiral aziridine ligands.

Due to the better results of reactions carried out with the use of Et_2_Zn, the enantioselective protocol was also optimized with the use of this reagent. To this end, several ligands previously proven to be efficient in asymmetric catalysis were examined [29,30,31,32]. Our initial investigation began by testing several catalysts bearing the chiral aziridine ring reacting in situ with ZnEt_2_ to evaluate their catalytic ability. The optimization of the enantioselective addition of phenylacetylene **4a** to nitrone **3a** started with the screening of several chiral aziridine alcohols **L1**-**L5** which were synthesized as previously described (Scheme 5) [32,33,34]. Table 1 summarizes the comparison of the results obtained for various chiral ligands. The simple β-amino alcohol catalysts **L1**-**L4** exhibited poor enantioselectivity. Fortunately, the ligand screening compromised that the AziPhenol ligand **L5** provided the best results in terms of both the yield and enantioselectivity, and the desired product was isolated in 67% yield and 44% *ee*. When the metal source was changed from ZnEt_2_ to Zn(OTf)_2_, a pronounced decrease in both the yield and enantiomeric excess was observed (Table 1, entry 6). Consequently, the most efficient ligand **L5** was selected for further optimization involving solvent, temperature, catalyst loading, and additive effects.

**Scheme 6:**
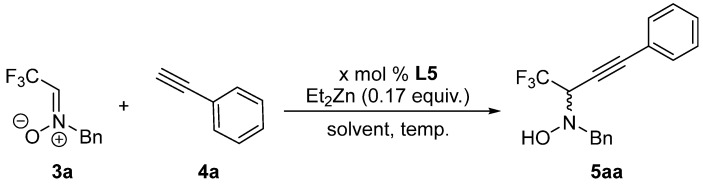
Enantioselective additions of phenylacetylene (**4a**) to nitrone **3a** in diverse solvents.

The initial studies towards the development of efficient conditions began with the evaluation of various solvents (Scheme 6). The screening of a variety of solvents including toluene, dichloromethane (DCM) and diethyl ether (Et_2_O) was performed (Table 2, entries 2–4). The reactions carried out in halogenated and aromatic solvents, such as dichloromethane and toluene, proceeded in good yields, but significantly lower enantioselectivity was observed. As shown in Table 2, the use of THF afforded superior results with respect to the yield and enantiomeric excess (entry 1) compared with the other solvents examined (entries 2–4). Subsequently, the catalyst loading amount and reaction temperature were also screened. Temperature was shown to have a significant effect on the yields and enantioselectivity (Table 2, entries 5–7). Decreasing the reaction temperature from 20 °C to −20 °C improved both the yield and stereoselectivity of the product **5aa** formation; however, it also resulted in a longer reaction time. Additionally, lowering the temperature to −78 °C caused a small decrease in yield and ee of product **5aa**. No significant changes in the stereoselectivities of the reaction were observed when a 20 mol % catalyst was used (Table 2, entry 8). Reducing the catalyst loading to 5 mol% caused a slight decrease in both the yield and enantiomeric excess of the product, and a longer reaction time was required. It revealed that 10 mol% of ligand is necessary for optimal yield and enantioselectivity. Further optimization aimed at improving catalytic activity revealed that 4 Å molecular sieves significantly influenced both the reactivity and stereoselectivity of the system. Such additives are known to play a crucial role in numerous zinc-catalyzed asymmetric transformations [35,36]. To our great delight, when 4 Å molecular sieves were added to the reaction, the conversion and enantioselectivity were enhanced; the desired product was obtained in 80% yield and 58% ee. Therefore, the optimized reaction conditions are summarized in entry 10 of Table 2.

**Scheme 7:**
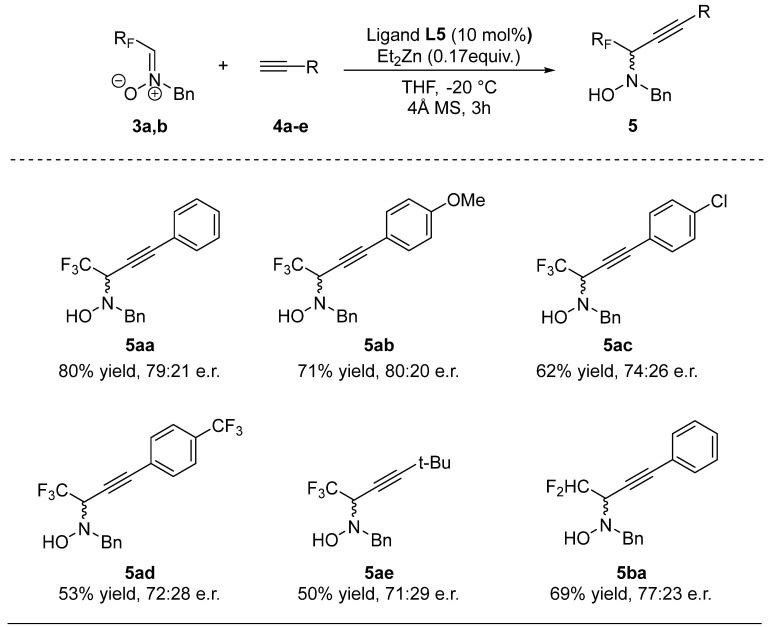
Enantioselective additions of acetylenes **4a-e** to nitrones **3a-b** under the optimal conditions (All reactions were carried out with **3** (0.1 mmol), **4** (0.15 mmol), ligand (0.01 mmol) and Et_2_Zn (0.17 mmol) in THF (2.0 mL) under nitrogen at −20 °C for 3 h; 20 mg of 4Å MS was added; Isolated yield after column chromatography; Determined by chiral HPLC).

With the optimized reaction conditions in hand, we next examined the substrate scope of the developed protocol (Scheme 7). Regardless of the electronic nature of the substituents on the phenyl ring, the reactions afforded good yields, although variations in enantioselectivity were observed. Substrate **4b**, which contained an electron-donating substituent (-OMe) in the *para* position of the phenyl ring, was well tolerated and led to the desired product in the highest yield and enantioselectivity. It was noticed that in the case of the usage of acetylenes bearing electron-withdrawing substituents (Cl, CF_3_) in the aryl ring as substrates, the products were isolated in lower yields and enantioselectivities. Surprisingly, in the presence of chiral ligands, the reaction product of acetylene **4e** with a sterically hindered *t*-Bu substituent was obtained in good yield but the decrease in yield and enantioselectivity was observed. However, changing of the CF_3_ substituent to CHF_2_ in the starting nitrone **3** resulted in a slight decrease in the yield, but the desired product was isolated with similar enantiomeric excess. In contrast, extending the reaction time to 12 h under the optimized conditions led to the formation of a complex reaction mixture, from which the cyclic products **6aa**-**6ad** were isolated in low yields and with poor enantioselectivity (<10%).

## 3. Materials and Methods

### 3.1. General Information

Solvents and chemicals were purchased and used without further purification. Tetrahydrofuran (THF) and toluene (PhMe) were distilled over sodium/benzophenone(violet-colored solution prior to use). Dichloromethane (DCM) was distilled over sodium hydride. Zinc triflate (Zn(OTf)_2_), diethyl zinc (Et_2_Zn) (1M solution in hexane), triethylamine (Et_3_N) and diverse acetylenes were purchased from Sigma-Aldrich (Merck, Poznań, Poland). Obtained products were purified by standard column chromatography on silica gel (230–400 mesh Merck, Poznań, Poland) or FLASH column chromatography using Grace Reveleris X2 apparatus with UV–Vis and ELSD detection (commercially available 12 g SiO_2_ columns, pressure 20–25 psi, solvent flow rate 25–28 mL/min). Unless stated otherwise, yields refer to analytically pure samples. NMR spectra were recorded with Bruker Avance III 600 MHz (^1^H NMR [600 MHz]; ^13^C NMR [151 MHz]; ^19^F NMR [565 MHz]) instrument. Chemical shifts are reported in ppm relative to solvent residual peaks (^1^H NMR: δ = 7.26 ppm [CHCl_3_]; ^13^C NMR: δ = 77.0 ppm [CDCl_3_]). For detailed peak assignments 2D (HMQC) spectra were measured. IR Spectra were measured with an Agilent Cary 630 FTIR spectrometer (in neat). MS Spectra were recorded on Varian 500 MS LS IonTrap spectrometer. The HPLC analysis were carried out using an Agilent 1260 Infinity system (Agilent, Waldbronn, Germany)at 25 °C using chiral columns Chiralcel AD and Chiralcel AD-H. Single-crystal XRD measurements were performed with a Rigaku XtaLAB Synergy, Pilatus 300K diffractometer. Melting points were determined in capillaries with a Stuart SMP30 apparatus (Stone, United Kingdom).

### 3.2. Synthesis of Nitrones Derived from Trifluoroacetaldehyde and Difluoroacetaldehyde

*N*-Benzyl *C*-(trifluoromethyl)nitrone (**3a**) and *N*-Benzyl *C*-(difluoromethyl)nitrone (**3b**) were prepared following the published protocol [14,15,16]. Chiral ligands (**L1-L5**) were also synthetized according to previously described methods [32,33,34]. More experimental data, e.g., NMR spectra of the products **5**,**6**, chromatograms and RTG structure details of nitrone **3a** and hydroxylamine **5aa** can be found in Supplementary Material.

### 3.3. Reactions of Nitrones ***3*** Derived from Fluorinated Aldehydes with Acetylenes ***4***

*(a)*  *Reactions leading to racemic products*

*Method A—A general procedure*: Appropriate acetylene **4a-g** (3 mmol) and Et_3_N (152 mg, 1.5 mmol) were added to a suspension of Zn(OTf)_2_ (182 mg, 0.5 mmol) in anhydrous DCM (5 mL) placed in a dried round-bottom flask. The reaction was carried out under an inert gas atmosphere (argon). This mixture was magnetically stirred for 20 min at room temperature. Next, a solution of a corresponding nitrone **3a,b** (1 mmol) in DCM (1 mL). was added dropwise. The progress of the reaction was controlled by TLC (SiO_2_; petroleum ether: ethyl acetate 3:2) and reaction completion time varied (1.5 h–12 h). Then a saturated NH_4_Cl solution was added to the reaction mixture. The organic layer was separated, and water layer was extracted with DCM (3 × 15 mL). Combined organic layers were dried over anhydrous Na_2_SO_4_ and filtrated. Next, the solvent was evaporated under reduced pressure. Products were purified by automated flash chromatography (SiO_2_ 12 g cartridges; petroleum ether with increasing amount of DCM (0–60%) as an eluent).

*Method B—A general procedure*: Appropriate acetylene **4a-g** (1.4 mmol) was added to a solution of diethylzinc (1.4 mmol) in anhydrous THF placed in a dried round-bottom flask. The reaction was carried out under an inert gas atmosphere (argon). This mixture was magnetically stirred for 20 min at room temperature. Then the flask was placed in an ice bath (~0 °C) and next a solution of a corresponding nitrone **3a,b** (1 mmol) in THF (1 mL) was added dropwise. The progress of the reaction was controlled by TLC (SiO_2_; petroleum ether: ethyl acetate 3:2) and the time of completion of the reaction was about 12 h. Then a saturated NH_4_Cl solution was added to the reaction mixture. The organic layer was separated, and water layer was extracted with DCM (3 × 15 mL). Combined organic layers were dried over anhydrous Na_2_SO_4_ and filtrated. Next, the solvent was evaporated under reduced pressure. Products were purified by automated flash chromatography (SiO_2_ 12 g cartridges; petroleum ether with increasing amount of DCM (0–60%) as an eluent). Analytically pure products were, in most cases, obtained by crystallization from an appropriate solvent.

*N-Benzyl-N-(1,1,1-trifluoro-4-phenylbut-3-yn-2-yl)hydroxylamine* (**5aa**). Yield: 198 mg (65%) (for method A), 134 mg (44%) (for method B); colorless crystals, m.p. 102–104 °C (DCM/petroleum ether). ^1^H NMR (600 MHz, CDCl_3_): δ 4.01, 4.27 (2d, 2H, ^2^*J*_H,H_ = 12.84 Hz, C**H**_2_Ph), 4.33 (q, 1H, ^3^*J*_H,F_ = 6.84 Hz, C**H**), 5.12 (s, 1H, O**H**), 7.32–7.43 (m, 8 arom. **H**), 7.56–7.59 (m, 2 arom. **H**) ppm. ^13^C NMR (150 MHz, CDCl_3_): δ 60.7 (q, ^2^*J*_C,F_ = 31.5 Hz, **C**H), 62.2 (**C**H_2_Ph), 76.5 (q, ^3^*J*_C,F_ = 3,0 Hz, **C**≡CPh), 89.5 (C≡**C**Ph), 121.6, 135.9 (2 arom. **C**), 123.1 (q, ^1^*J*_C,F_ = 279.0 Hz, **C**F_3_), 128.0, 128.4, 128.6, 129.2, 129.4, 132.2 (10 arom. **C**H) ppm. ^19^F NMR (565 MHz, CDCl_3_): δ −71.47 (d, 3F, ^3^*J*_F,H_ = 6.84 Hz, C**F**_3_) ppm. IR: *v* 3275*m* (O-H), 2879*w*, 1491*w*, 1463*w*, 1359*m*, 1289*m*, 1187*s*, 1139*vs*, 1075*s*, 1030*m*, 818*s*, 751*vs*, 689*vs* cm^−1^. ESI-MS (*m*/*z*): 306 (60, [M+H]^+^), 328 (100, [M+Na] ^+^). Elemental analysis for C_17_H_14_F_3_NO (305.3) calculated: C 66.88, H 4.62, N 4.59; found: C 66.90, H 4.58, N 4.83.

*N-Benzyl-N-(1,1-difluoro-4-phenylbut-3-yn-2-yl)hydroxylamine* (**5ba**). Yield: 247 mg (86%) (for method A), 172 mg (60%) (method B); colorless crystals m.p. 85–87 °C (DCM/petroleum ether). ^1^H NMR (600 MHz, CDCl_3_): δ 3.99, 4.23 (2d, 2H, ^2^*J*_H,H_ = 12.84 Hz, C**H**_2_Ph), 4.04–4.09 (m, 1H, C**H**), 5.06 (s, 1H, O**H**), 6.01 (dt, 1H, ^3^*J*_H,H_ = 5.10 Hz, ^2^*J*_H,F_ = 56.05 Hz, C**H**F_2_), 7.31–7.42 (m, 8 arom. **H**), 7.55–7.57 (m, 2 arom. **H**) ppm. ^13^C NMR (150 MHz, CDCl_3_): δ 61.6 (t, ^2^*J*_C,F_ = 25.5 Hz, **C**H), 62.3 (**C**H_2_Ph), 78.6 (dd, ^3^*J*_C,F_ = 3.0 Hz, **C**≡CPh), 89.4 (C≡**C**Ph), 113.7 (dd, ^1^*J*_C,F(1)_ = 243.0 Hz, ^1^*J*_C,F(2)_ = 244.5 Hz, **C**HF_2_), 121.9, 135.9 (2 arom. **C**) 128.0, 128.4, 128.6, 129.0, 129.6, 132.1 (10 arom. **C**H) ppm. ^19^F NMR (565 MHz, CDCl_3_): δ −125.22 (ddd, 1F, ^3^*J*_F(1),H_ = 10.79 Hz, ^2^*J*_F(1),H_ = 56.05 Hz, ^2^*J*_F,F_ = 284.15 Hz, CH**F**_2_), −121.55 (ddd, 1F, ^3^*J*_F(2),H_ = 8.02 Hz, ^2^*J*_F(2),H_ = 56.50 Hz, ^2^*J*_F,F_ = 284.15 Hz, CH**F**_2_) ppm. IR: *v* 3237*m* (O-H), 2876*w*, 1491*w*, 1444*m*, 1384*m*, 1109*s*, 1087*s*, 1060*s*, 689*vs*, 540*vs* cm^−1^. ESI-MS (*m*/*z*): 288 (45, [M+H]^+^), 310 (100, [M+Na]^+^). Elemental analysis for C_17_H_15_F_2_NO (287.3) calculated: C 71.07, H 5.26, N 4.88; found: C 71.26, H 5.02, N 5.17.

*N-Benzyl-N-[1,1,1-trifluoro-4-(4′-methoxyphenyl)but-3-yn-2-yl]hydroxylamine* (**5ab**). Yield: 101 mg (30%) (method A), 225 mg (67%) (method B); colorless crystals, m.p. 102–104 °C (Et_2_O/petroleum ether). ^1^H NMR (600 MHz, CDCl_3_): δ 3.84 (s, 3H, OC**H**_3_), 3.99, 4.24 (2d, 2H, ^2^*J*_H,H_ = 12.78 Hz, C**H**_2_Ph), 4.31 (q, 1H, ^3^*J*_H,F_ = 6.84 Hz, C**H**), 5.22 (br.s, 1H, O**H**), 6.87–6.89 (m, 2 arom. **H**), 7.31–7.34 (m, 1 arom. **H**), 7.36–7.38 (m, 2 arom. **H**), 7.41–7.42 (m, 2 arom. **H**), 7.50–7.51 (m, 2 arom. **H**) ppm. ^13^C NMR (150 MHz, CDCl_3_): δ 55.3 (O**C**H_3_), 60.7 (q, ^2^*J*_C,F_ = 31.5 Hz, **C**H), 62.2 (**C**H_2_Ph), 75.0 (**C**≡CPh), 89.5 (C≡**C**Ph), 113.5, 135.8, 160.2 (3 arom. **C**), 114.0, 128.0, 128.7, 129.4, 133.7 (9 arom. **C**H) ppm. ^19^F NMR (565 MHz, CDCl_3_): δ −71.58 (d, 3F, ^3^*J*_F,H_ = 6.84 Hz, C**F**_3_) ppm. IR: *v* 3269*m* (O-H), 3026*w*, 2933*w*, 2885*w*, 2236*w*, 1610*m*, 1513*s*, 1454*m*, 1357*m*, 1271s, 1174*s*, 1133*vs*, 1000*s*, 972*w*, 827*vs*, 760*s* cm^−1^. ESI-MS (*m*/*z*): 336 (100, [M+H]^+^). Elemental analysis for C_18_H_16_F_3_NO (335.1) calculated: C 64.47, H 4.81, N 4.18; found: C 64.47, H 4.92, N 4.47.

*2-Benzyl-5-(4′-methoxyphenyl)-3-(trifluoromethyl)-2,3-dihydroizoxazole* (**6ab**). Yield: 107 mg (32%) (method A); 17 mg (5%) (method B); colorless crystals; m.p. 84–85 °C (Et_2_O/petroleum ether). ^1^H NMR (600 MHz, CDCl_3_): δ 3.83 (s, 3H, CH_3_), 4.02, 4.38 (2d, 2H, ^2^*J*_H,H_ = 12.80 Hz, C**H**_2_Ph), 4.43 (dq, 1H, ^3^*J*_H,H_ = 2.90 Hz, ^3^*J*_H,F_ = 6.50 Hz, C**H**CF_3_), 5.01 (d, 1H, ^3^*J*_H,H_ = 2.90 Hz, C=C**H**), 6.94–6.85 (m, 2 arom. **H**), 7.40–7.29 (m, 3 arom. **H**), 7.45–7.40 (m, 2 arom. **H**), 7.53–7.45 (m, 2 arom. **H**) ppm. ^13^C NMR (150 MHz, CDCl_3_): δ55.5 (**C**H_3_), 63.7 (**C**H_2_Ph), 70.8 (q, ^2^*J*_C,F_ = 32.3 Hz, **C**HCF_3_), 83.4 (q, C=**C**H), 114.1, 127.8, 128.2, 128.7, 129.8 (9 arom. **C**H), 124.2 (q, ^1^*J*_C,F_ = 282.0 Hz, **C**F_3_), 120.1, 134.9, 161.0 (3 arom. **C**), 157.4 (**C**=CH) ppm. ^19^F NMR (565 MHz, CDCl_3_): δ −77.78 (d, 3F, ^3^*J*_F,H_ = 6.50 Hz, C**F**_3_) ppm. **IR**: *v* 3034*w*, 2974*w*, 2940*w*, 2847*w*, 1662*m*, 1606*m*, 1513*m*, 1457*w*, 1428*w*, 1375*w*, 1256*s*, 1167*s*, 1126*vs*, 1047*m*, 1021*m*, 880*m*, 834*m* cm^−1^. ESI-MS (*m*/*z*): 336 (100, [M+H]^+^). Elemental analysis for C_18_H_16_F_3_NO (335.1) calculated: C 64.47, H 4.81, N 4.18; found: C 63.39, H 6.39, N 14.97.

*N-Benzyl-N-[1,1,1-trifluoro-4-(4′-chlorophenyl)but-3-yn-2-yl]hydroxylamine* (**5ac**). Yield: 14 mg (4%) (method A), 139 mg (41%) (method B); colorless crystals, m.p. 78–80 °C (Et_2_O/petroleum ether) ^1^H NMR (600 MHz, CDCl_3_): δ 3.99, 4.24 (2d, 2H, ^2^*J*_H,H_ = 12.78 Hz, C**H**_2_Ph), 4.31 (q, 1H, ^3^*J*_H,F_ = 6.84 Hz, C**H**), 5.21 (s, 1H, O**H**), 7.32–7.41 (m, 7 arom. **H**), 7.48–7.50 (m, 2 arom. **H**) ppm. ^13^C NMR (150 MHz, CDCl_3_): δ 60.6 (q, ^2^*J*_C,F_ = 31.6 Hz, **C**H), 62.2 (**C**H_2_Ph), 77.6 (**C**≡CPh), 88.2 (C≡**C**Ph), 120.0, 135.3, 135.7 (3 arom. **C**), 123.0 (q, ^1^*J*_C,F_ = 279.2 Hz, **C**F_3_), 128.1, 128.6, 128.7, 129.4, 133.4 (9 arom. **C**H) ppm. ^19^F NMR (565 MHz, CDCl_3_): δ −71.41 (d, 3F, ^3^*J*_F,H_ = 6.84 Hz, C**F**_3_) ppm. IR: *v* 3273*m* (O-H), 2877*w*, 2225*w*, 1490*s*, 1457*m*, 1394*m*, 1349*s*, 1263*s*, 1185*s*, 1133*vs*, 1085*vs*, 1006*s*, 972*m*, 920*m*, 979*m*, 820*s*, 753*s*, 700*s* cm^−1^. ESI-MS (*m*/*z*): 340 (100, [M_Cl-35_+H]^+^), 342 (36, [M _Cl-37_+H]^+^). Elemental analysis for C_17_H_13_ClF_3_NO (339.7) calculated: C 60.10, H 3.86, N 4.12; found: C 60.00, H 3.78, N 4.42.

*2-Benzyl-5-(4′-chlorophenyl)-3-(trifluoromethyl)-2,3-dihydroizoxazole* (**6ac**). Yield: 302 mg (89%) (method A); colorless crystals; m.p. 116–117 °C (Et_2_O/petroleum ether). ^1^H NMR (600 MHz, CDCl_3_): δ 4.04, 4.37 (2d, 2H, ^2^*J*_H,H_ = 12.90 Hz, C**H**_2_Ph), 4.47 (dq, 1H, ^3^*J*_H,H_ = 2.90 Hz, ^3^*J*_H,F_ = 6.50 Hz, C**H**CF_3_), 5.14 (d, 1H, ^3^*J*_H,H_ = 2.90 Hz, C=C**H**), 7.39–7.32 (m, 5 arom. **H**), 7.42–7.40 (m, 2 arom. **H**), 7.50–7.46 (m, 2 arom. **H**) ppm. ^13^C NMR (150 MHz, CDCl_3_): δ 63.8 (**C**H_2_Ph), 70.8 (q, ^2^*J*_C,F_ = 32.5 Hz, **C**HCF_3_), 86.0 (q, ^3^*J*_C,F_ = 1.5 Hz, C=**C**H), 124.0 (q, ^1^*J*_C,F_ = 280.2 Hz, **C**F_3_), 126.0, 134.6, 136.1 (3 arom. **C**), 127.5, 128.3, 128.7, 129.0, 129.8 (10 arom. **C**H), 156.6 (**C**=CH) ppm. ^19^F NMR (565 MHz, CDCl_3_): δ −71.32 (d, 3F, ^3^*J*_F,H_ = 6.50 Hz, C**F**_3_) ppm. IR: *v* 3127*w*, 3090*w*, 3034*w*, 2959*w*, 2896*w*, 2840*w*, 1651*w*, 1490*m*, 1453*w*, 1375*m*, 1341*m*, 1275*s*, 1222*m*, 1163*s*, 1129*vs*, 1085*m*, 1039*m*, 1010*m*, 879*m*, 834*m*, 798*m* cm^−1^. ESI-MS (*m*/*z*): 340 (100, [M_Cl-35_+H]^+^), 342 (28, [M_Cl-37_+H]^+^). Elemental analysis for C_17_H_13_ClF_3_NO (339.7) calculated: C 60.10, H 3.86, N 4.12; found: C 60.06, H 3.87, N 4.28.

*N-Benzyl-N-[1,1,1-trifluoro-4-(4′-trifluoromethylphenyl)but-3-yn-2-yl]hydroxylamine* (**5ad**). Yield: 93 mg (25%) (method B); colorless crystals; m.p. 101–103 °C (Et_2_O/petroleum ether). ^1^H NMR (600 MHz, CDCl_3_): δ 4.00, 4.26 (2d, 2H, ^2^*J*_H,H_ = 12.84 Hz, C**H**_2_Ph), 4.33 (q, 1H, ^3^*J*_H,F_ = 6.84 Hz, C**H**), 5.16 (s, 1H, O**H**), 7.32–7.42 (m, 5 arom. **H**), 7.62–7.63 (m, 2 arom. **H**), 7.66–7.67 (m, 2 arom. **H**) ppm. ^13^C NMR (150 MHz, CDCl_3_): δ 60.5 (q, ^2^*J*_C,F_ = 31.7 Hz, **C**H), 62.3 (**C**H_2_Ph), 79.2 (q, ^3^*J*_C,F_ = 1.5 Hz, **C**≡CPh), 87.8 (C≡**C**Ph), 123.0 (q, ^1^*J*_C,F_ = 279.3 Hz, **C**F_3_), 123.7 (q, ^1^*J*_C,F_ = 270.5 Hz, **C**F_3_), 125.3 (q, ^3^*J*_C,F_ = 7.5 Hz, 2 arom. **C**H), 128.1, 128.7, 129.4, 132.5 (9 arom. **C**H), 130.9 (q ^2^*J*_C,F_ = 7.5 Hz, 1 arom. **C**), 135.6 (1 arom. **C**) ppm. ^19^F NMR (565 MHz, CDCl_3_): δ −62.90 (s, 3F, ^3^*J*_F,H_ = 6.84 Hz, C**F**_3_), −77.53 (d, 3F, ^3^*J*_F,H_ = 6.84 Hz, CHC**F**_3_) ppm. IR: *v* 3422*m* (O-H), 3272*w*, 1618*m*, 1457*m*, 1405*m*, 1327*s*, 1275*s*, 1174*s*, 1126*vs*, 1003*vs*, 1050*s*, 1014*s*, 954*w*, 879*w*, 835*s*, 745*s* cm^−1^. ESI-MS (*m*/*z*): 374 (100, [M+H]^+^). Elemental analysis for C_18_H_13_F_6_NO (373.1) calculated: C 57.92, H 3.51, N 3.71; found: C 57.83, H 3.54, N 3.98.

*2-Benzyl-5-(4′-trifluoromethylphenyl)-3-(trifluoromethyl)-2,3-dihydroizoxazole* (**6ad**). Yield: 269 mg (72%) (method A); colorless crystals; m.p. 129–131 °C (Et_2_O/petroleum ether). ^1^H NMR (600 MHz, CDCl_3_): δ 4.06 (d, 1H, ^2^*J*_H,H_ = 12.60 Hz, C**H**_2_Ph), 4.39 (d, 1H, ^2^*J*_H,H_ = 12.60 Hz, C**H**_2_Ph), 4.51 (dq, 1H, ^3^*J*_H,H_ = 3.00 Hz, ^3^*J*_H,F_ = 6.06 Hz, C**H**CF_3_), 5.28 (d, 1H, ^3^*J*_H,H_ =3.00 Hz, C=C**H**), 7.33–7.39 (m, 3 arom. **H**), 7.42–7.43 (m, 2 arom. **H**), 7.63–7.67 (m, 4 arom. **H**) ppm. ^13^C NMR (150 MHz, CDCl_3_): δ 63.7 (**C**H_2_Ph), 70.6 (q, ^2^*J*_C,F_ = 32.3 Hz, **C**HCF_3_), 87.5 (C=**C**H), 123.7 (q, ^1^*J*_C,F_ = 270.5 Hz, **C**F_3_), 123.8 (q, ^1^*J*_C,F_ = 278.4 Hz, **C**F_3_), 125.5 (q, ^3^*J*_C,F_ = 3.8 Hz, 2 arom. **C**H) 126.4, 128.3, 128.6, 129.6 (7 arom. **C**H), 130.6, 134.3 (2 arom. **C**), 131.7 (q, ^2^*J*_C,F_ = 27.6 Hz, 1 arom. **C**) 156.1 (**C**=CH) ppm. ^19^F NMR (565 MHz, CDCl_3_): δ −62.90 (s, 3F, C**F**_3_), −77.53 (d, 3F, ^3^*J*_F,H_ = 6.06 Hz, CHC**F**_3_) ppm. IR: *v* 3127*w*, 3041*w*, 2903*w*, 1651*w*, 1414*w*, 1367*w*, 1330*m*, 1274*m*, 1159*s*, 1107*vs*, 1060*s*, 1017*m*, 850*s* cm^−1^. ESI-MS (*m*/*z*): 374 (35, [M+H]^+^), 396 (100, [M+Na]^+^). Elemental analysis for C_18_H_13_F_6_NO (373.1) calculated: C 57.92, H 3.51, N 3.71; found: C 57.73, H 3.69, N 4.01.

*N-Benzyl-N-(1,1,1-trifluoro-5,5-dimethylheks-3-yn-2-yl)hydroxyloamine* (**5ae**). Yield: 151 mg (53%) (method A), 9 mg (3%) (method B); colorless crystals; m.p. 75–77 °C (DCM/petroleum ether). ^1^H NMR (600 MHz, CDCl_3_): δ 1.32 (s, 9H, 3C**H**_3_), 3.87, 4.15 (2d, 2H, ^2^*J*_H,H_ = 12.85 Hz, C**H**_2_Ph), 4.07 (q, 1H, ^3^*J*_H,F_ = 6.95 Hz, C**H**), 4.94 (s, 1H, O**H**), 7.30–7.33 (m, 2 arom. **H**). 7.34–7.38 (m, 3 arom. **H**) ppm. ^13^C NMR (150 MHz, CDCl_3_): δ 27.7 (**C**(CH_3_)_3_), 30.8 (3**C**H_3_), 60.2 (q, ^2^*J*_C,F_ = 31.5 Hz, **C**H), 62.0 (**C**H_2_Ph), 65.6 (q, ^3^*J*_C,F_ = 3.0 Hz, **C**≡Ct-Bu), 99.2 (C≡**C**t-Bu), 123.2 (q, ^1^*J*_C,F_ = 279.0 Hz, **C**F_3_), 127.9, 128.6, 129.4 (5 arom. **C**H), 136.0 (1 arom. **C**H) ppm. ^19^F NMR (565 MHz, CDCl_3_): δ −72.02 (d, 3F, ^3^*J*_F,H_ = 6.95 Hz, C**F**_3_) ppm. IR: *v* 3269*m* (O-H), 2970*m*, 2922*m*, 2873*m*, 2240*w*, 1457*m*, 1360*s*, 1278*s*, 1203*w*, 1166*s*, 1130*vs*, 1092*m*, 1062*m*, 998*m*, 976*w*, 924*w*, 864*m*, 820*s*, 764*s*, 701*vs* cm^−1^. ESI-MS (*m*/*z*): 286 (100, [M+H]^+^), 308 (55, [M+Na]^+^). Elemental analysis for C_15_H_18_F_3_NO (285.3) calculated: C 63.15, H 6.36, N 4.91; found: C 63.39, H 6.39, N 5.14.

*Methyl 2-Benzyl-3-(trifluoromethyl)-4-isoxazoline-5-carboxylate* (**6af**). Yield: 60 mg (21%) (method A); colorless crystals; m.p. 81–84 °C (DCM/petroleum ether). ^1^H NMR (600 MHz, CDCl_3_): δ 3.85 (s, 3H, CO_2_C**H**_3_) 4.0, 4.38 (2d, 2H, ^2^*J*_H,H_ = 12.96 Hz, C**H**_2_Ph), 4.46 (dq, 1H, ^4^J_H,H_ = 3.06 Hz, ^3^*J*_H,F_ = 6.40 Hz, C**H**), 5.65 (d, 1H, ^4^J_H,H_ = 3.06 Hz, C=C**H**), 7.32–7.39 (m, 5 arom. **H**) ppm. ^13^C NMR (150 MHz, CDCl_3_): δ 52.7 (CO_2_**C**H_3_), 63.5 (**C**H_2_Ph), 70.0 (q, ^2^*J*_C,F_ = 31.5 Hz, **C**H), 99.6 (q, ^3^*J*_C,F_ = 1.5 Hz, **C**H=CCO_2_CH_3_), 123.2 (q, ^1^*J*_C,F_ = 279.0 Hz, **C**F_3_), 128.4, 128.7, 129.7 (5 arom. **C**H), 133.5 (1 arom. **C**), 149.5 (CH=**C**CO_2_CH_3_), 158.2 (**C**=O) ppm. ^19^F NMR (565 MHz, CDCl_3_): δ −77.02 (d, 3F, ^3^*J*_F,H_ = 6.40 Hz, C**F**_3_) ppm. IR: *v* 3145*w*, 3090*w*, 3012*w*, 2959*w*, 2900*w*, 1733*vs* (C=O), 1647*m*, 1446*m*, 1334*m*, 1278*m*, 1226*s*, 1177*s*, 1121*vs*, 1067*m*, 1010*m*, 969*m*, 879*m*, 801*m*, 723*vs* cm^−1^. ESI-MS (*m*/*z*): 288 (35, [M+H]^+^), 310 (100, [M+Na]^+^). Elemental analysis for C_13_H_12_F_3_NO_3_ (287.1) calculated: C 54.36, H 4.21, N 4.88; found: C 54.27, H 4.39, N 5.00.

*N-Benzyl-N-(5,5-diethoxy-1,1,1-trifluoropent-3-yn-2-yl)hydroxylamine* (**5ag**). Yield: 33 mg (10%) (method A); colorless oil. ^1^H NMR (600 MHz, CDCl_3_): δ 1.25–1.28 (m, 6H, 2OCH_2_C**H**_3_), 3.63–3.69 (m, 2H, OC**H**_2_CH_3_), 3.76–3.83 (m, 2H, C**H**_2_CH_3_), 3.91, 4.19 (2d, 2H, ^2^*J*_H,H_ = 12.84 Hz, C**H**_2_Ph), 4.16 (dq, 1H, ^5^J_H,H_ = 1.17 Hz, ^3^*J*_H,F_ = 6.42 Hz, C**H**), 5.20 (s, 1H, O**H**), 5.38 (d, 1H, ^5^J_H,H_ = 1.17 Hz, C**H**(OEt)_2_), 7.30–7.38 (m, 5 arom. **H**) ppm. ^13^C NMR (150 MHz, CDCl_3_): δ 15.05, 15.06 (2OCH_2_**C**H_3_), 60.1 (q, ^2^*J*_C,F_ = 31.5 Hz, **C**H), 61.1, 61.2 (2O**C**H_2_CH_3_), 62.2 (**C**H_2_Ph), 73.0 (**C**≡CCH(OEt)_2_), 85.1 (C≡**C**CH(OEt)_2_), 91.1 (**C**H(OEt)_2_), 122.9 (q, ^1^*J*_C,F_ = 279.0 Hz, **C**F_3_), 128.0, 128.6, 129.4 (5 arom. **C**H), 135.7 (1 arom. **C**) ppm. ^19^F NMR (565 MHz, CDCl_3_): δ −71.46 (d, 3F, ^3^*J*_F,H_ = 6.42 Hz, C**F**_3_) ppm. IR: *v* 3331*w* (O-H), 2978*w*, 2926*w*, 2859*w*, 1673*w*, 1454*w*, 1353*w*, 1271*m*, 1384*m*, 1177*s*, 1140*vs*, 1051*s*, 928*w*, 883*w*, 827*w* cm^−1^. HRMS (TOF AP^+^) *m*/*z* [M+H]^+^ calcd C_16_H_21_NO_3_F_3_: 332.1474, found: 332.1470; [M+Na]^+^ calcd C_16_H_2O_NO_3_F_3_Na: 354.1293, found: 354.1297.

*(b)*  *Enantioselective reactions*

*General procedure*. Under an argon atmosphere, THF (0.5 mL) was syringed into a round-bottomed flask with a rubber septum containing the AziPhenol ligand (6 mg, 0.01 mmol). A solution of diethylzinc (200 μL of 1.0 M solution in hexane, 0.02 mmol) was added dropwise at room temperature. The mixture was stirred at room temperature until the solution became slightly cloudy. Then 40 mg 4Å MS was added to the mixture and cooled to −20 °C for an additional 10 min before Et_2_Zn (1.5 mL of 1.0 M solution in hexane, 0.15 mmol) and the appropriate acetylene (0.15 mmol) in THF (0.5 mL) were added to the mixture. The nitrone (0.1 mmol) was dissolved in 1.0 mL of THF and added dropwise to the mixture and stirred at −20 °C. The progress of the reaction was controlled by TLC (SiO_2_; hexane: ethyl acetate 3:2) and the time of completion of the reaction was about 3h. Then, a saturated NH_4_Cl solution was added to the reaction mixture. The organic layer was separated, and the water layer was extracted with DCM (3 × 10 mL). Combined organic layers were dried over anhydrous Na_2_SO_4_ and filtrated. Next, the solvent was evaporated under reduced pressure. The crude mixture was purified by column chromatography (silica gel, hexane with ethyl acetate in gradient) to afford the corresponding products.

*N-Benzyl-N-(1,1,1-trifluoro-4-phenylbut-3-yn-2-yl)hydroxylamine* (**5aa**): 79:21 e.r.; ${\left[a\right]}_{D}^{22}$ = −24.8 (c 0.3, CHCl_3_); HPLC analysis: Chiralcel AD-H, Hexanes: *^i^*PrOH = 90:10, flow = 1.0 mL/min, retention time: 6.59 (minor), 7.39 (major) min, wavelength = 250 nm.

*N-Benzyl-*N*-[1,1,1-trifluoro-4-(4′-methoxyphenyl)but-3-yn-2-yl]hydroxylamine* (**5ab**): 80:20 e.r.; ${\left[a\right]}_{D}^{22}$ = −40.9 (c 0.3, CHCl_3_); HPLC analysis: Chiralcel AD-H, Hexanes: *^i^*PrOH = 95:5, flow = 1.0 mL/min, retention time: 18.54 (minor), 20.29 (major) min, wavelength = 250 nm.

*N-Benzyl-N-[1,1,1-trifluoro-4-(4′-chlorophenyl)but-3-yn-2-yl]hydroxylamine* (**5ac**): 74:26 e.r.; ${\left[a\right]}_{D}^{22}$ = −33.4 (c 0.3, CHCl_3_); HPLC analysis: Chiralcel AD-H, Hexanes: *^i^*PrOH = 95:5, flow = 1.0 mL/min, retention time: 12.65 (minor), 17.19 (major) min, wavelength = 250 nm.

*N-Benzyl-N-[1,1,1-trifluoro-4-(4′-trifluoromethylphenyl)but-3-yn-2-yl]hydroxylamine* (**5ad**): 72:28 e.r.; ${\left[a\right]}_{D}^{22}$ = −20.5 (c 0.3, CHCl_3_); HPLC analysis: Chiralcel AD-H, Hexanes: *^i^*PrOH = 95:5, flow = 1.0 mL/min, retention time: 13.38 (minor), 17.84 (major) min, wavelength = 250 nm.

*N-Benzyl-N-(1,1,1-trifluoro-5,5-dimethylheks-3-yn-2-yl)hydroxyloamine* (**5ae**): 71:29 e.r.; ${\left[a\right]}_{D}^{22}$ = −9.9 (c 0.3, CHCl_3_); HPLC analysis: Chiralcel AD, Hexanes: *^i^*PrOH = 98:2, flow = 0.5 mL/min, retention time: 12.55 (minor), 14.23 (major) min, wavelength = 250 nm.

*N-Benzyl-N-(1,1-difluoro-4-phenylbut-3-yn-2-yl)hydroxylamine* (**5ba**): 77:23 e.r.; ${\left[a\right]}_{D}^{22}$ = −19.2 (c 0.3, CHCl_3_); HPLC analysis: Chiralcel AD-H, Hexanes: *^i^*PrOH = 90:10, flow = 0.7 mL/min, retention time: 12.55 (minor), 14.23 (major) min, wavelength = 250 nm.

*2-Benzyl-5-phenyl-3-(trifluoromethyl)-2,3-dihydroisoxazole* (**6aa**): 50:50 e.r.; HPLC analysis: Chiralcel AD, Hexanes: *^i^*PrOH = 90:10, flow = 0.5 mL/min, retention time: 9.36, 10.14 min, wavelength = 250 nm.

*2-Benzyl-5-(4′-methoxyphenyl)-3-(trifluoromethyl)-2,3-dihydroizoxazole* (**6ab**): 51:49 e.r.; HPLC analysis: Chiralcel AD, Hexanes: *^i^*PrOH = 95:5, flow = 0.5 mL/min, retention time: 13.23, 14.15 min, wavelength = 250 nm.

*2-Benzyl-5-(4′-chlorophenyl)-3-(trifluoromethyl)-2,3-dihydroizoxazole* (**6ac**): 53:47 e.r.; HPLC analysis: Chiralcel AD, Hexanes: *^i^*PrOH = 98:2, flow = 0.3 mL/min, retention time: 10.65, 11.24 min, wavelength = 250 nm.

*2-Benzyl-5-(4′-trifluoromethylphenyl)-3-(trifluoromethyl)-2,3-dihydroizoxazole* (**6ad**): 51:49 e.r.; HPLC analysis: Chiralcel AD, Hexanes: *^i^*PrOH = 95:5, flow = 0.5 mL/min, retention time: 5.40, 5.78 min.

### 3.4. Computational Study

All calculations reported in this work were performed using the “Ares” cluster in the “Cyfronet” computational center in Cracow. The wb97xd functional and the 6-311+G(d) basis set included in the GAUSSIAN 09 package [37] were used. For all structures optimization the Berny algorithm was applied. Localized critical points were checked by vibrational frequency analyses to see whether they constituted minima or maxima on the potential energy surface (PES). All transition structures (TSs) showed a single imaginary frequency, whereas reactants, products and pre-reaction complexes had none. The intrinsic reaction coordinate (IRC) path was traced in order to check the energy profiles connecting each transition structure to the two associated minima of the proposed mechanism. GEDT indices were estimated according to Domingo equations [38]. The reaction environment polarity (THF) was simulated using polarizable continuum model (PCM) [39].

## 4. Conclusions

A method of synthesis of fluorinated hydroxylamines of type **5** was developed by adding acetylenes **4** to nitrones **3** derived from fluorinated aldehydes **1**. In these reactions, the formation of hydroxylamines **5** and/or oxazolines **6** was observed as products. However, the expected hydroxylamines **5** were the only or the main products in the reactions conducted in the presence of diethylzinc. Theoretical calculations clearly indicate that oxazolines **6** are the kinetic products and hydroxylamines **5** are the thermodynamic products of the studied process. In the extension of the studies the first enantioselective protocol of addition of acetylenes **4** to RF-nitrones **3** was elaborated. The desired products **5** were isolated in good yields and with moderate enantioselectivities. The obtained hydroxylamines **5** consist of an interesting class of building blocks for use in organic synthesis. In the literature, propargyl *N*-hydroxylamines have been used for acylaziridines [10], propargylamines by reduction [40,41,42], α,β-unsaturated ketones by the sequence of isomerization and hydrolysis reactions [7].

## Figures and Tables

**Table 1:** Effects of ligand and metal reagent on the asymmetric additions of phenylacetylene **4a** to nitrone **3a** under the indicated conditions ^a^.

Entry	Ligand	M	Yield [%] ^b^	e.r. [%] ^c^
1	L1	Et_2_Zn	51	54:46
2	L2	Et_2_Zn	58	59:41
3	L3	Et_2_Zn	38	51:49
4	L4	Et_2_Zn	60	57:43
5	L5	Et_2_Zn	67	72:28
6 ^d^	L5	Zn(OTf)_2_	21	52:48

^a^ All reactions were carried out with **3a** (0.1 mmol), **4a** (0.15 mmol), ligand (0.01 mmol) and Et_2_Zn (0.17 mmol) in THF (2.0 mL) under nitrogen at 20 °C for 3 h. ^b^ Isolated yield after silica gel chromatography. ^c^ Determined by chiral HPLC. ^d^ Zn(OTf)_2_ (0.05 mmol).

**Table 2:** Screening Conditions for Reaction in the Presence of Ligand L5 ^a^.

Entry	Ligand (mol%)	Solvent	Time [h]	T [°C]	Yield [%] ^c^	e.r. [%] ^d^
1	10	THF	3	20	67	72:28
2	10	DCM	3	20	56	64:36
3	10	Toluene	3	20	49	59.5:40.5
4	10	Et_2_O	3	20	23	55:45
5	10	THF	4	0	70	74:26
6	10	THF	4	−20	76	76.5:23.5
7	10	THF	4	−78	68	75:25
8	20	THF	4	−20	73	76:24
9	5	THF	5	−20	77	73.5:26.5
10 ^b^	10	THF	4	−20	80	79:21

^a^ Unless otherwise noted, all reactions were carried out with **3a** (0.1 mmol), **4a** (0.15 mmol), ligand (0.01 mmol) and Et_2_Zn (0.17 mmol) in THF (2.0 mL) under nitrogen at for 3 h. ^b^ 40 mg of 4 Å MS were added. ^c^ Isolated yield after column chromatography. ^d^ Determined by chiral HPLC.

## Data Availability

Data are contained within the article.

## Supplementary Materials

The following supporting information can be downloaded at: https://www.mdpi.com/article/10.3390/molecules30234578/s1. Copies of ^1^H and ^13^C spectra of all new compounds, HPLC chromatograms and crystallographic data [43,44,45,46]. Figure S1: Molecular structure of **5aa** (left) and **3a** (right). Displacement ellipsoids are drawn at 50% probability level; Figure S2: Partial packing diagram of **5aa** (left) and **3a** (right). Displacement ellipsoids are drawn at 50% probability level; Figure S3: Selected interactions stabilizing molecular and supramolecular structure of **5aa**; Figure S4: Selected interactions stabilizing molecular and supramolecular structure of **3a**. Table S1: Selected structural data for 5aa and 3a; Table S2: Bond Lengths for **3a**_DEPO; Table S3: Bond Angles for **5aa**_DEPO; Table S4: Bond Angles for **3a**_DEPO; Table S5: Torsion Angles for 5aa_DEPO; Table S6: Torsion Angles for 3a_DEPO.
