# Supplementary material for: Competition of the Addition/Cycloaddition Schemes in the Reaction Between Fluorinated Nitrones and Arylacetylenes: Comprehensive Experimental and DFT Study
Source: Molecules. 2025 Nov 28;30(23):4578. doi: 10.3390/molecules30234578 (PMC12692762; doi:10.3390/molecules30234578)
Supplement: Supplementary file 1 [file molecules-30-04578-s001.zip › molecules-3975697-supplementary.pdf]

Supporting information

for

Competition of the Addition/Cycloaddition Schemes in the Reaction Between Fluorinated Nitrones and Arylacetylenes: Comprehensive Experimental and DFT Study

Szymon Jarzyński [1], Andrzej Krempiński [2], Anna Pietrzak [3], Radomir Jasiński [4,*] and Emilia Obijalska [2,*]

[1] Faculty of Chemistry, Department of Organic Chemistry, University of Lodz, Tamka 12, 91-403 Lodz, Poland; szymon.jarzynski@chemia.uni.lodz.pl
[2] Faculty of Chemistry, Department of Organic and Applied Chemistry, University of Lodz, 91-403 Lodz, Poland; andrzej.krempinski@edu.uni.lodz.pl
[3] Institute of General and Ecological Chemistry, Lodz University of Technology, Żeromskiego 116, 90-924 Lodz, Poland; anna.pietrzak.1@p.lodz.pl
[4] Cracow University of Technology, Department of Organic Chemistry and Technology, Warszawska 24, 31-155 Kraków, Poland
* Correspondence: radomir.jasinski@pk.edu.pl (R.J.); emilia.obijalska@chemia.uni.lodz.pl (E.O.)

Table of contents **Page**

1. Copies of ^{1}H-NMR and ^{13}C-NMR spectra 2
2. HPLC analyses 13
3. Crystallographic data

1. Copies of ^{1}H-NMR, ^{13}C-NMR, ^{19}F-NMR and IR spectra

N-Benzyl-N-(1,1,1-trifluoro-4-phenylbut-3-yn-2-yl)hydroxylamine (**5aa**).

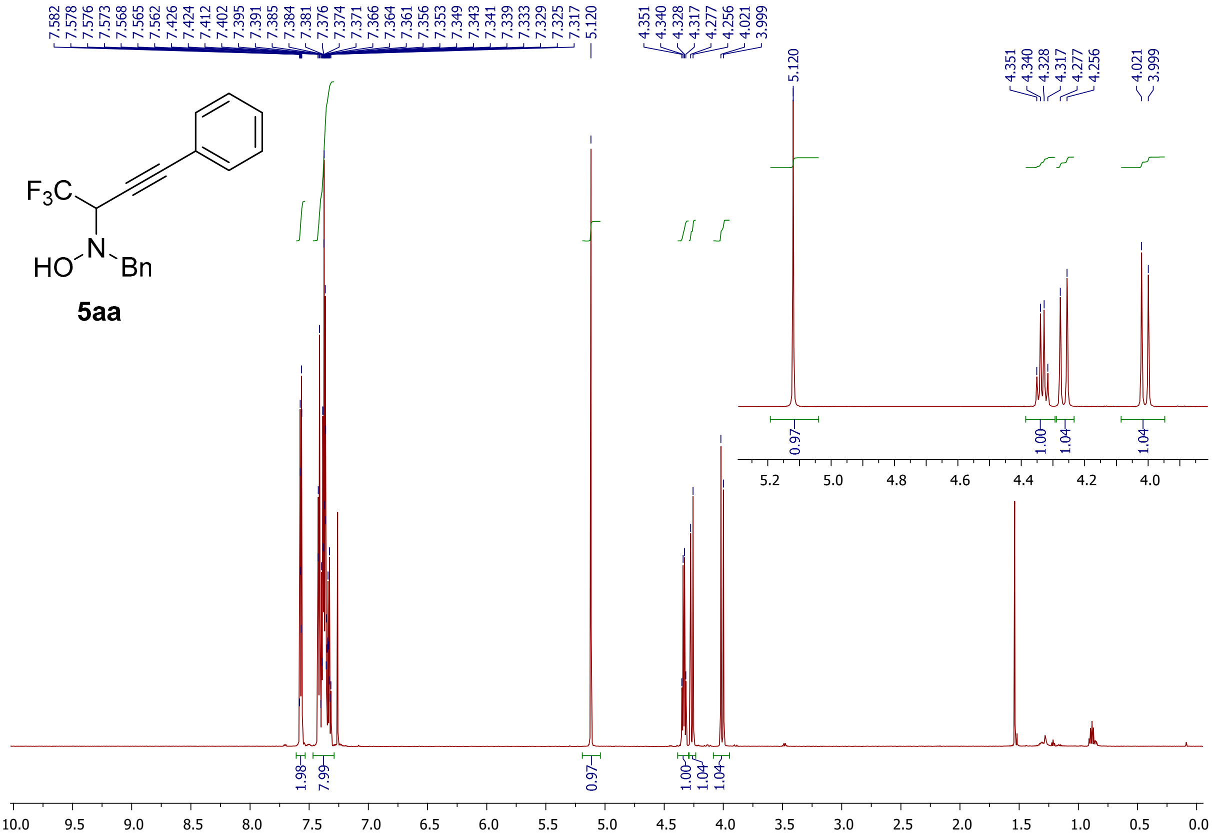

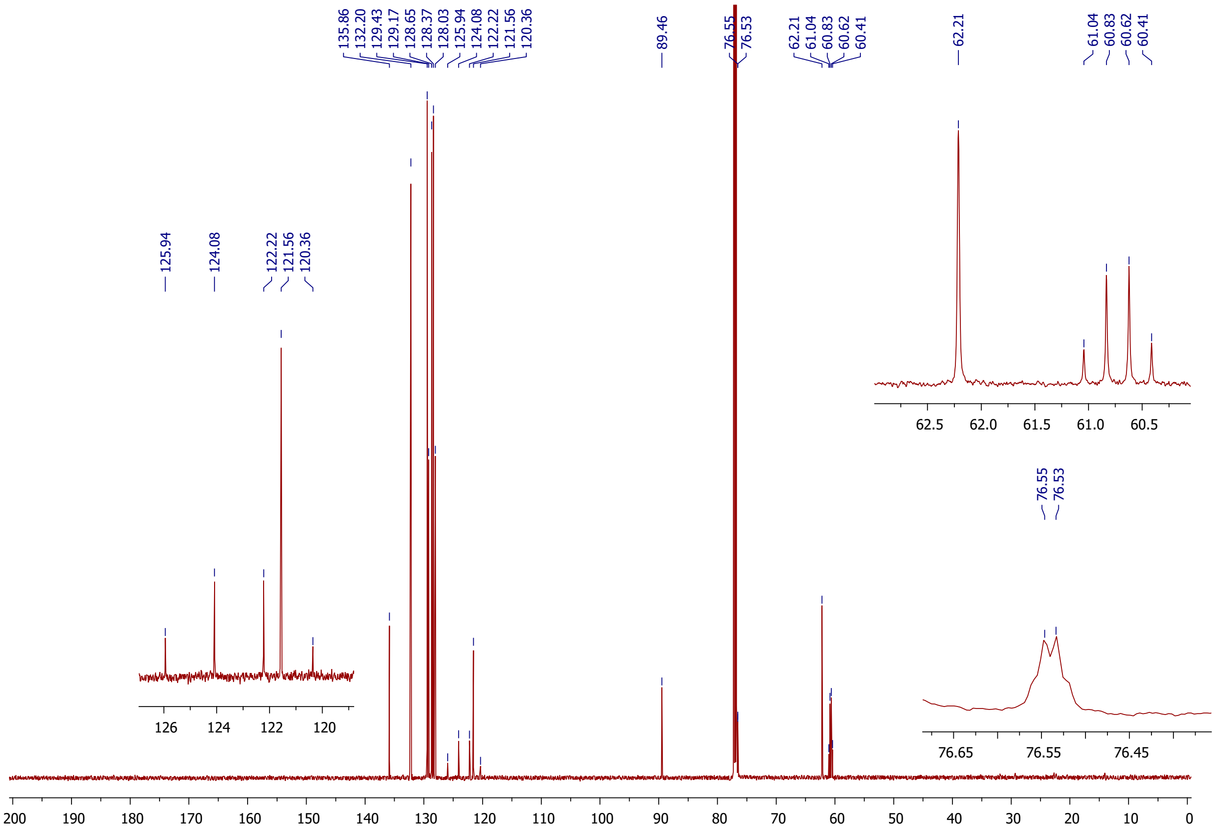

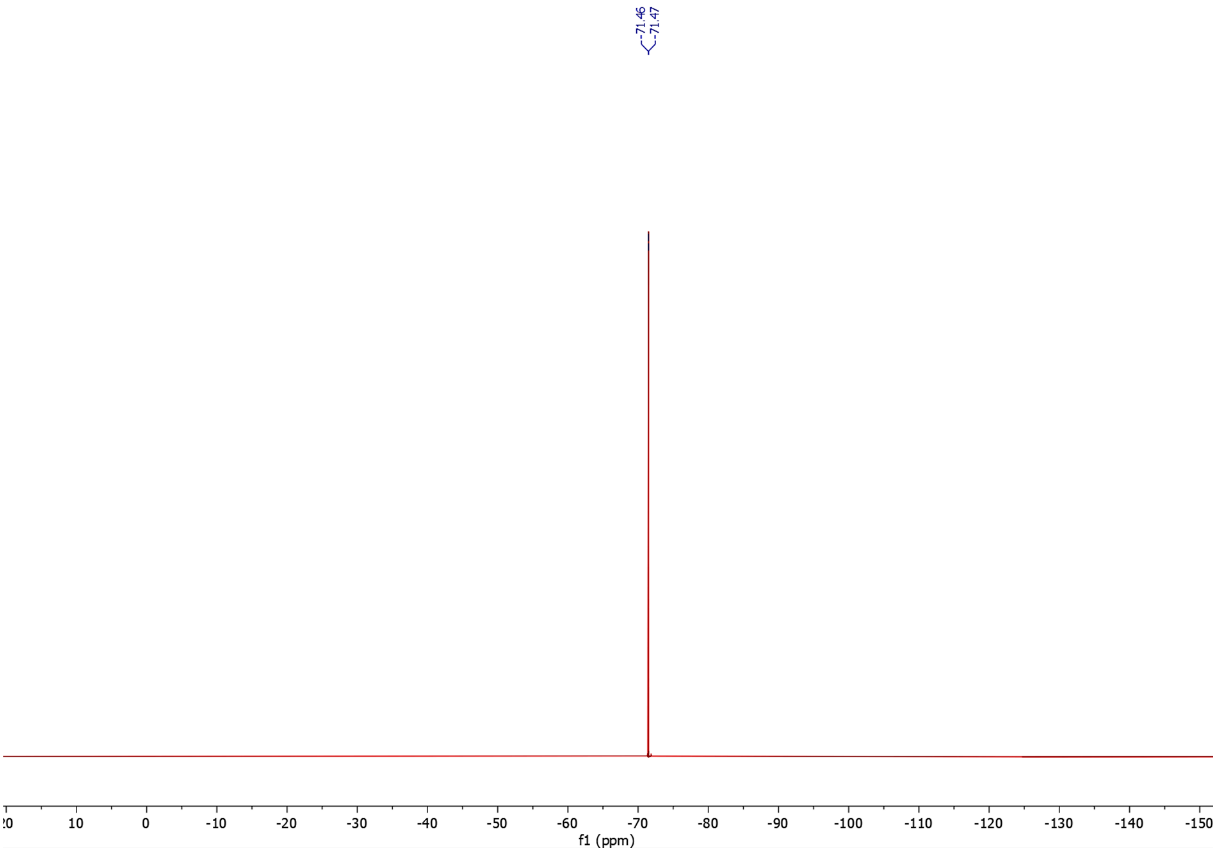
-71.46
-71.47
20
10
0
-10
-20
-30
-40
-50
-60
-70
-80
-90
-100
-110
-120
-130
-140
-150
f1 (ppm)

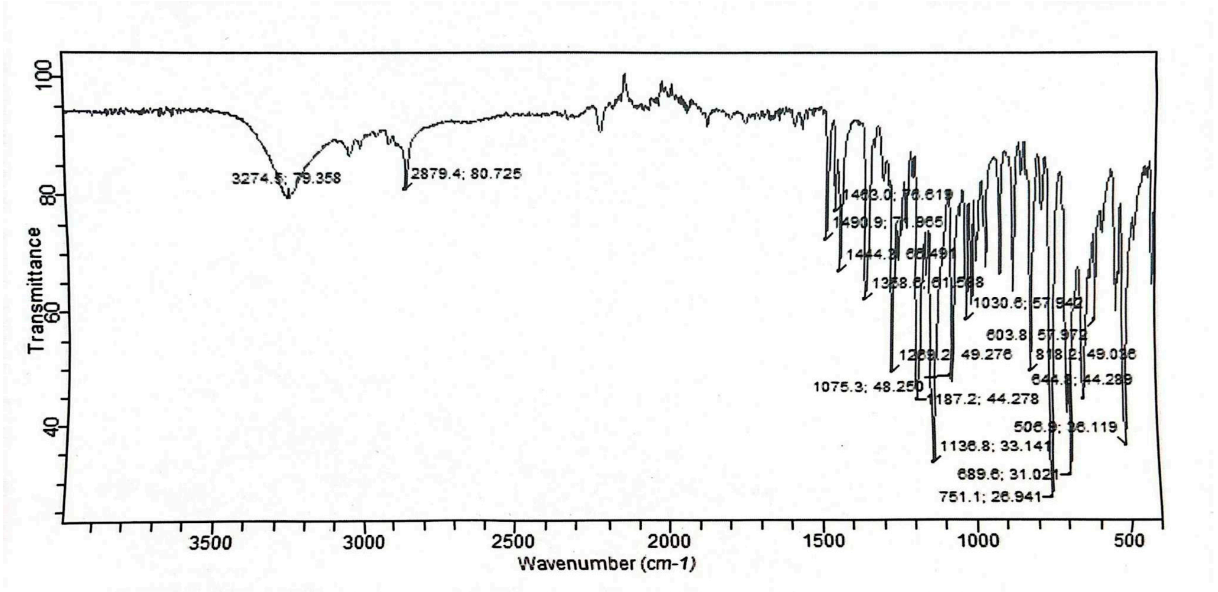
Transmittance
100
80
60
40
2879.4; 80.725
1075.3; 48.250
1187.2; 44.278
1136.8; 33.141
689.6; 31.021
751.1; 26.941
3500
3000
2500
2000
1500
1000
500
Wavenumber (cm-1)

N-Benzyl-N-(1,1-difluoro-4-phenylbut-3-yn-2-yl)hydroxylamine (**5ba**).

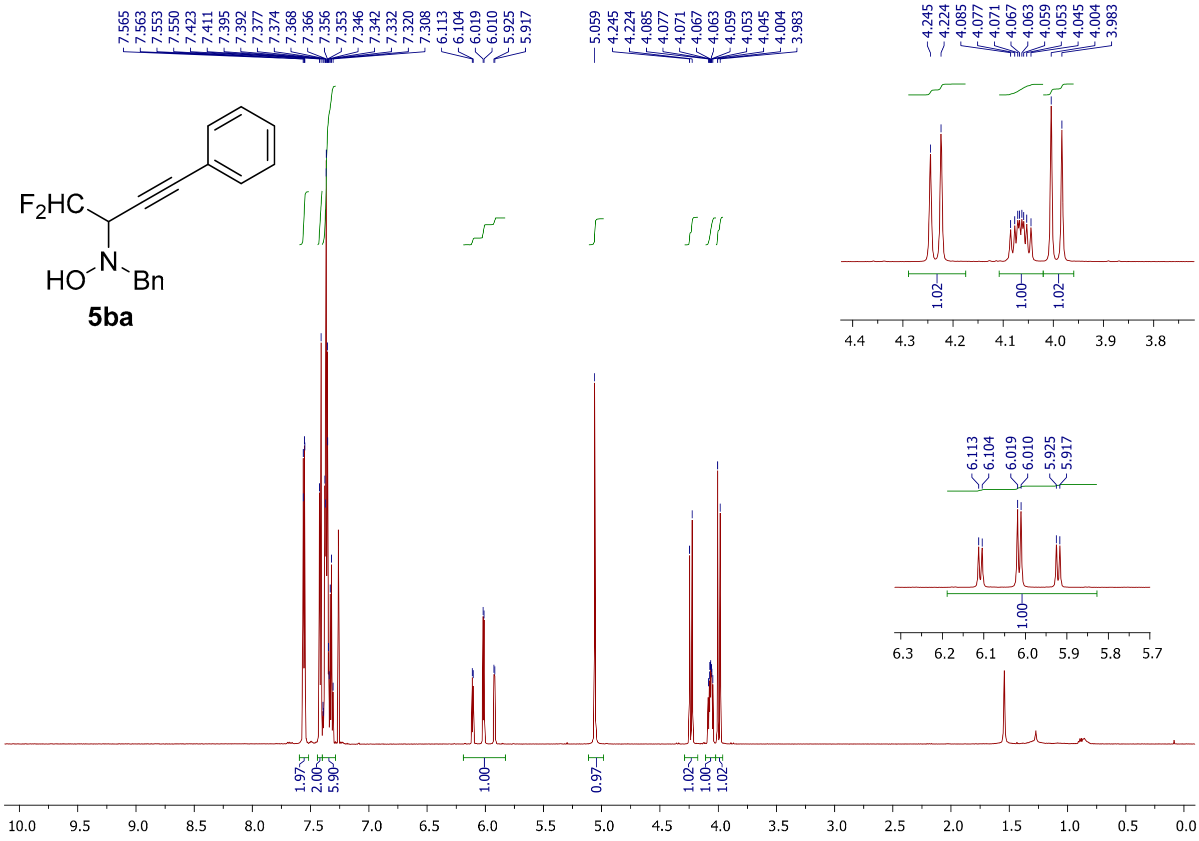

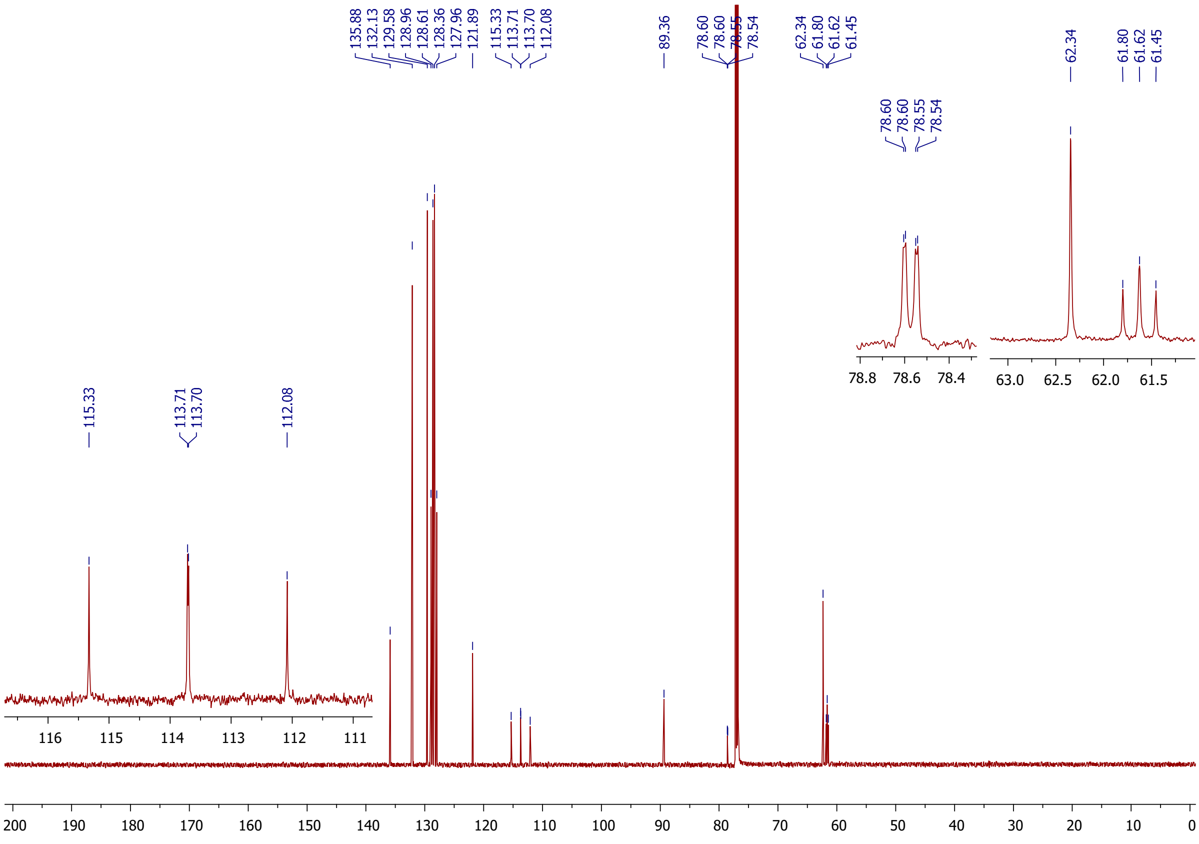

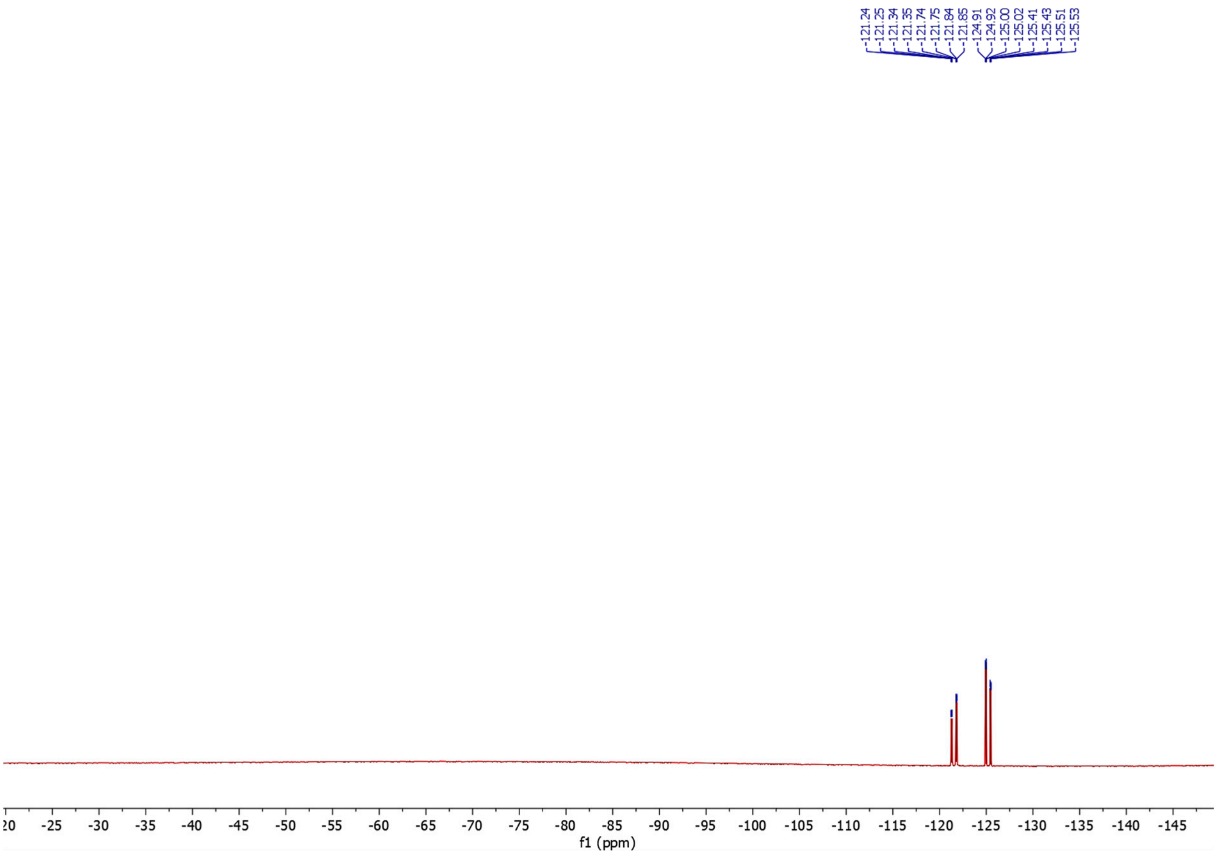
-121.24
-121.25
-121.34
-121.35
-121.74
-121.75
-121.84
-121.85
-124.91
-124.92
-125.00
-125.02
-125.41
-125.43
-125.51
-125.53
f1 (ppm)

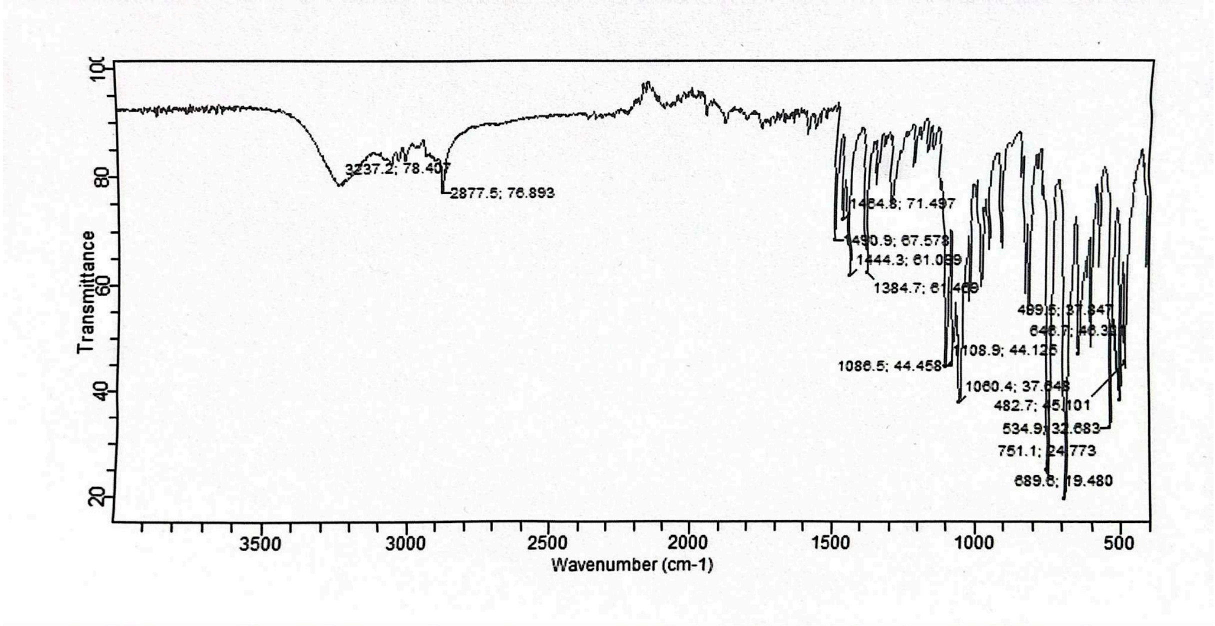
Transmittance
Wavenumber (cm-1)
2877.5; 76.893
1086.5; 44.458
1108.9; 44.125
482.7; 45.101
751.1; 24.773

N-Benzyl-N-[1,1,1-trifluoro-4-(4′-methoxyphenyl)but-3-yn-2-yl]hydroxylamine (**5ab**).

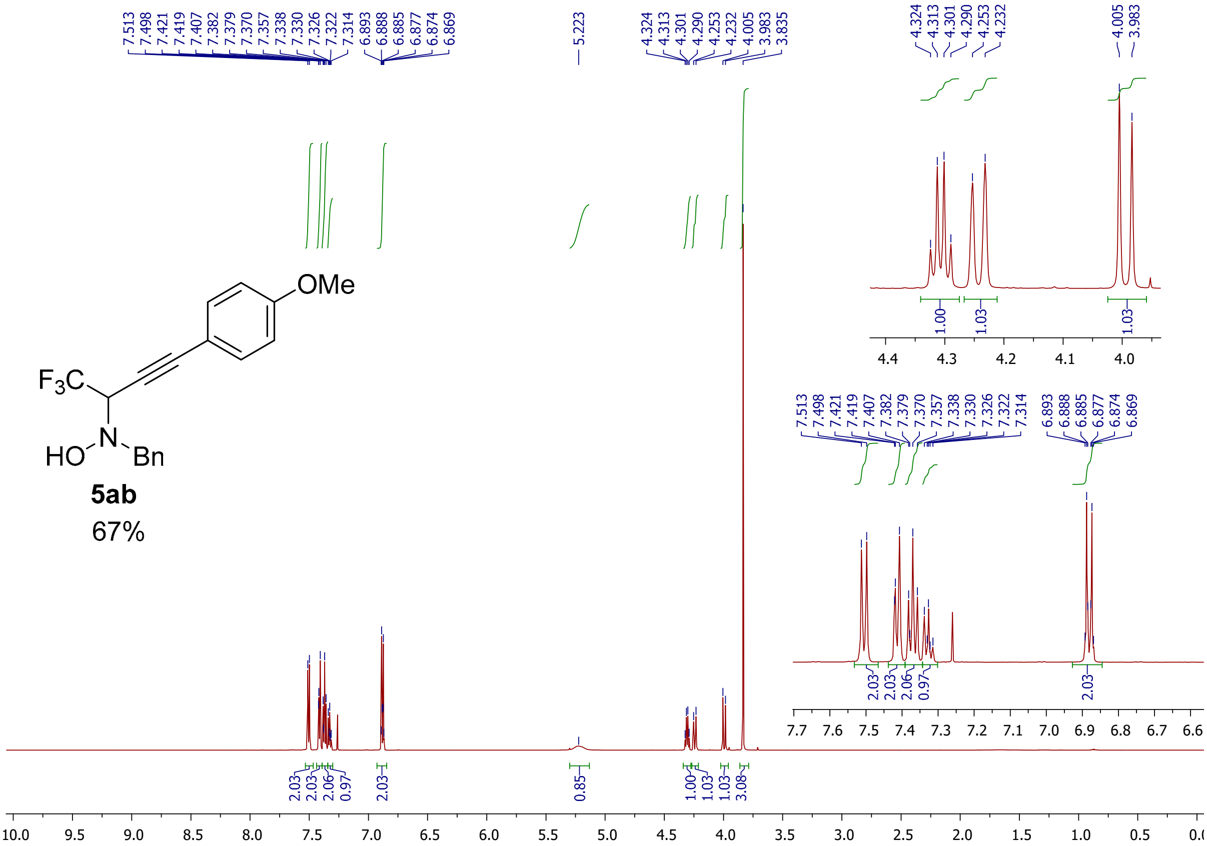

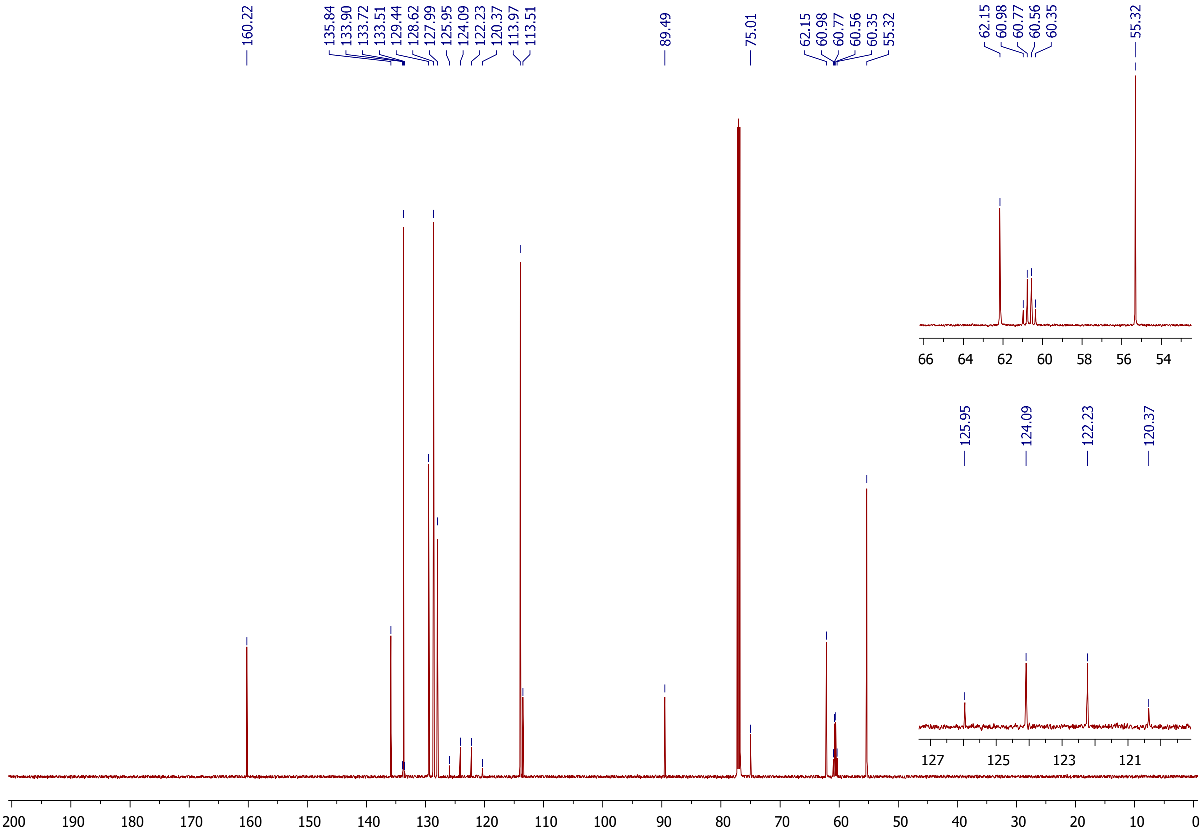

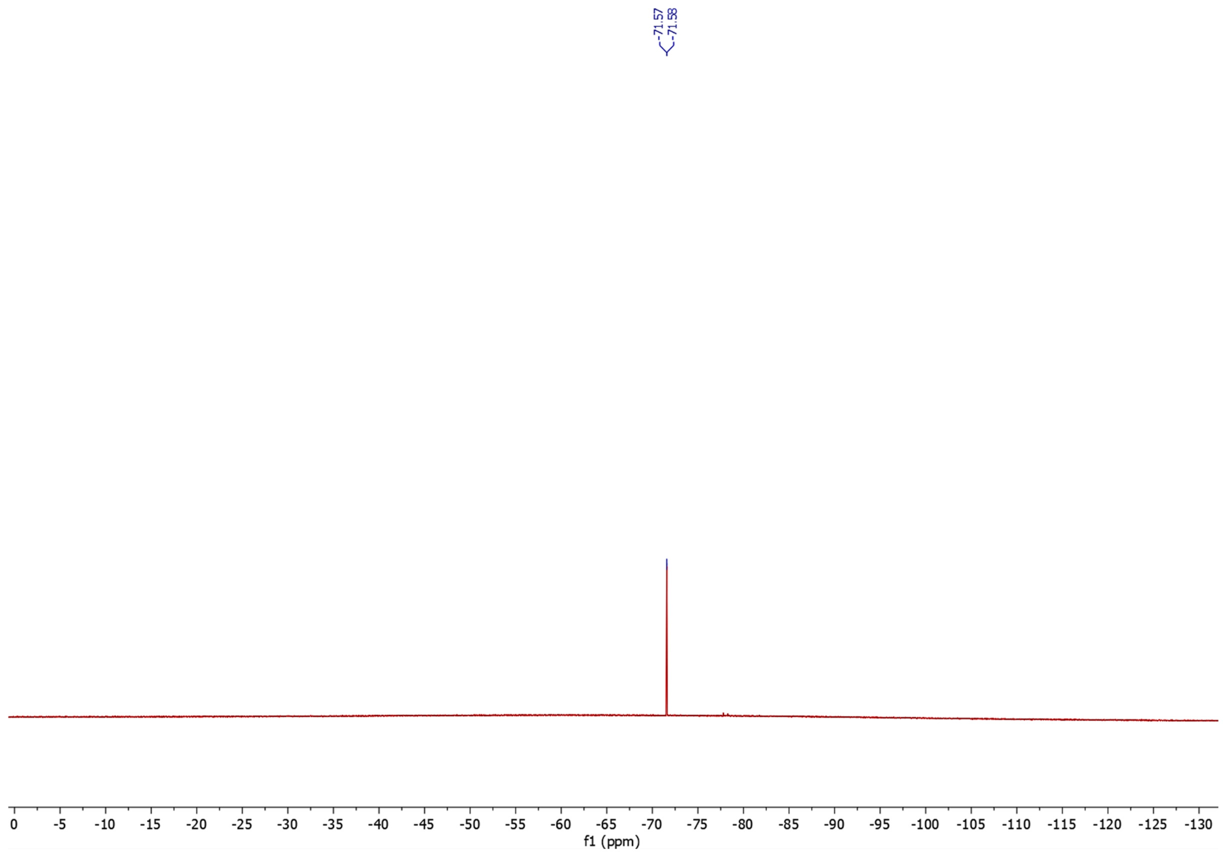
-71.57
-71.58
0 -5 -10 -15 -20 -25 -30 -35 -40 -45 -50 -55 -60 -65 -70 -75 -80 -85 -90 -95 -100 -105 -110 -115 -120 -125 -130
f1 (ppm)

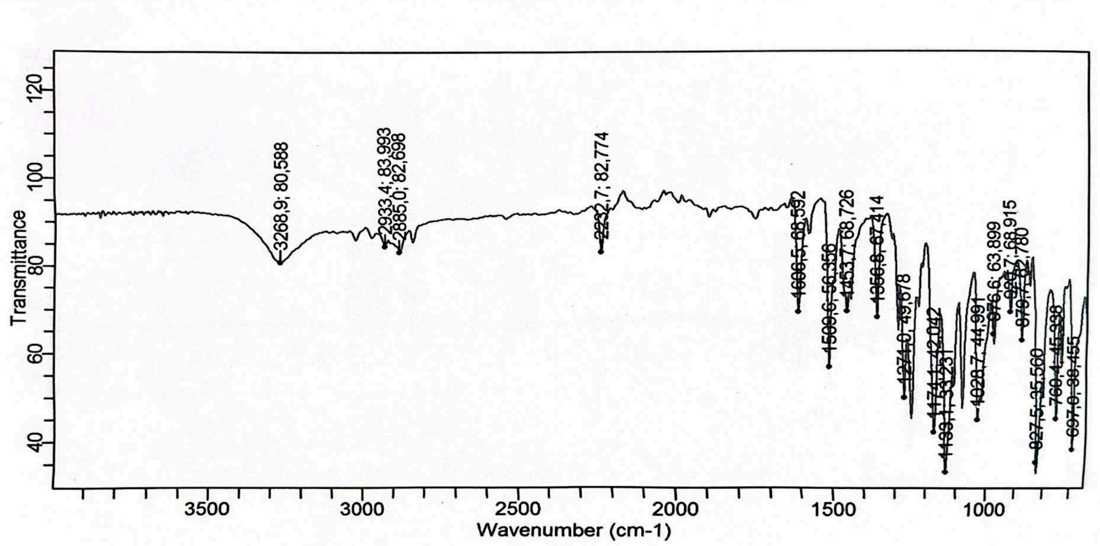
Transmittance
40
60
80
100
120
3268,9; 80,588
2933,4; 83,993
2885,0; 82,698
2232,7; 82,774
3500
3000
2500
2000
1500
1000
Wavenumber (cm-1)

2-Benzyl-5-(4′-methoxyphenyl)-3-(trifluoromethyl)-2,3-dihydroizoxazole (**6ab**).

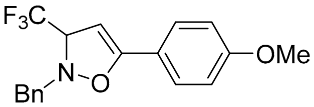

6ab

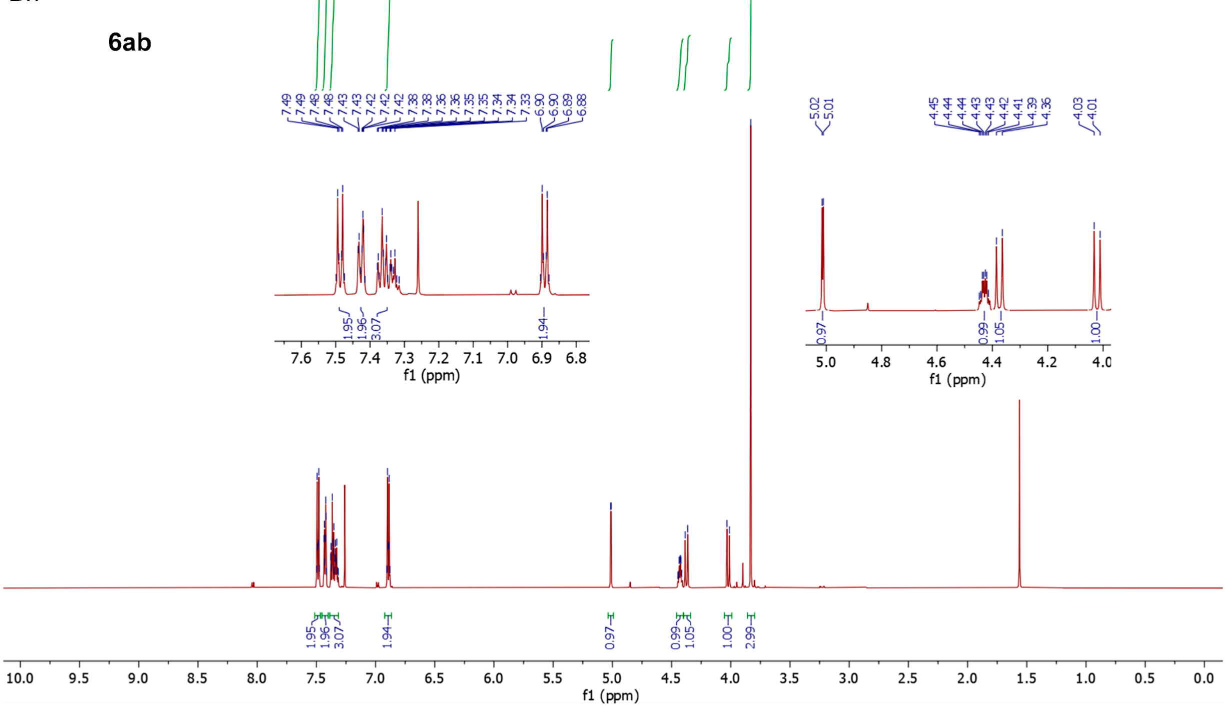

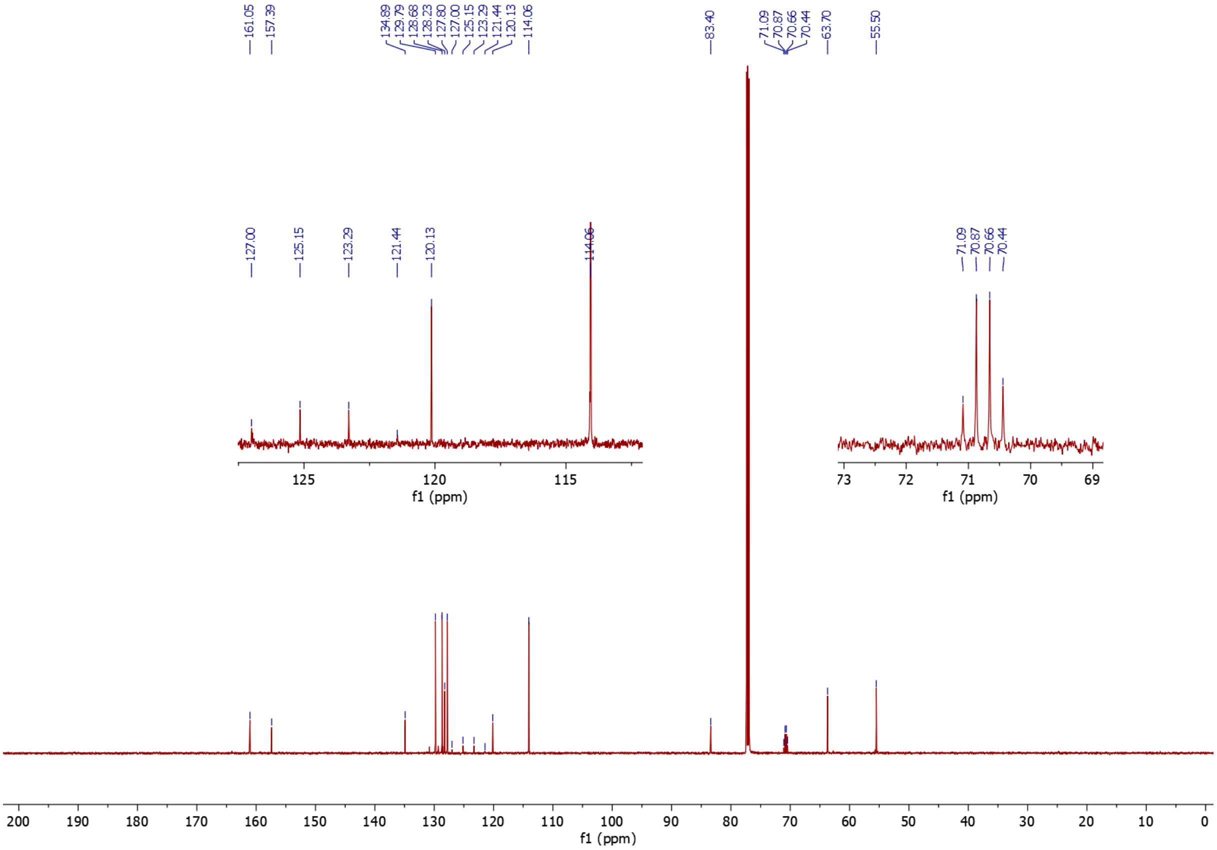

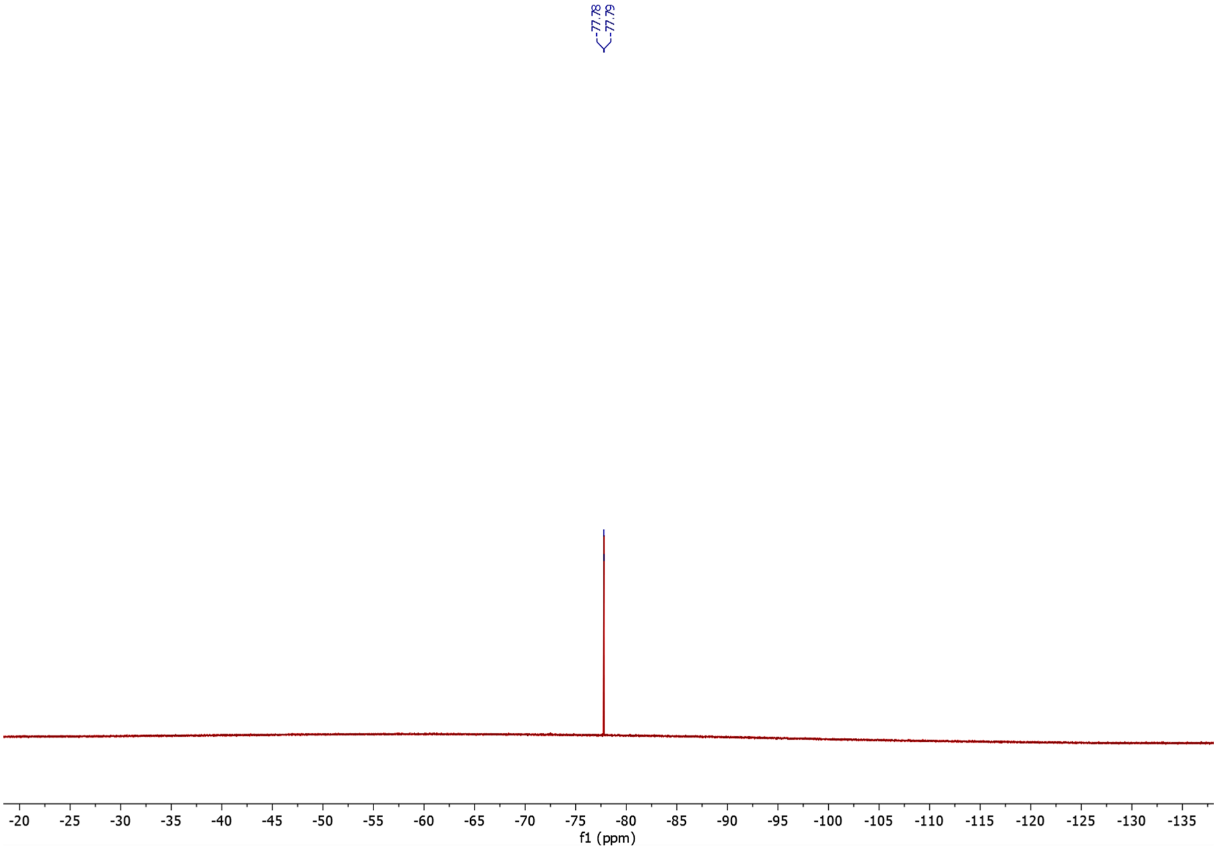
-77.78
-77.79
-20 -25 -30 -35 -40 -45 -50 -55 -60 -65 -70 -75 -80 -85 -90 -95 -100 -105 -110 -115 -120 -125 -130 -135
f1 (ppm)

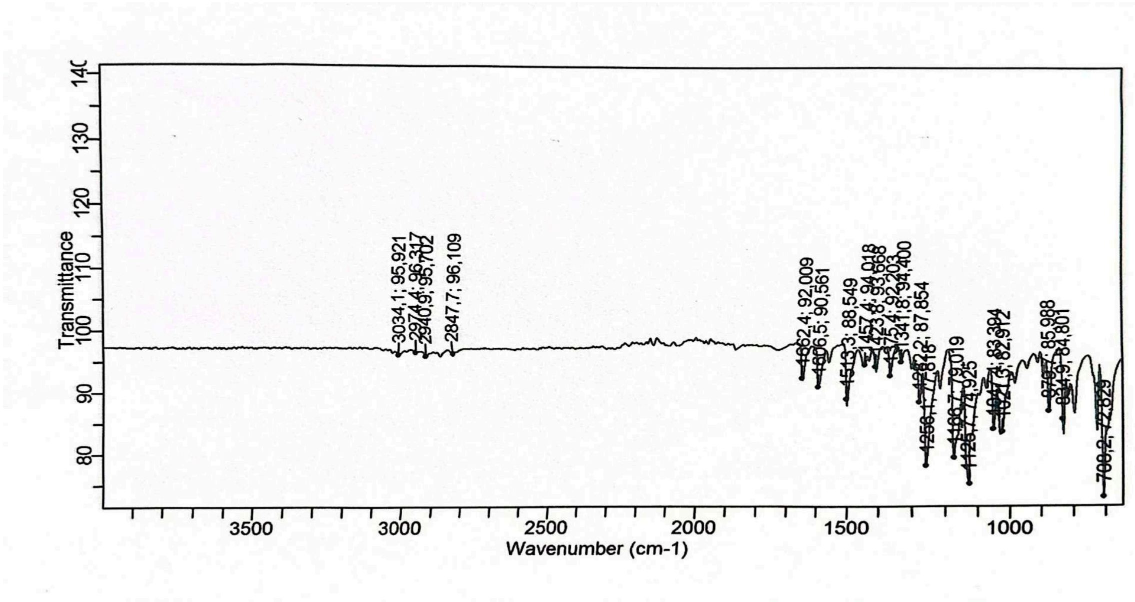
Transmittance
Wavenumber (cm-1)
3034,1; 95,921
2974,4; 96,317
2940,9; 95,702
2847,7; 96,109
1606,5; 90,561
1513,3; 88,549
1341,8; 94,400
700,2; 72,629

N-Benzyl-N-[1,1,1-trifluoro-4-(4′-chlorophenyl)but-3-yn-2-yl]hydroxylamine (**5ac**).

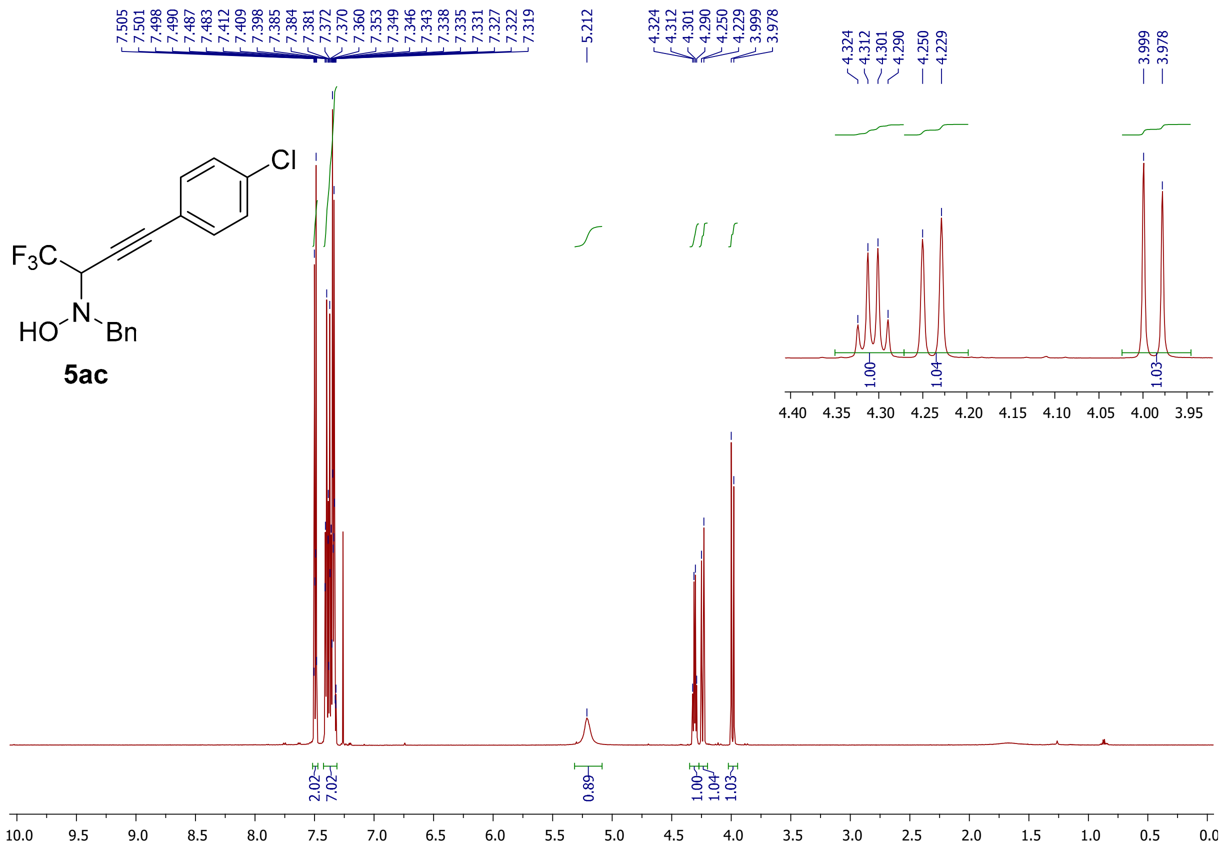

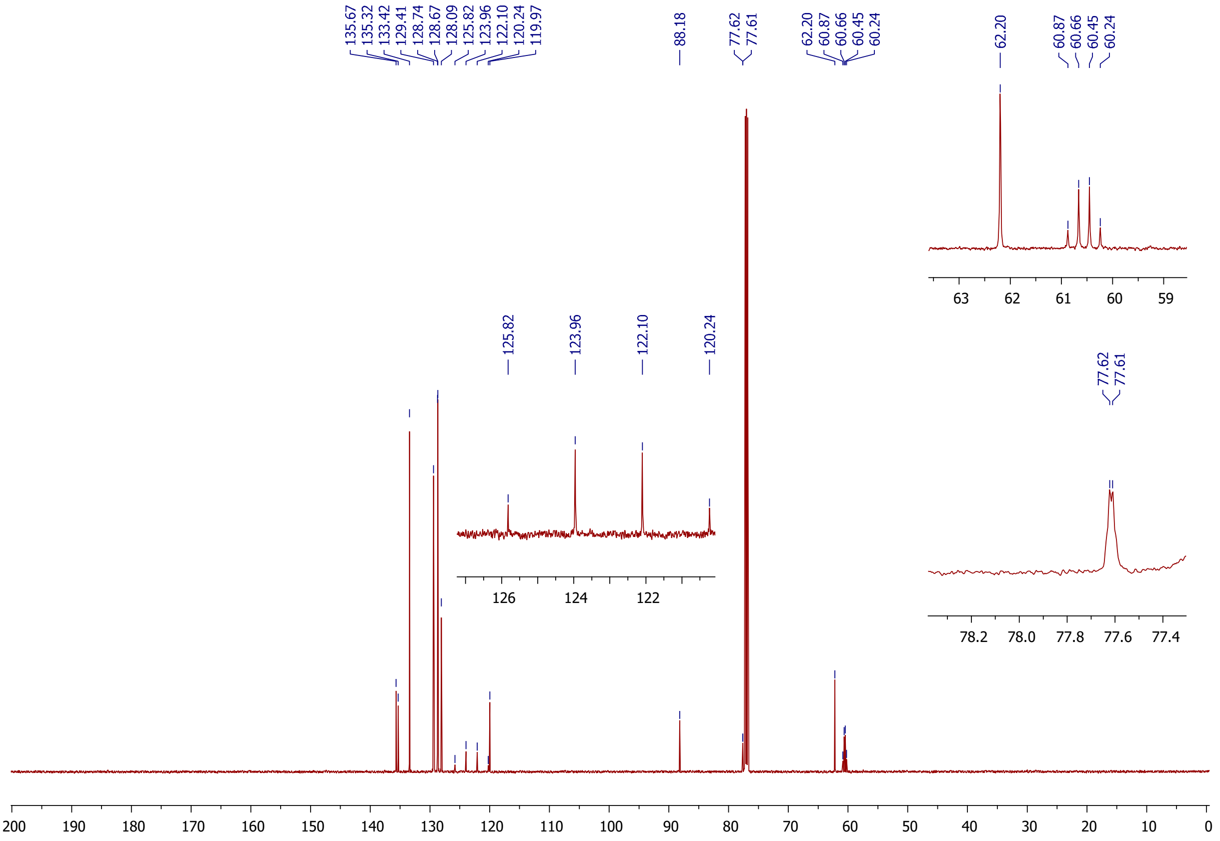

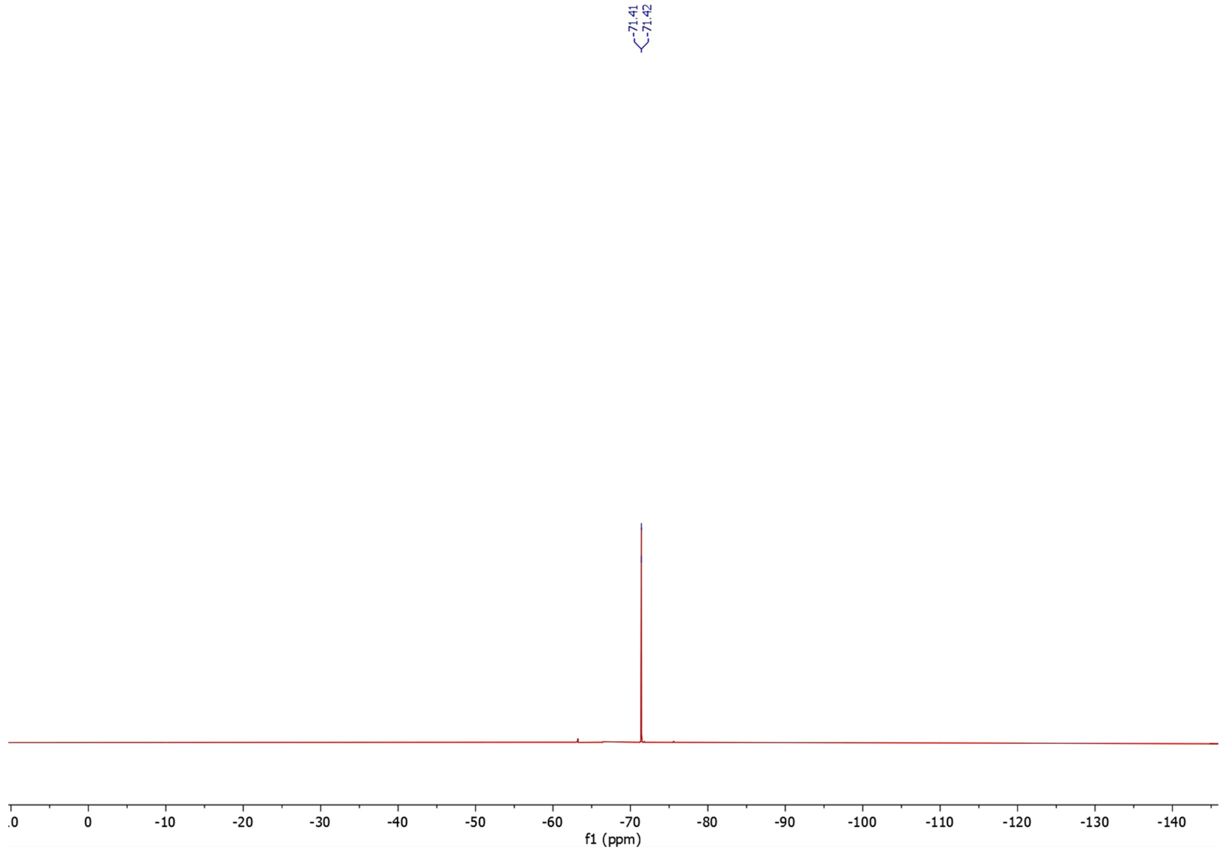
-71.41
-71.42
f1 (ppm)

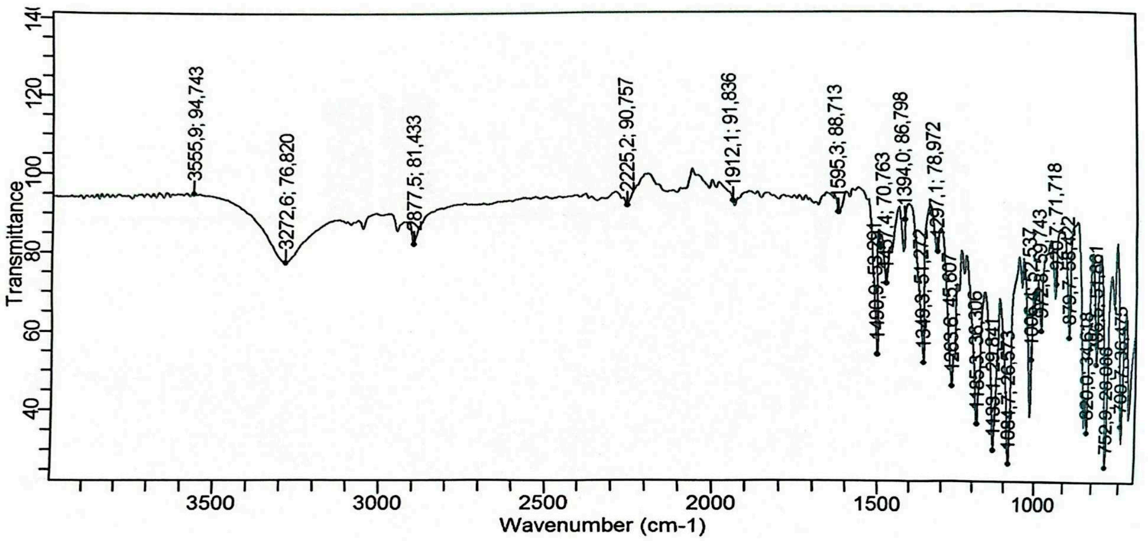
3555,9; 94,743
3272,6; 76,820
2877,5; 81,433
2225,2; 90,757
1912,1; 91,836
1595,3; 88,713
1394,0; 86,798
1297,1; 78,972
71,718
Transmittance
Wavenumber (cm-1)

2-Benzyl-5-(4′-chlorophenyl)-3-(trifluoromethyl)-2,3-dihydroizoxazole (**6ac**).

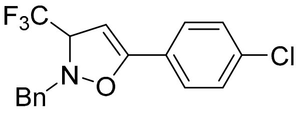

6ac

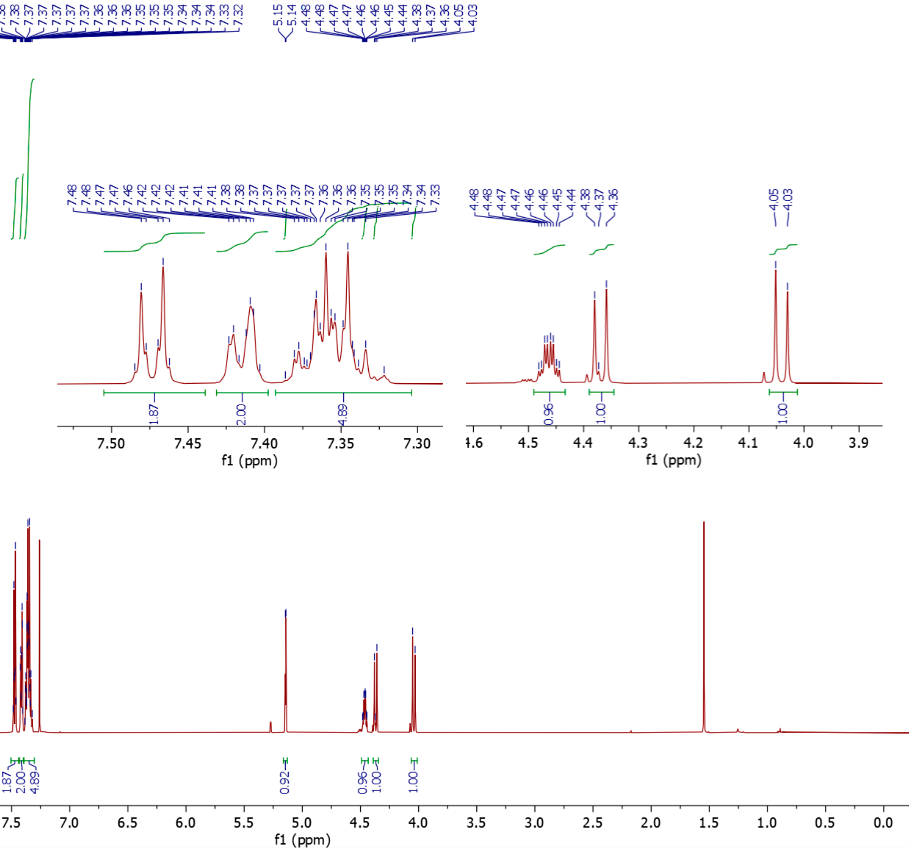

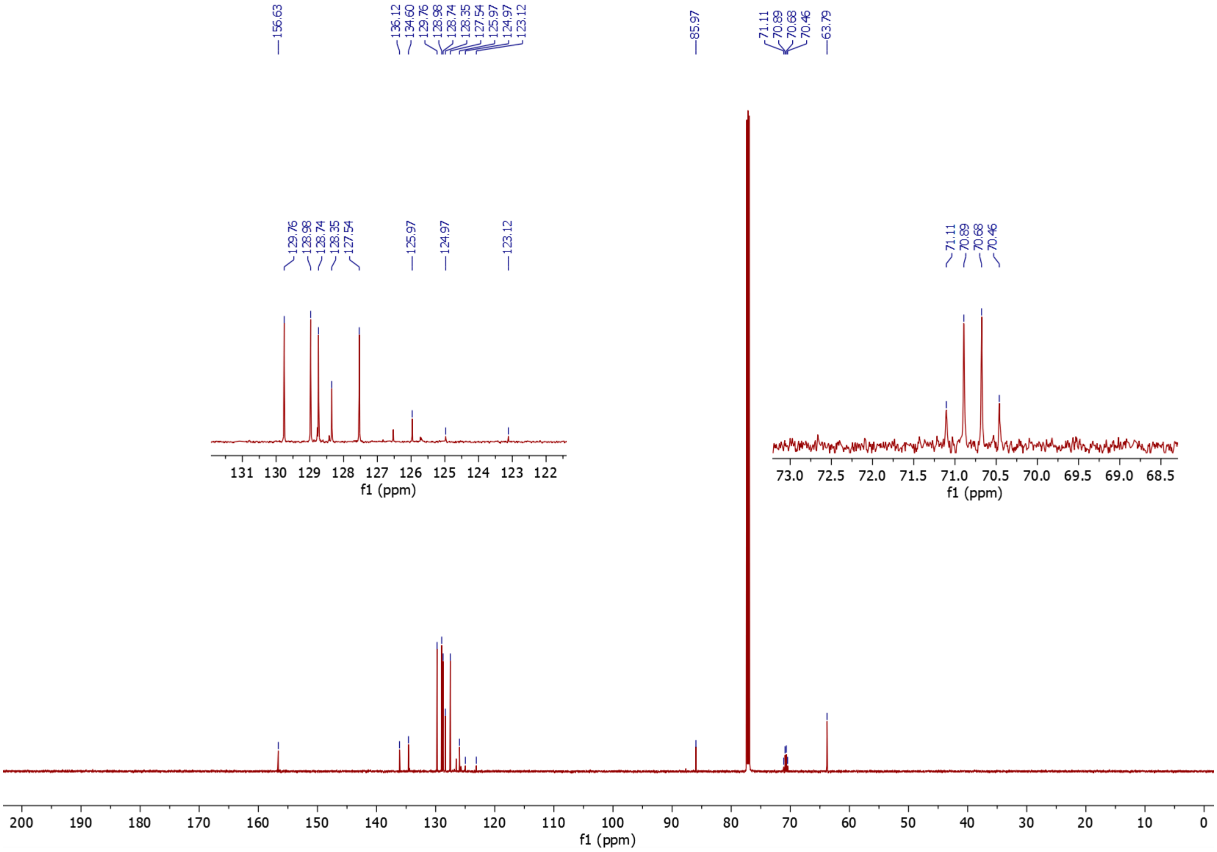

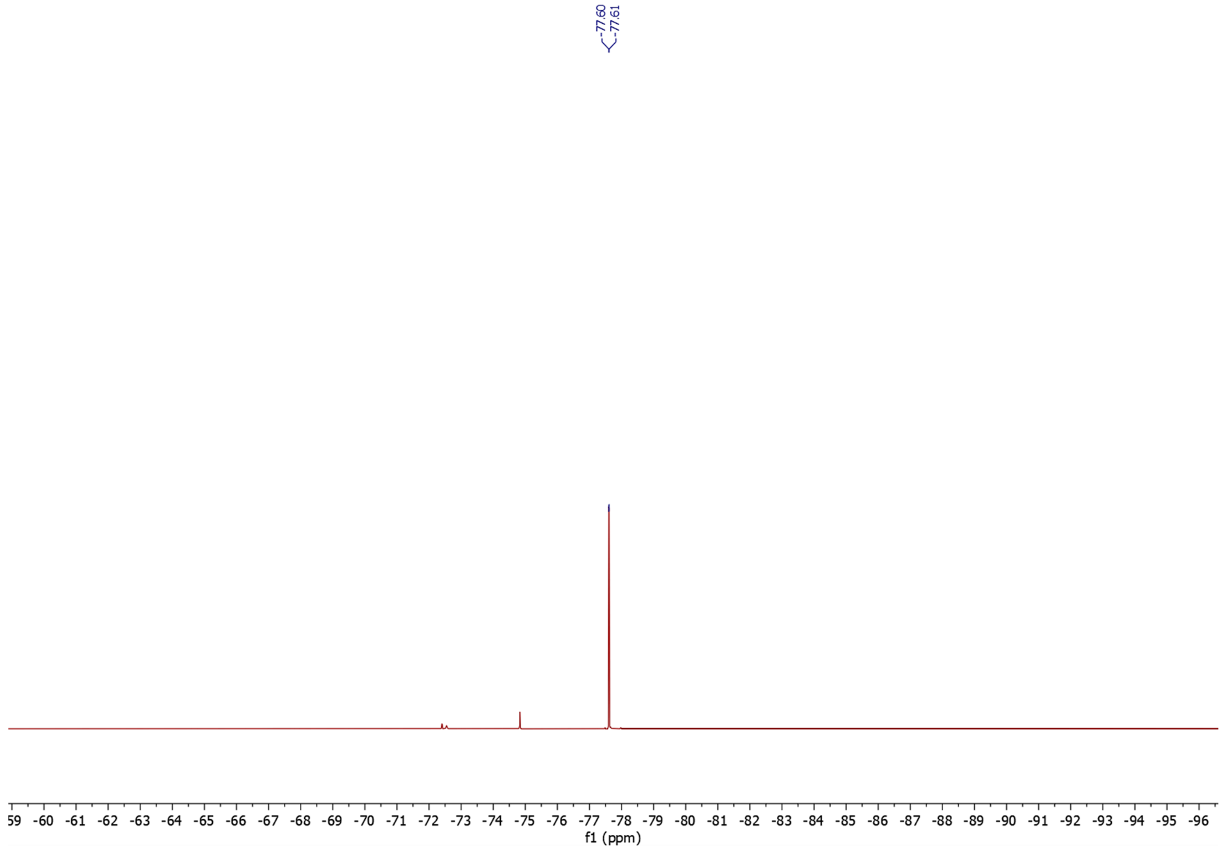
-77.60
-77.61
59 -60 -61 -62 -63 -64 -65 -66 -67 -68 -69 -70 -71 -72 -73 -74 -75 -76 -77 -78 -79 -80 -81 -82 -83 -84 -85 -86 -87 -88 -89 -90 -91 -92 -93 -94 -95 -96
f1 (ppm)

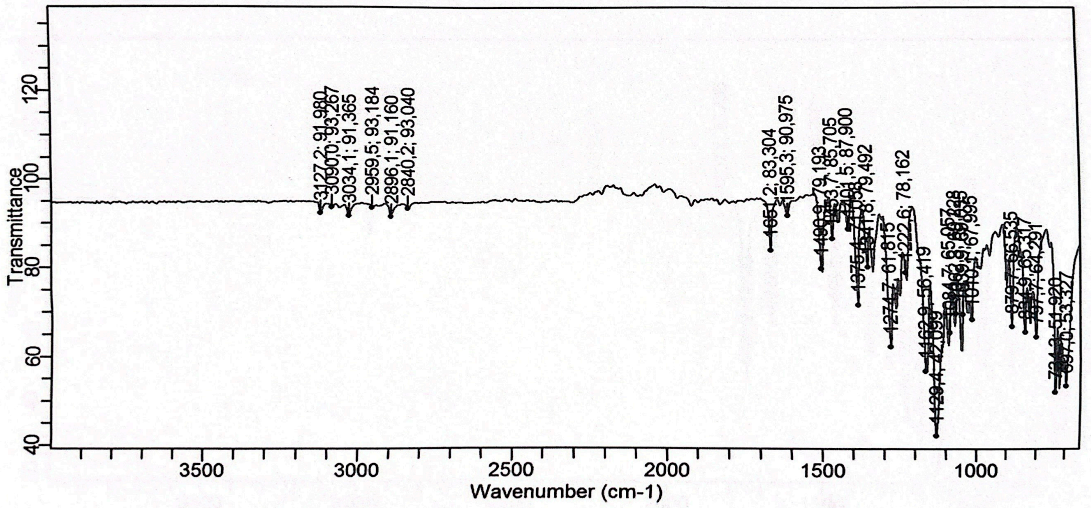
Transmittance
40
60
80
100
120
3500
3000
2500
2000
1500
1000
Wavenumber (cm-1)
3127,2; 91,980
3034,1; 91,365
2959,5; 93,184
2896,1; 91,160
2840,2; 93,040
1595,3; 90,975
1222,6; 78,162

N-Benzyl-N-[1,1,1-trifluoro-4-(4′-trifluoromethylphenyl)but-3-yn-2-yl]hydroxylamine (**5ad**).

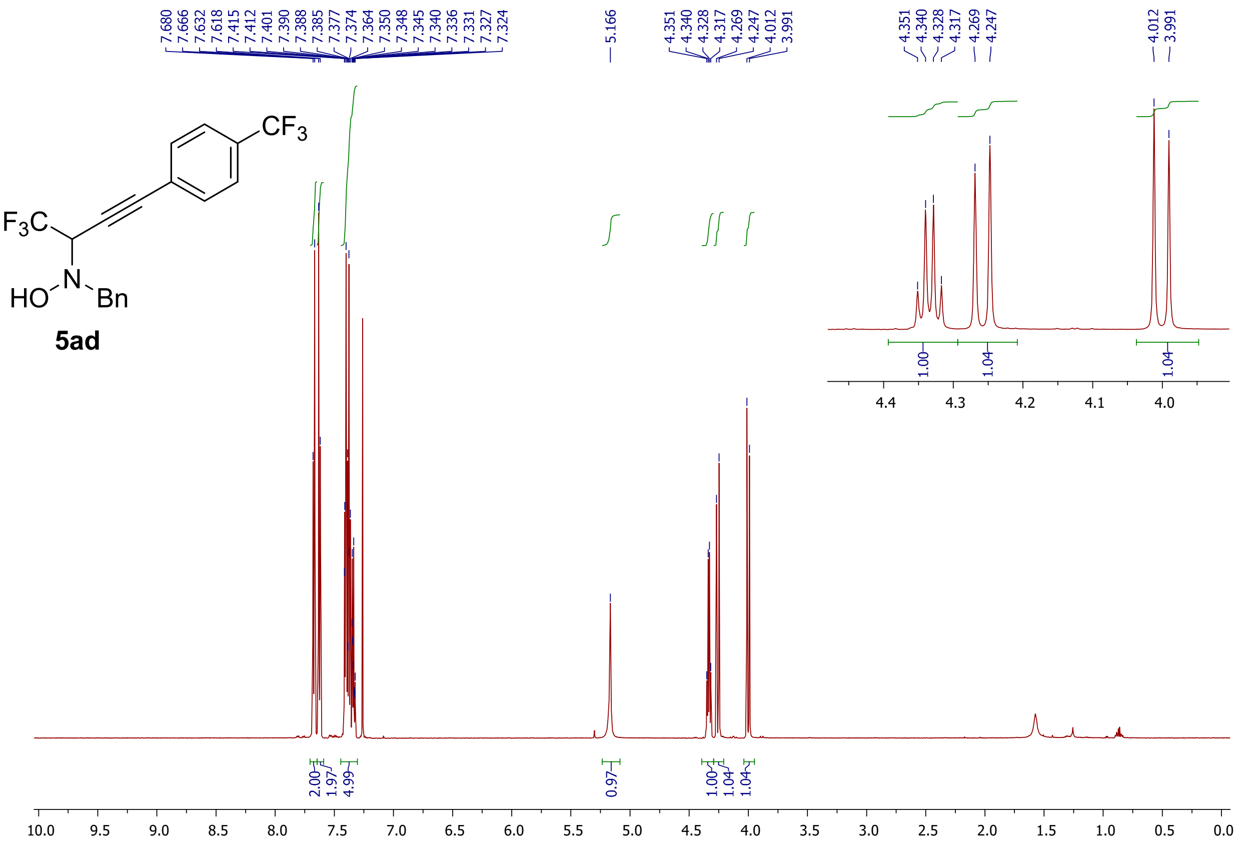

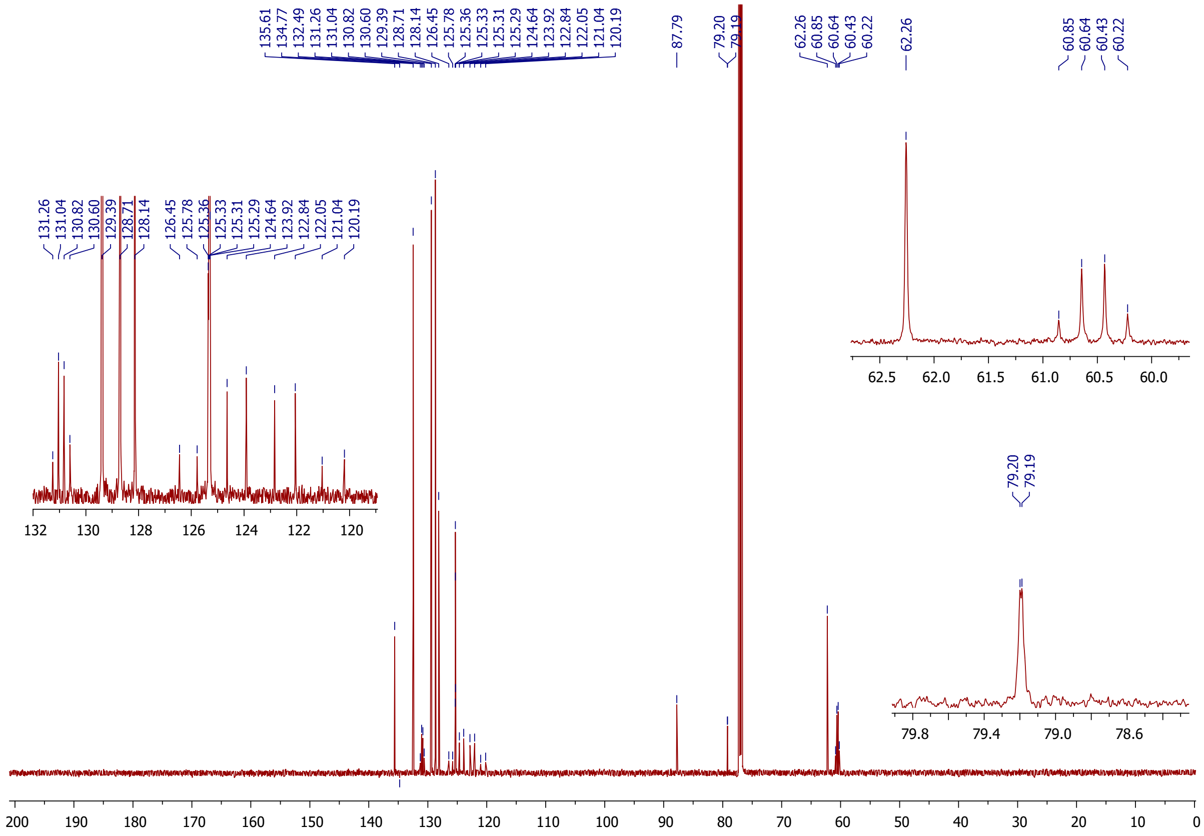

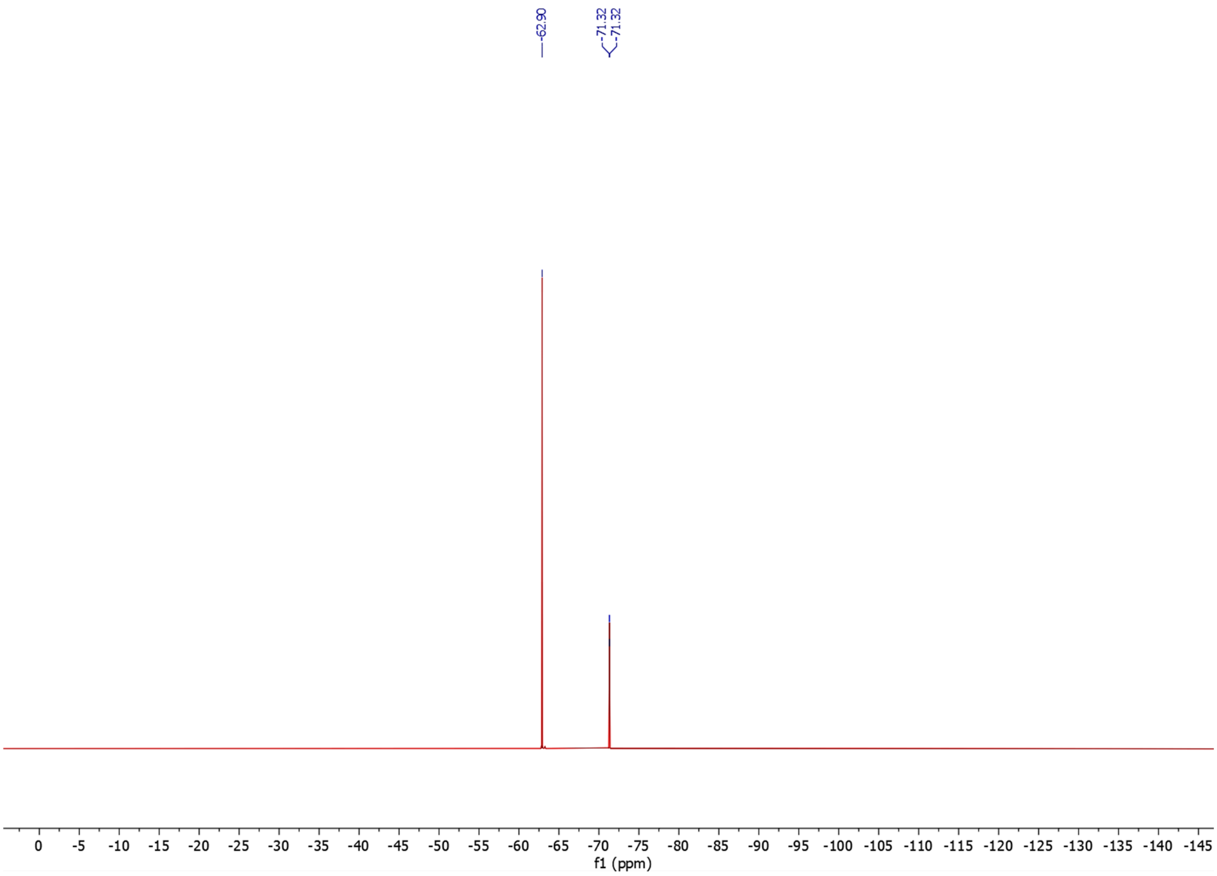
-62.90
-71.32
-71.32
0 -5 -10 -15 -20 -25 -30 -35 -40 -45 -50 -55 -60 -65 -70 -75 -80 -85 -90 -95 -100 -105 -110 -115 -120 -125 -130 -135 -140 -145
f1 (ppm)

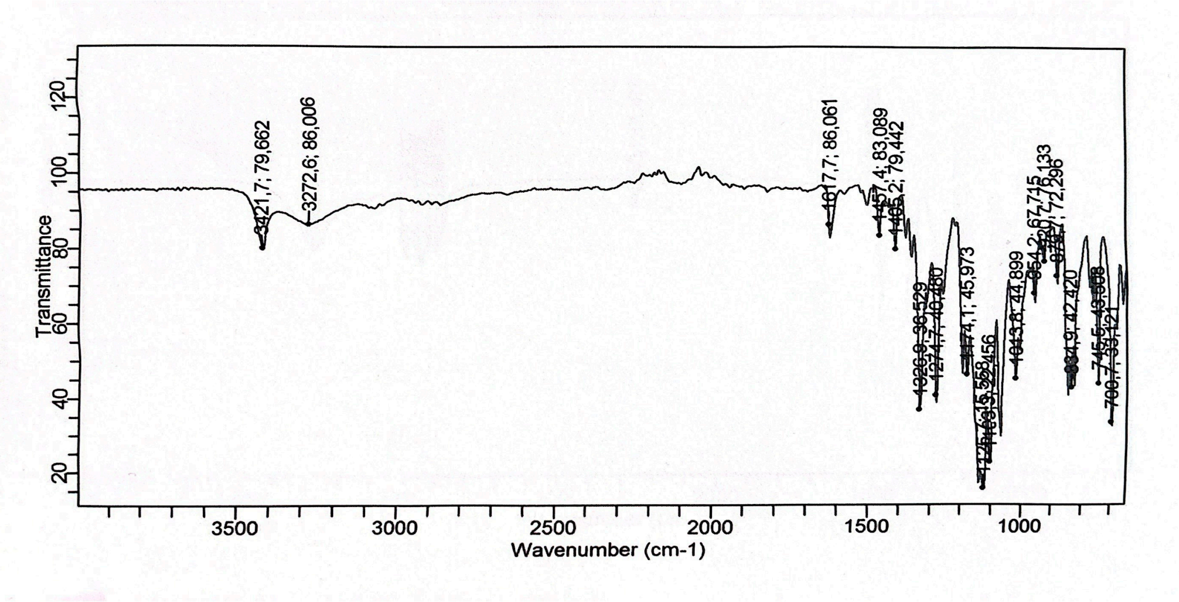
Transmittance
20
40
60
80
100
120
3421,7; 79,662
3272,6; 86,006
1617,7; 86,061
1274,1; 45,973
1013,8; 44,899
3500
3000
2500
2000
1500
1000
Wavenumber (cm-1)

2-Benzyl-5-(4′-trifluoromethylphenyl)-3-(trifluoromethyl)-2,3-dihydroizoxazole (**6ad**).

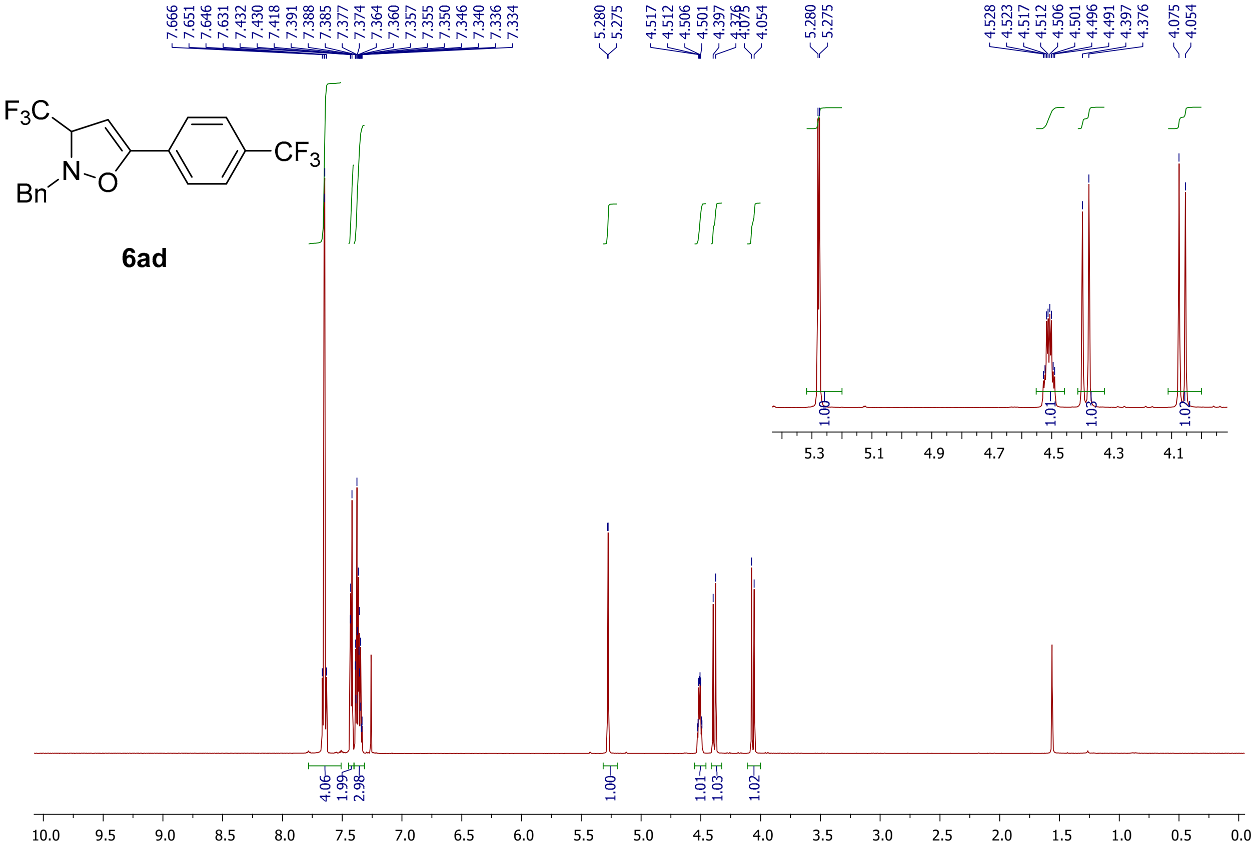

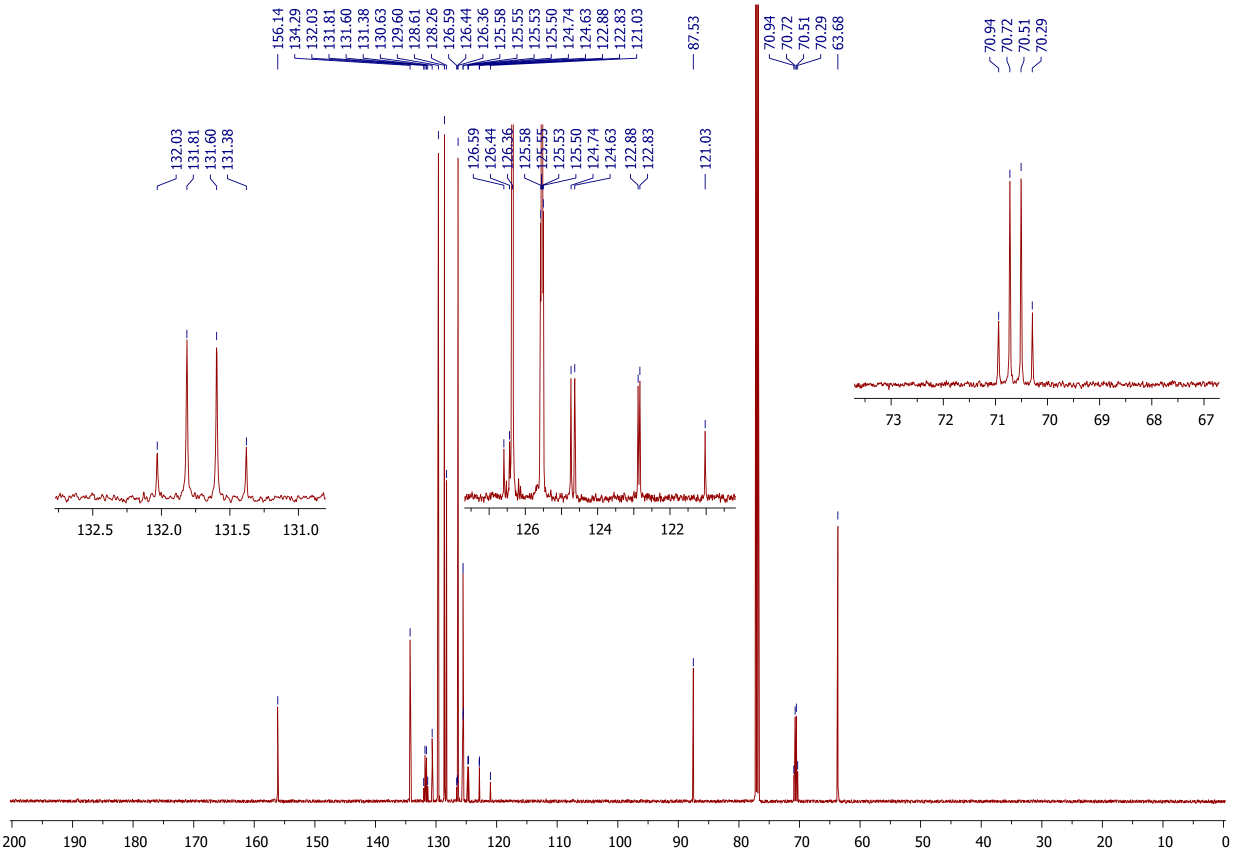

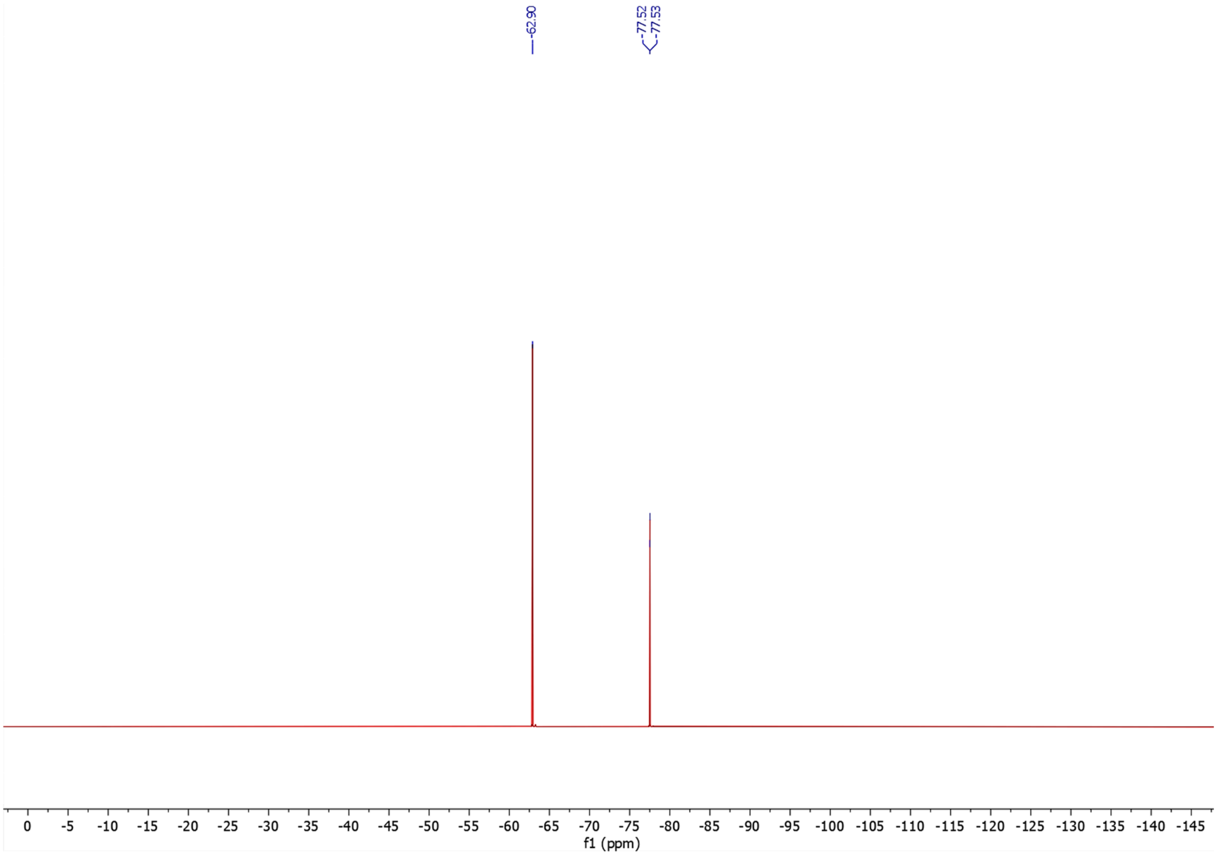

-62.90
-77.52
-77.53
0 -5 -10 -15 -20 -25 -30 -35 -40 -45 -50 -55 -60 -65 -70 -75 -80 -85 -90 -95 -100 -105 -110 -115 -120 -125 -130 -135 -140 -145
f1 (ppm)

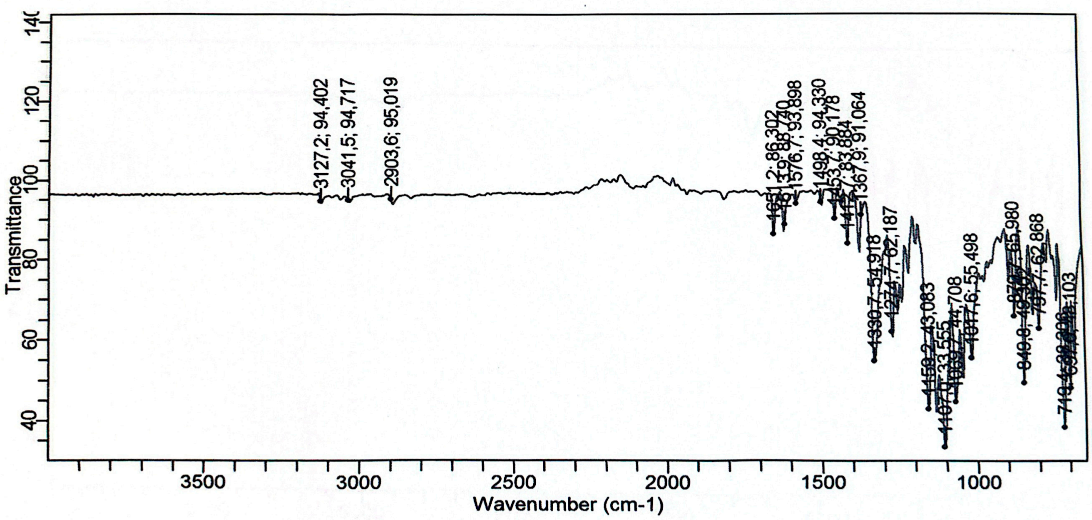

Transmittance
Wavenumber (cm-1)
3500 3000 2500 2000 1500 1000
40 60 80 100 120
3127,2; 94,402
3041,5; 94,717
2903,6; 95,019
1576,7; 93,898
1498,4; 94,330
1367,9; 91,064
1330,7; 54,918
1159,2; 43,083
1017,6; 55,498

N-*Benzyl*-N-*(1,1,1-trifluoro-5,5-dimethylheks-3-yn-2-yl)hydroxyloamine* (**5ae**).

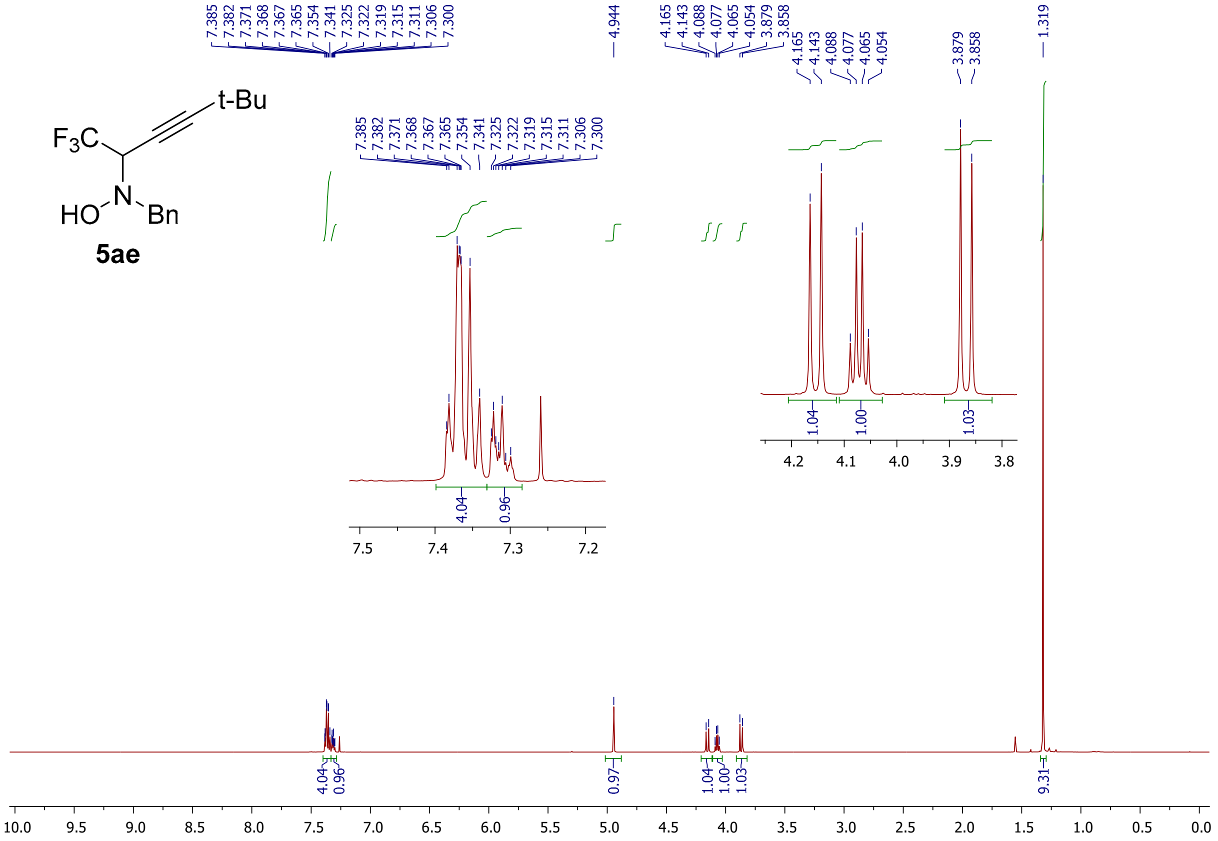

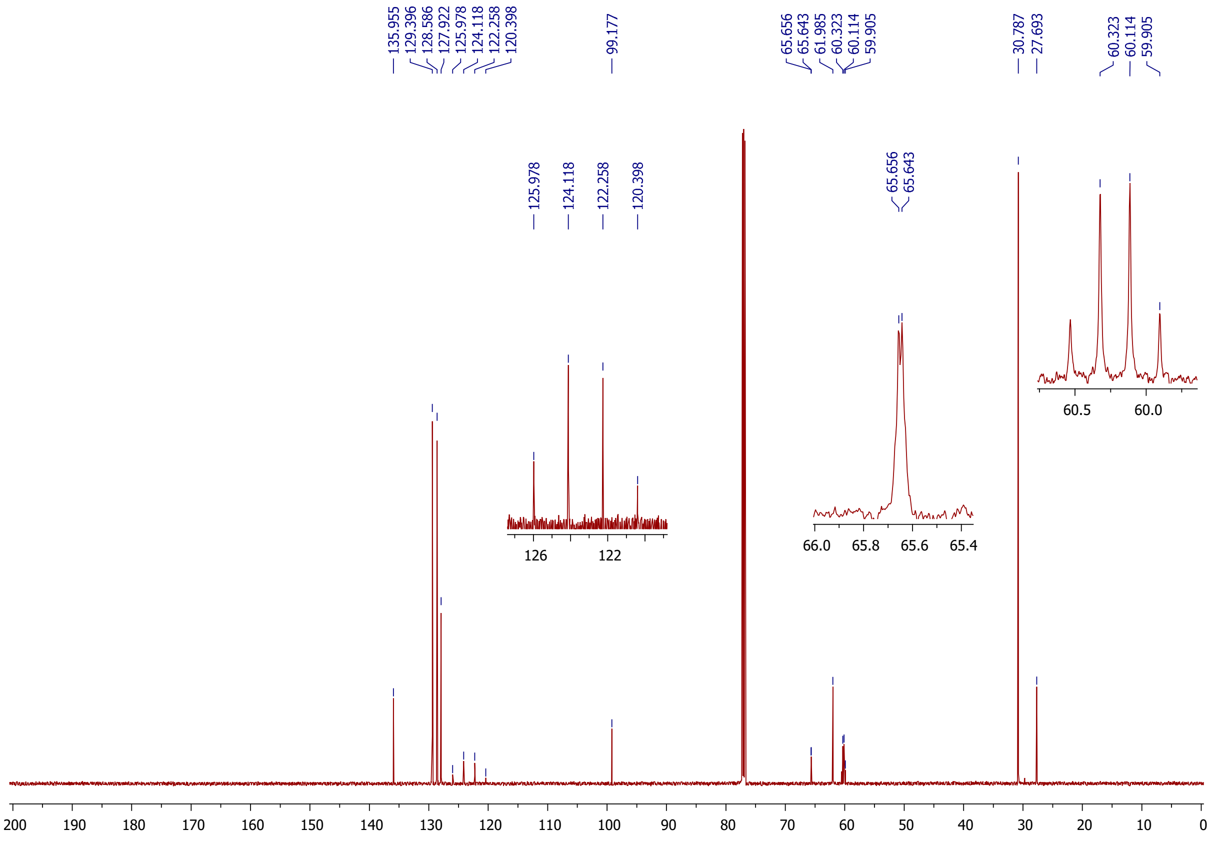

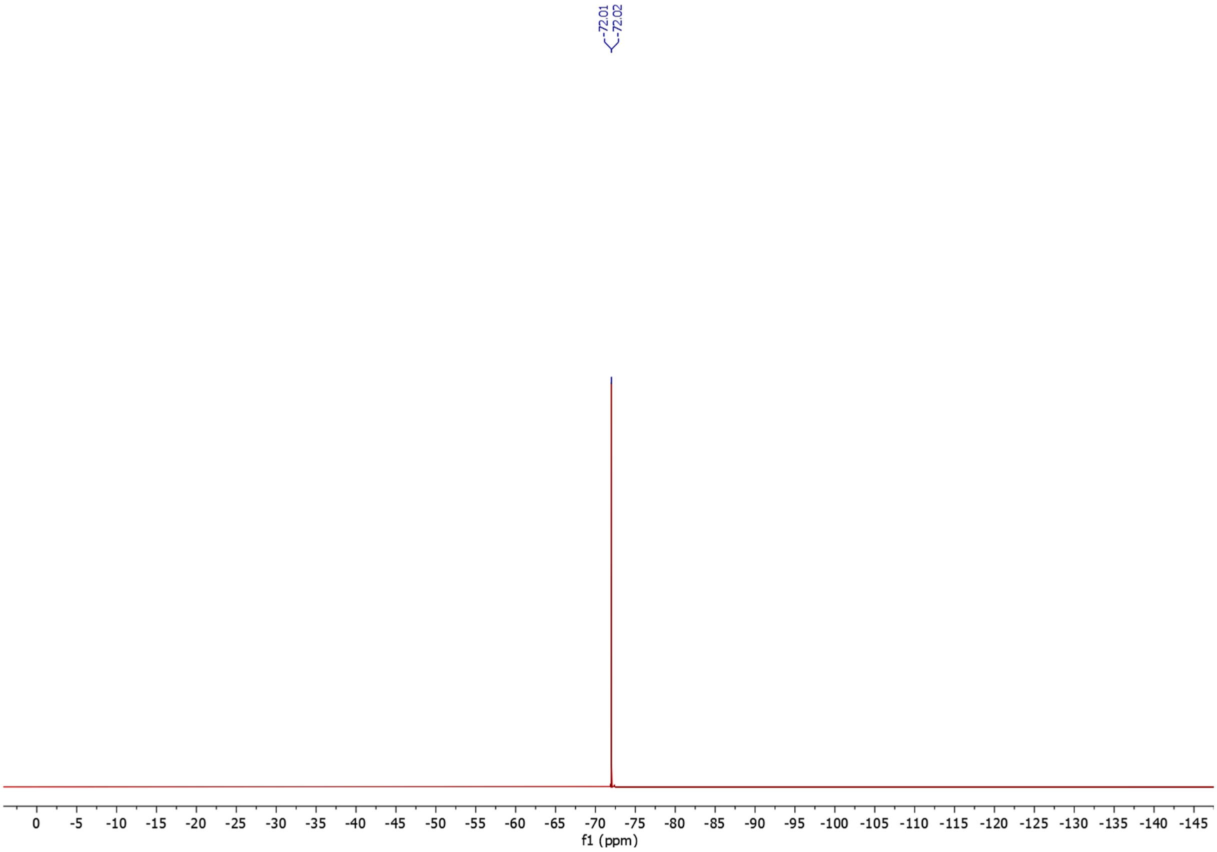
-72.01
-72.02
0 -5 -10 -15 -20 -25 -30 -35 -40 -45 -50 -55 -60 -65 -70 -75 -80 -85 -90 -95 -100 -105 -110 -115 -120 -125 -130 -135 -140 -145
f1 (ppm)

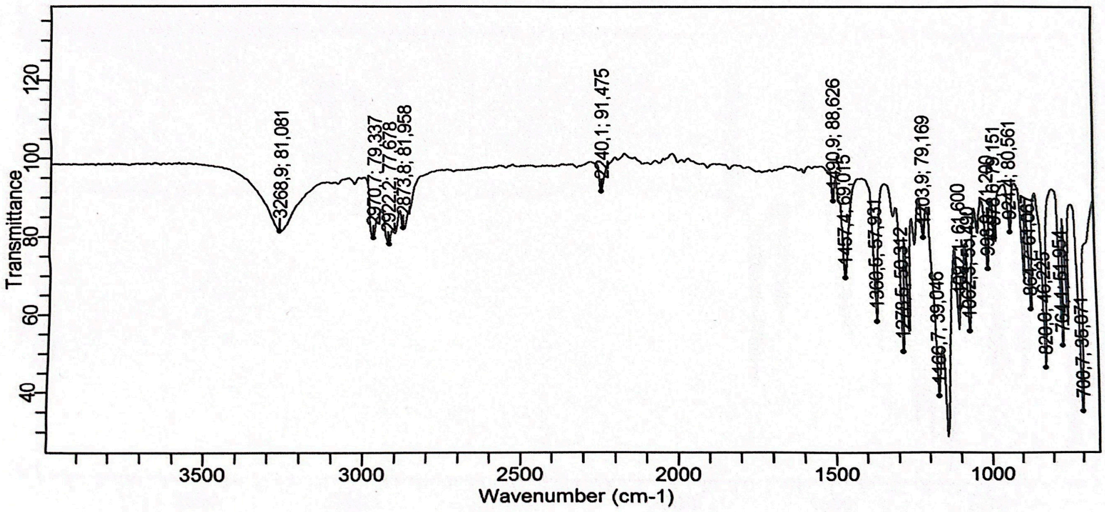
Transmittance
Wavenumber (cm-1)
3268,9; 81,081
2970,7; 79,337
2873,8; 81,958
2240,1; 91,475
1490,9; 88,626
1457,4; 69,015
1360,5; 57,931
1203,9; 79,169
1160,7; 39,046
1062,3; 55,990
824,1; 80,561
829,0; 46,225
708,7; 35,071
3500
3000
2500
2000
1500
1000
40
60
80
100
120

Methyl 2-Benzyl-3-(trifluoromethyl)-4-isoxazoline-5-carboxylate (**6af**).

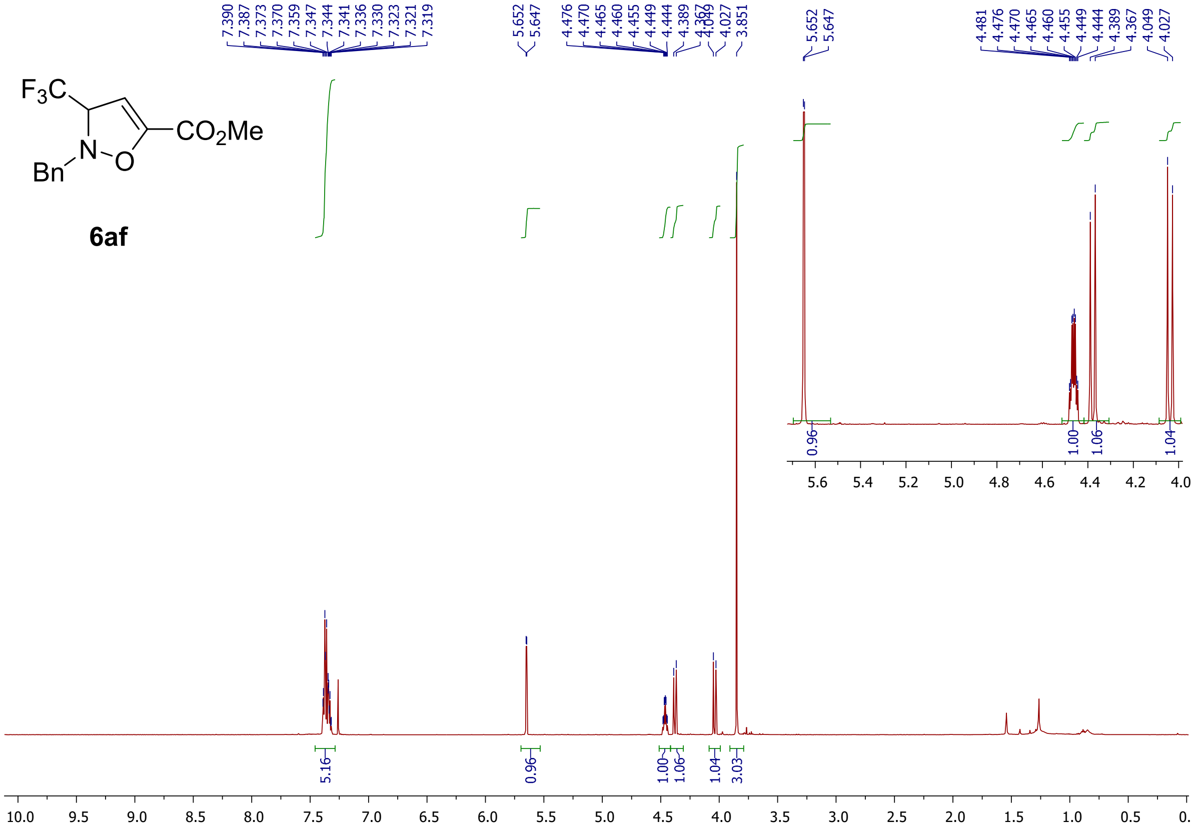

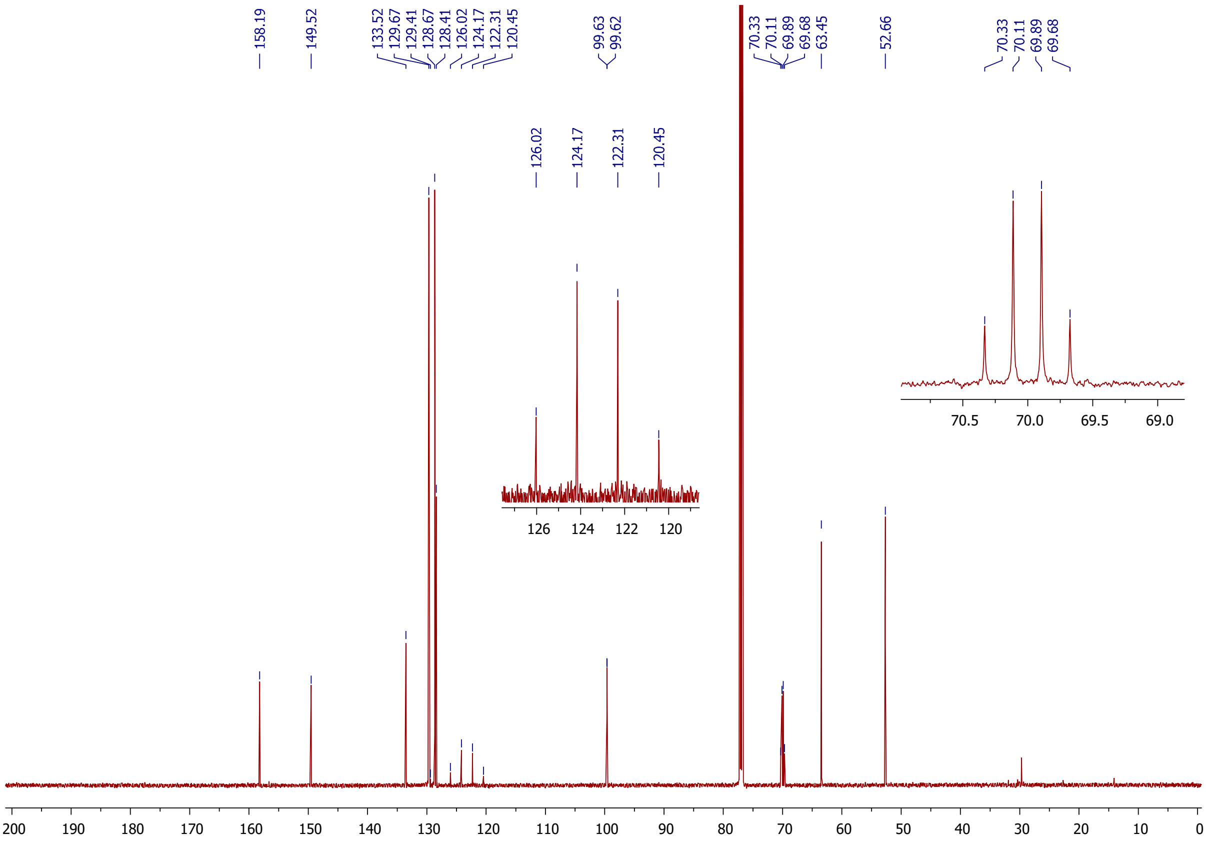

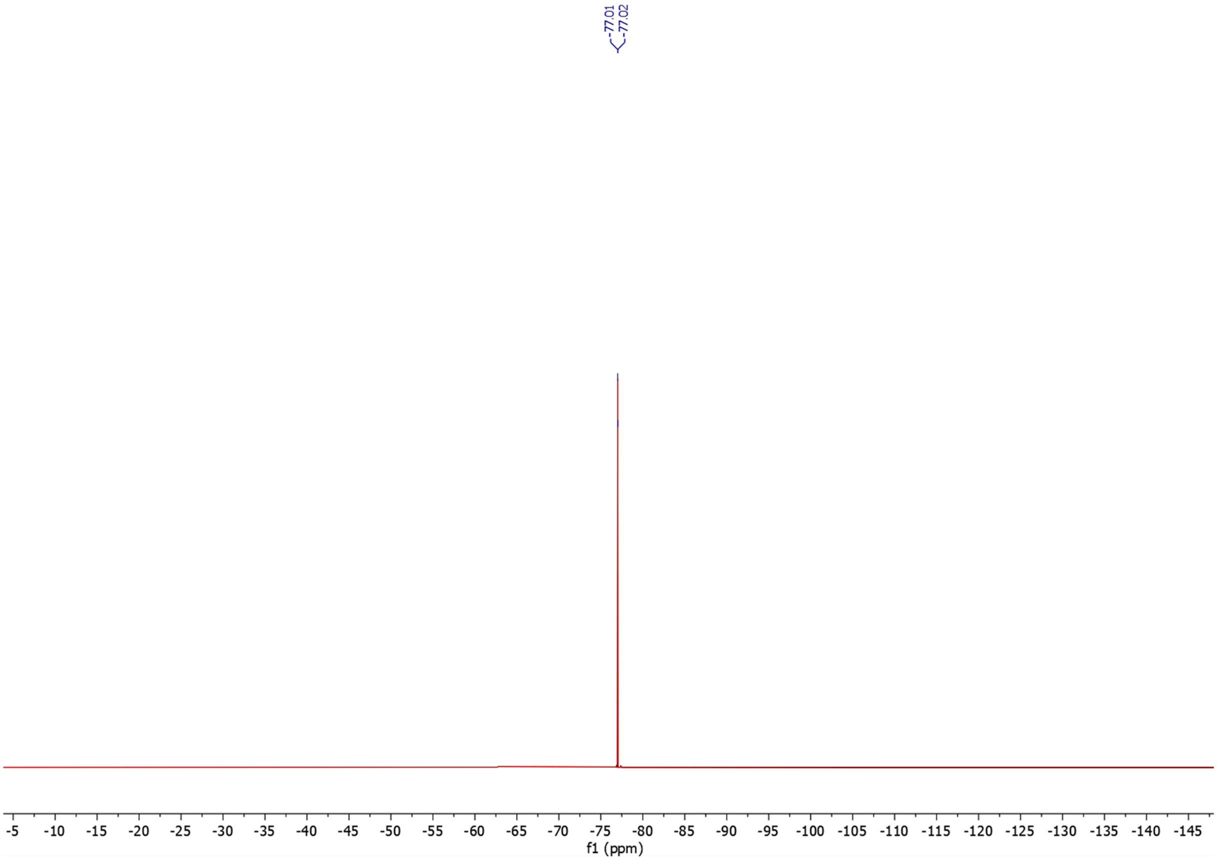
-77.01
-77.02
-5 -10 -15 -20 -25 -30 -35 -40 -45 -50 -55 -60 -65 -70 -75 -80 -85 -90 -95 -100 -105 -110 -115 -120 -125 -130 -135 -140 -145
f1 (ppm)

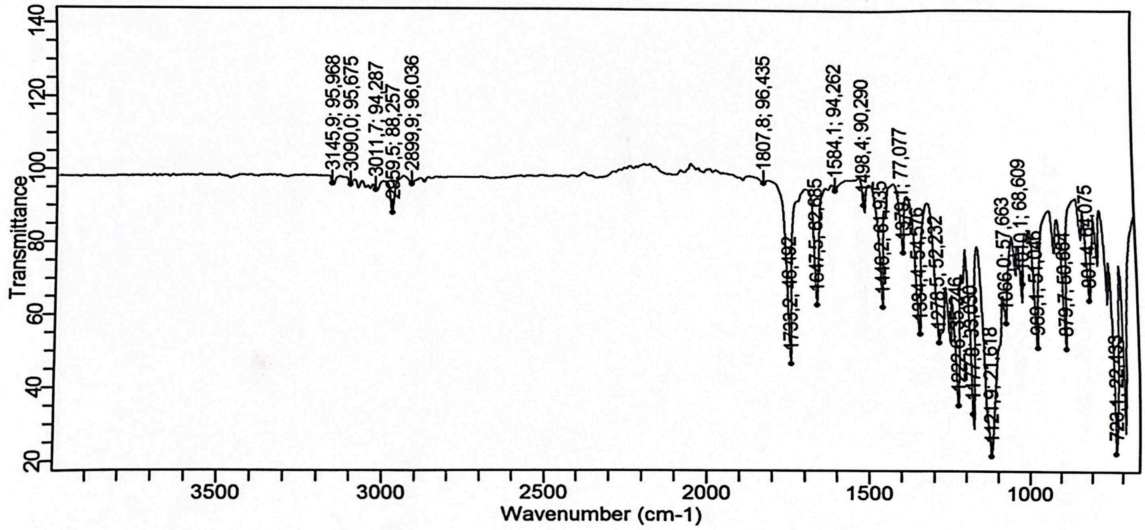
Transmittance
Wavenumber (cm-1)
3500
3000
2500
2000
1500
1000
3145,9; 95,968
3090,0; 95,675
3011,7; 94,287
2959,5; 88,257
2899,9; 96,036
1807,8; 96,435
1584,1; 94,262
1498,4; 90,290
1733,2; 46,482
1647,5; 62,685

N-*Benzyl*-N-*(5,5-diethoxy-1,1,1-trifluoropent-3-yn-2-yl)hydroxylamine* (**5ag**).

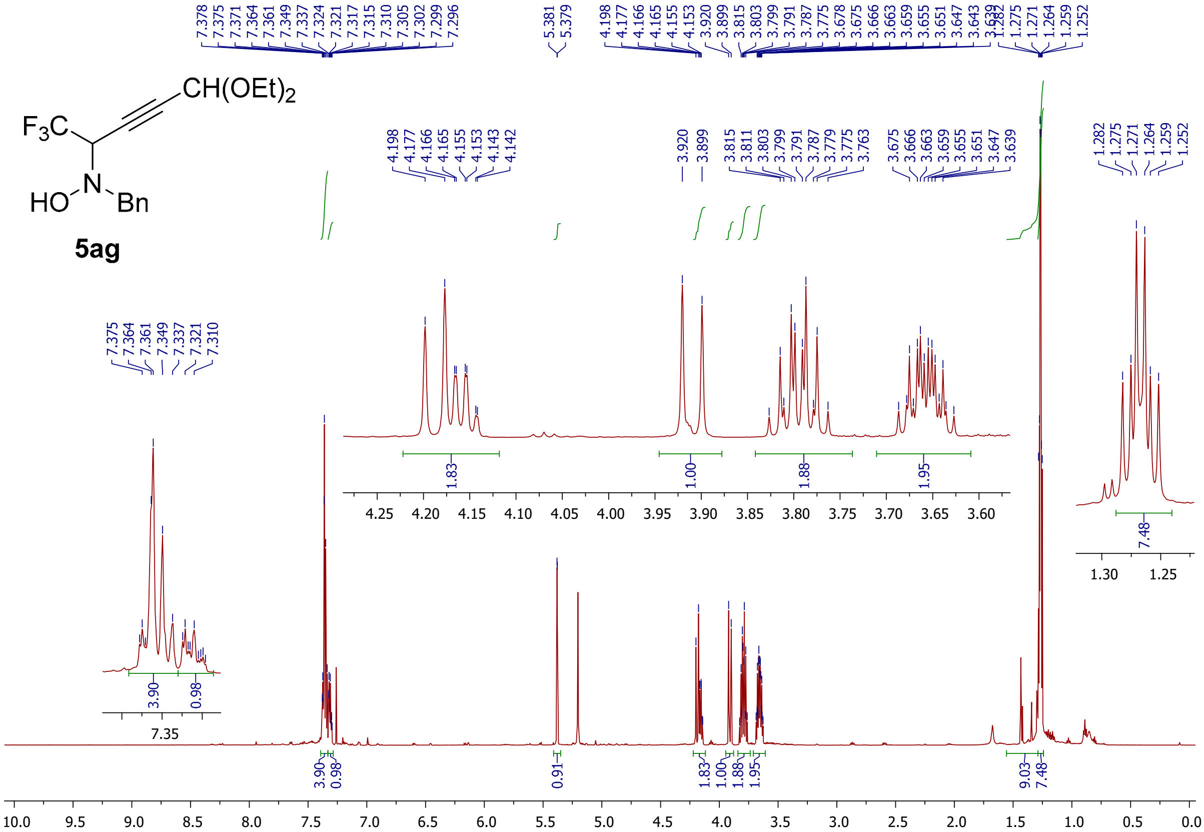

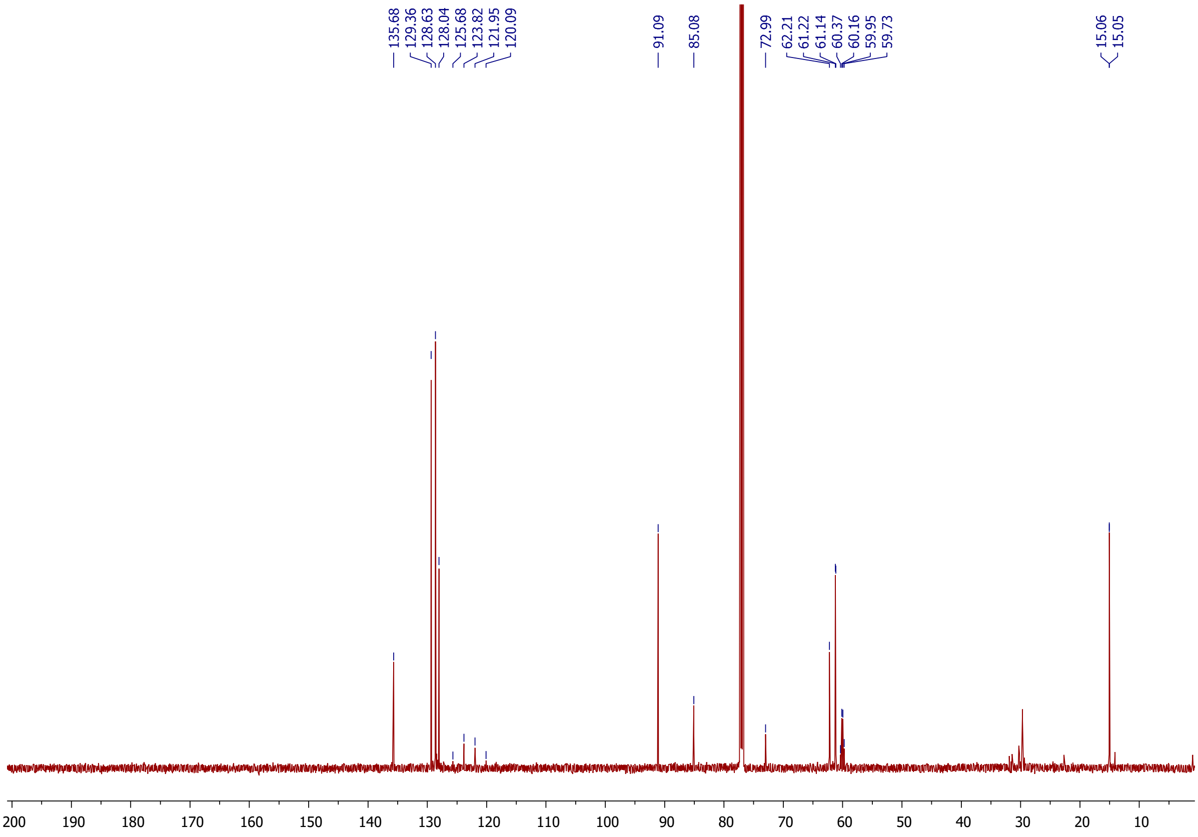

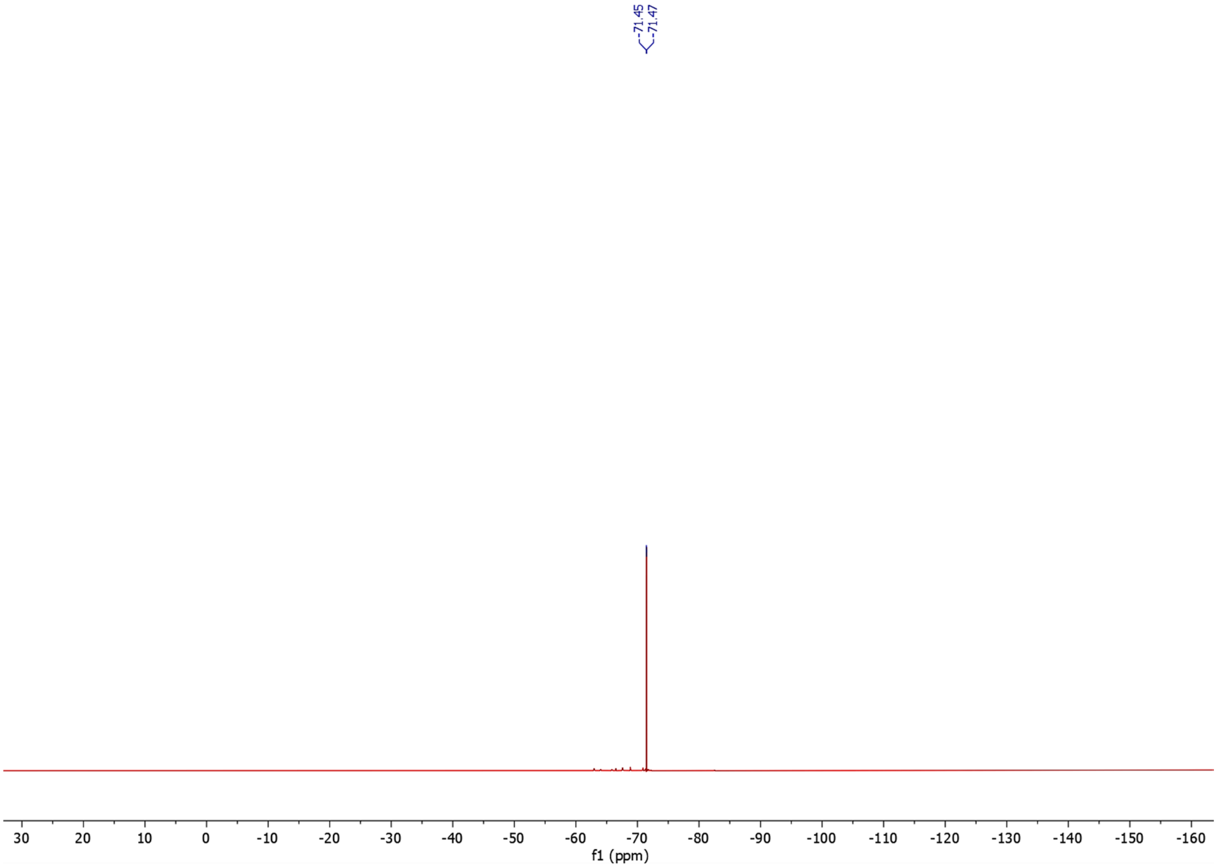
-71.45
-71.47
f1 (ppm)

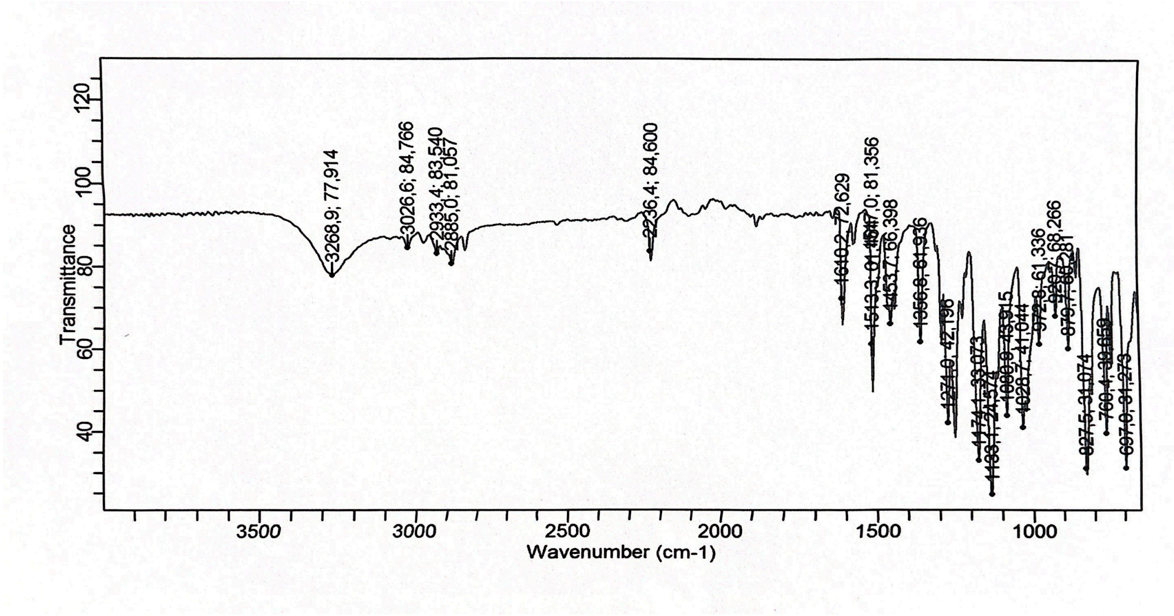
Transmittance
Wavenumber (cm-1)
3268,9; 77,914
3026,6; 84,766
2933,4; 83,540
2885,0; 81,057
2236,4; 84,600
1453,7; 66,398
1274,0; 42,196
972,8; 61,336
827,5; 31,074
697,0; 31,273

2. HPLC analysis

***N*-Benzyl-*N*-(1,1,1-trifluoro-4-phenylbut-3-yn-2-yl)hydroxylamine** **(5aa)**: *ee* determination condition: Chiralcel AD-H, Hexanes : ^{i}PrOH = 90:10, flow = 1.0 mL/min, retention time: 6.59 (minor), 7.39 (major) min.

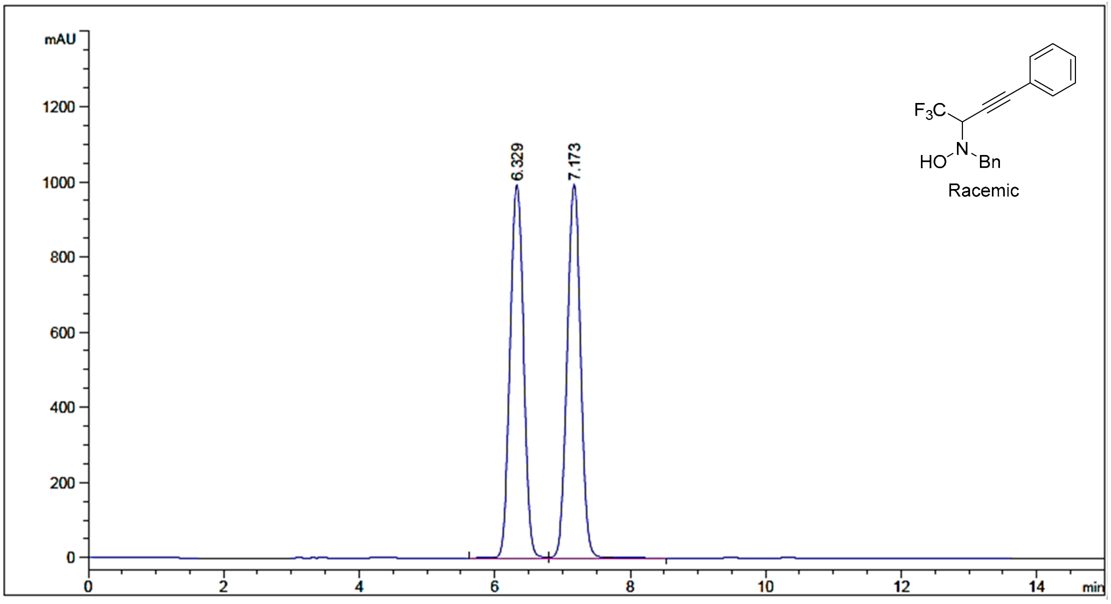


```
Signal 1: DAD1 A, Sig=250,4 Ref=off

Peak RetTime Type  Width     Area      Height     Area
  #   [min]        [min]   [mAU*s]     [mAU]       %
----|-------|----|-------|----------|----------|--------|
   1   6.329 BV    0.2196 1.37295e4  991.05255  49.9023
   2   7.173 VB    0.2201 1.37833e4  992.29047  50.0977
```

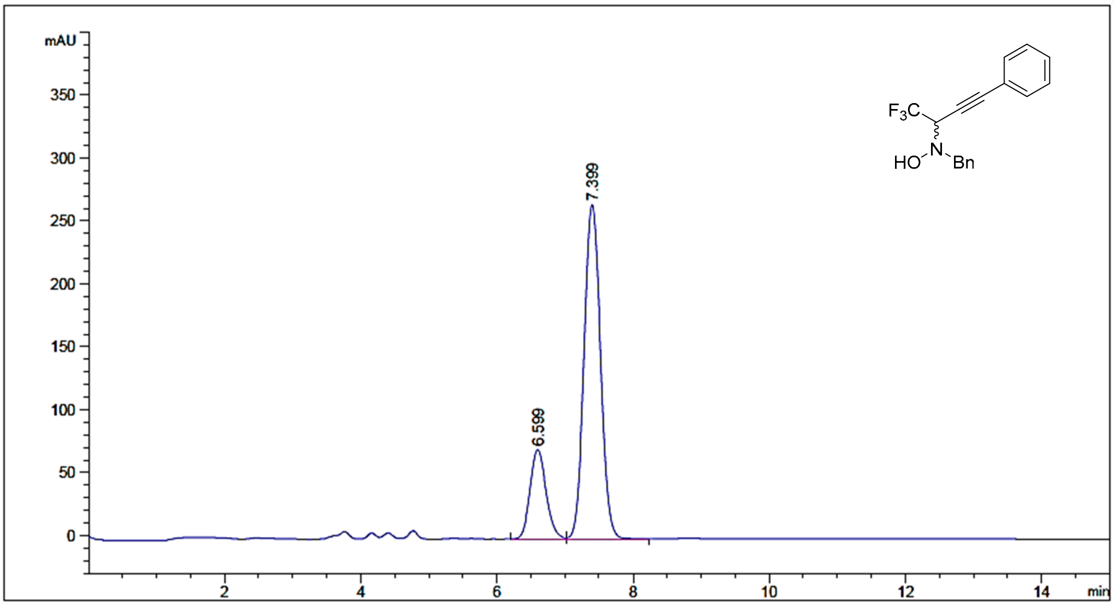


```
Signal 1: DAD1 A, Sig=250,4 Ref=off

Peak RetTime Type  Width     Area      Height     Area
  #   [min]        [min]   [mAU*s]     [mAU]       %
----|-------|----|-------|----------|----------|--------|
   1   6.599 BV    0.2533 1162.05225   70.86443  20.7633
   2   7.399 VB    0.2650 4434.61328  265.38214  79.2367
```

***N*-Benzyl-N-[1,1,1-trifluoro-4-(4′-methoxyphenyl)but-3-yn-2-yl]hydroxylamine** (**5ab**): *ee* determination condition: Chiralcel AD-H, Hexanes : ^{i}PrOH = 95:5, flow = 1.0 mL/min, retention time: 18.54 (minor), 20.29 (major) min.

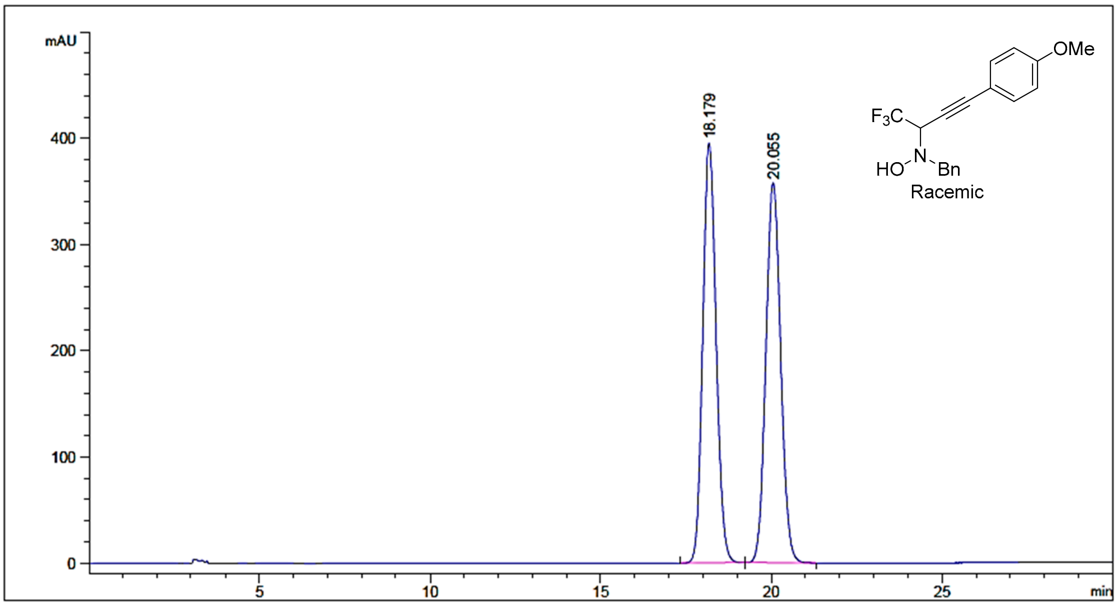

Signal 1: DAD1 A, Sig=250,4 Ref=off

Peak #	RetTime [min]	Type	Width [min]	Area [mAU*s]	Height [mAU]	Area %
1	18.179	BB	0.4225	1.07312e4	395.52380	49.8275
2	20.055	BB	0.4701	1.08055e4	357.99228	50.1725

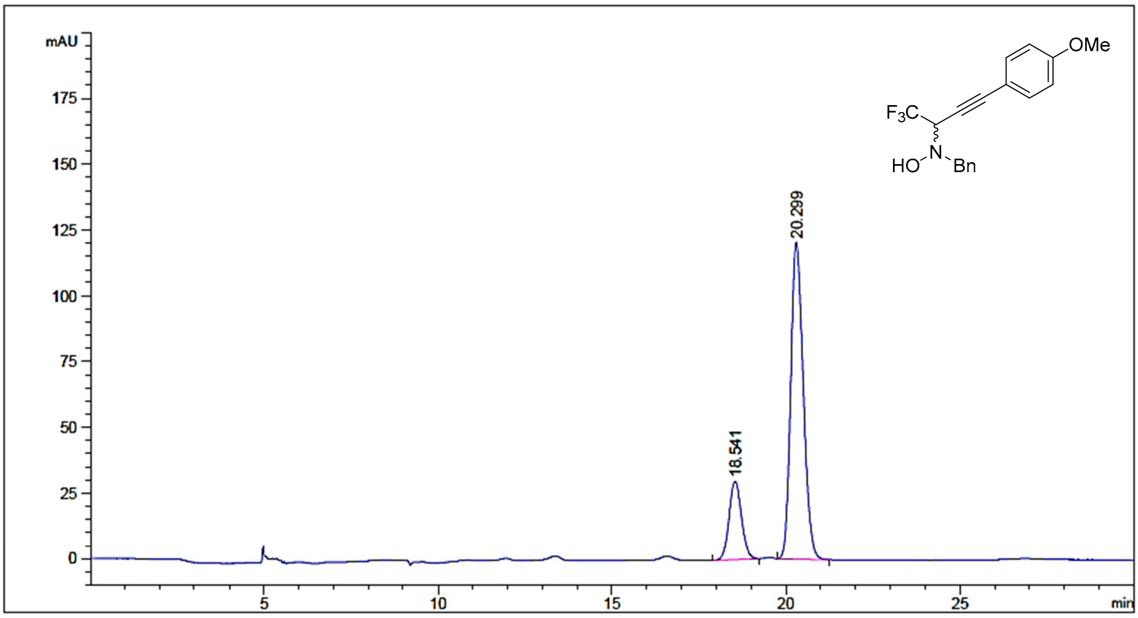

Signal 1: DAD1 A, Sig=250,4 Ref=off

Peak #	RetTime [min]	Type	Width [min]	Area [mAU*s]	Height [mAU]	Area %
1	18.541	BB	0.3899	743.15570	29.90510	19.7970
2	20.299	BB	0.3878	3010.71802	120.36557	80.2030

***N*-Benzyl-*N*-[1,1,1-trifluoro-4-(4'-chlorophenyl)but-3-yn-2-yl]hydroxylamine** (**5ac**): *ee* determination condition: Chiralcel AD-H, Hexanes : ^{i}PrOH = 95:5, flow = 1.0 mL/min, retention time: 12.65 (minor), 17.19 (major) min.

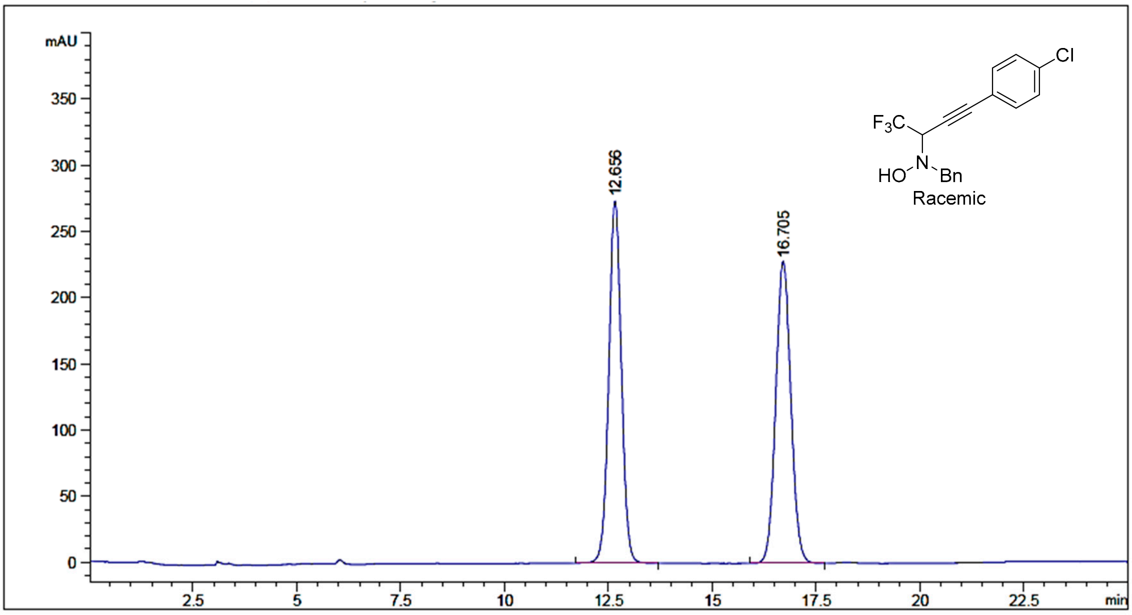

Signal 1: DAD1 A, Sig=250,4 Ref=off

Peak #	RetTime [min]	Type	Width [min]	Area [mAU*s]	Height [mAU]	Area %
1	12.656	BB	0.3238	5768.21045	273.76245	49.8957
2	16.705	BB	0.3946	5792.32861	227.85965	50.1043

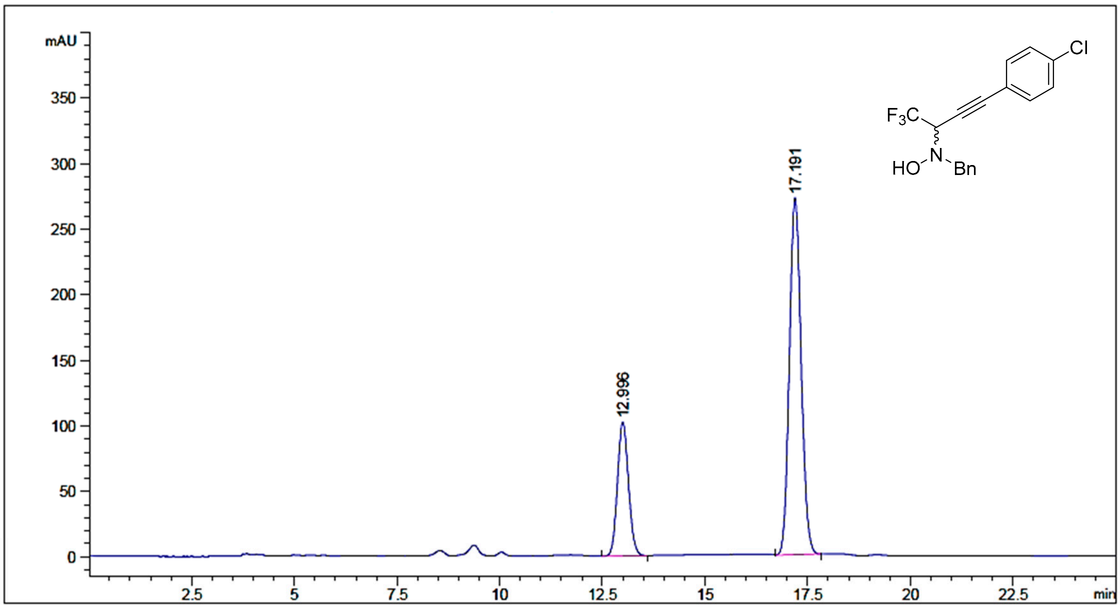

Signal 1: DAD1 A, Sig=250,4 Ref=off

Peak #	RetTime [min]	Type	Width [min]	Area [mAU*s]	Height [mAU]	Area %
1	12.996	BB	0.2924	1916.54651	102.36933	26.1098
2	17.191	BB	0.3082	5423.78857	272.46326	73.8902

***N*-Benzyl-*N*-[1,1,1-trifluoro-4-(4'-trifluoromethylphenyl)but-3-yn-2-yl]hydroxylamine** (**5ad**): *ee* determination condition: Chiralcel AD-H, Hexanes : ^{i}PrOH = 95:5, flow = 1.0 mL/min, retention time: 13.38 (minor), 17.84 (major) min.

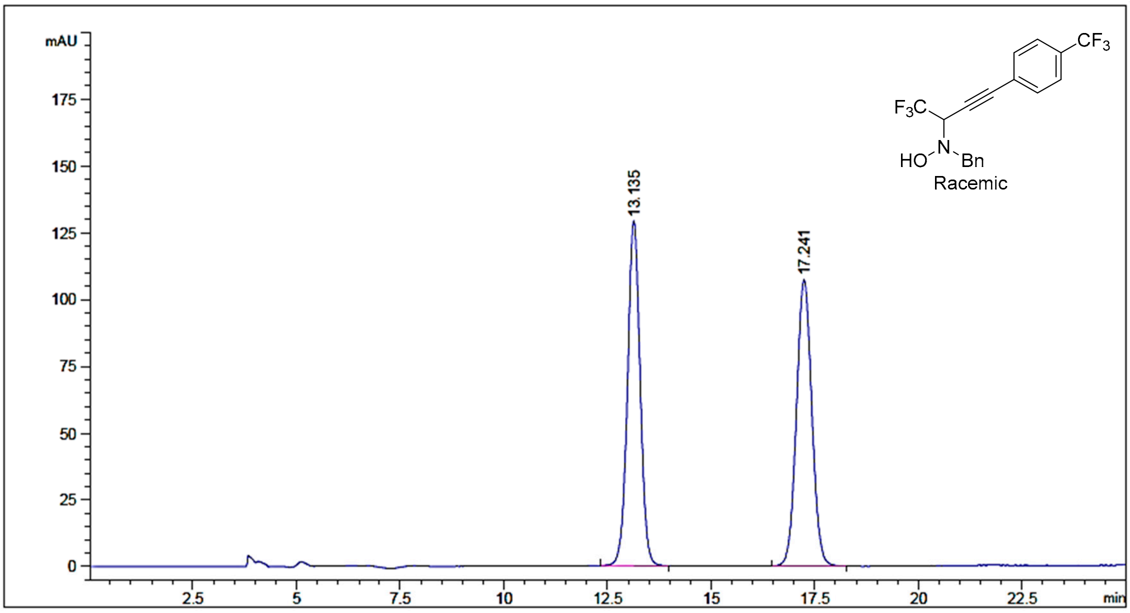

Signal 1: DAD1 A, Sig=250,4 Ref=off

Peak #	RetTime [min]	Type	Width [min]	Area [mAU*s]	Height [mAU]	Area %
1	13.135	BB	0.3276	2738.68286	129.02455	49.7710
2	17.241	BB	0.4028	2763.88428	107.19583	50.2290

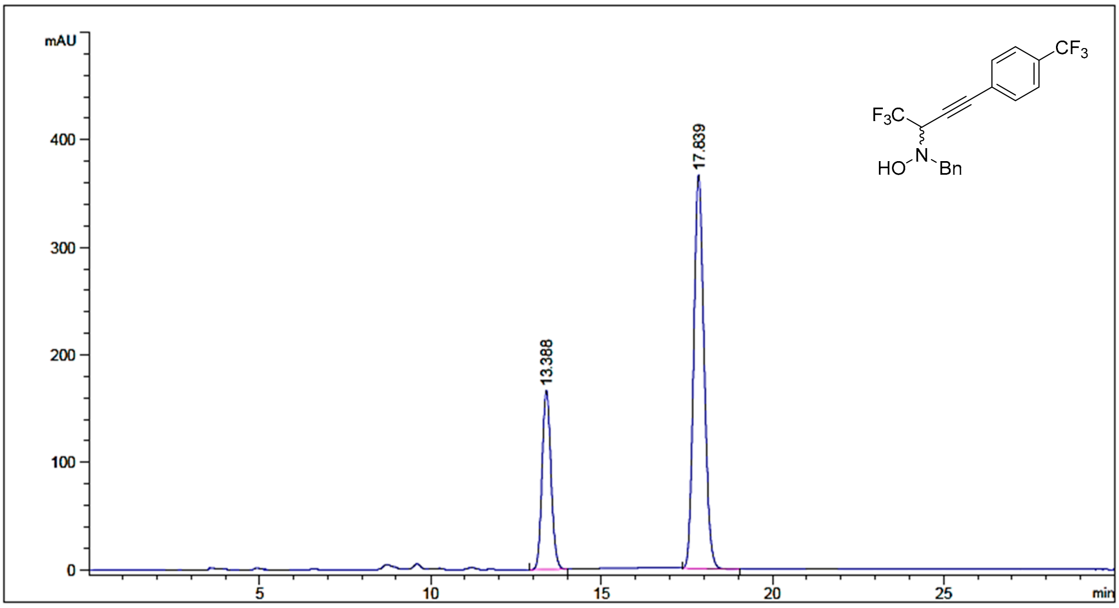

Signal 1: DAD1 A, Sig=250,4 Ref=off

Peak #	RetTime [min]	Type	Width [min]	Area [mAU*s]	Height [mAU]	Area %
1	13.388	BB	0.2812	3021.96289	166.94653	28.0933
2	17.839	BB	0.3283	7734.92676	366.36826	71.9067

***N*-Benzyl-*N*-(1,1,1-trifluoro-5,5-dimethylheks-3-yn-2-yl)hydroxyloamine** (**5ae**): *ee* determination condition: Chiralcel AD, Hexanes : ^{i}PrOH = 98:2, flow = 0.5 mL/min, retention time: 12.55 (minor), 14.23 (major) min.

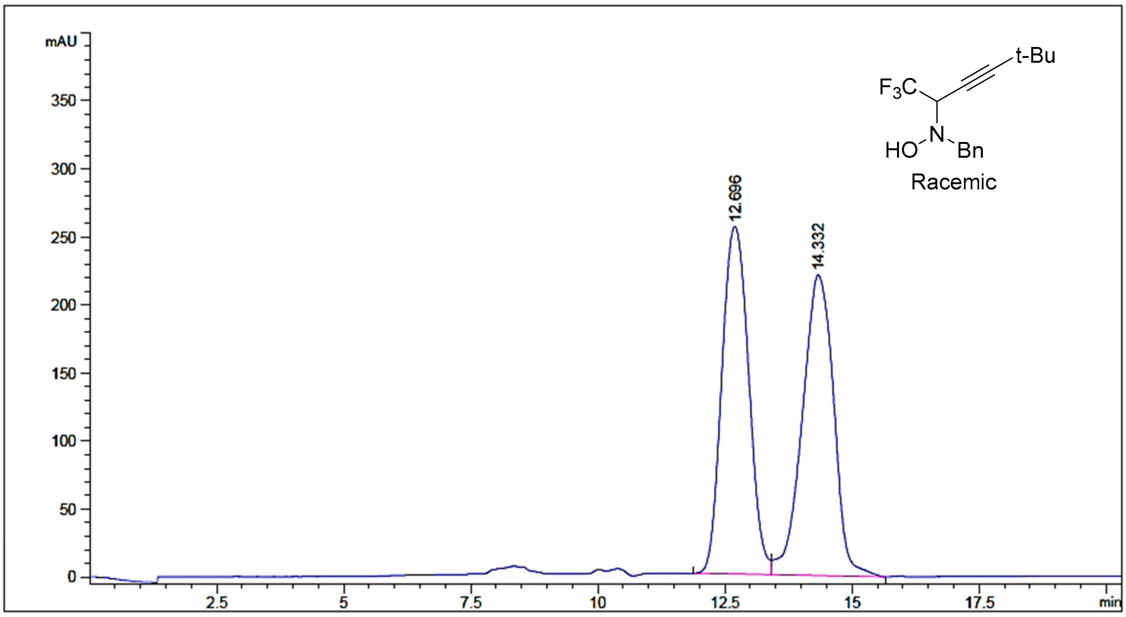

Signal 1: DAD1 A, Sig=250,4 Ref=off

Peak #	RetTime [min]	Type	Width [min]	Area [mAU*s]	Height [mAU]	Area %
1	12.696	BV	0.5892	9351.90723	255.38837	49.6450
2	14.332	VB	0.6726	9485.65625	221.06870	50.3550

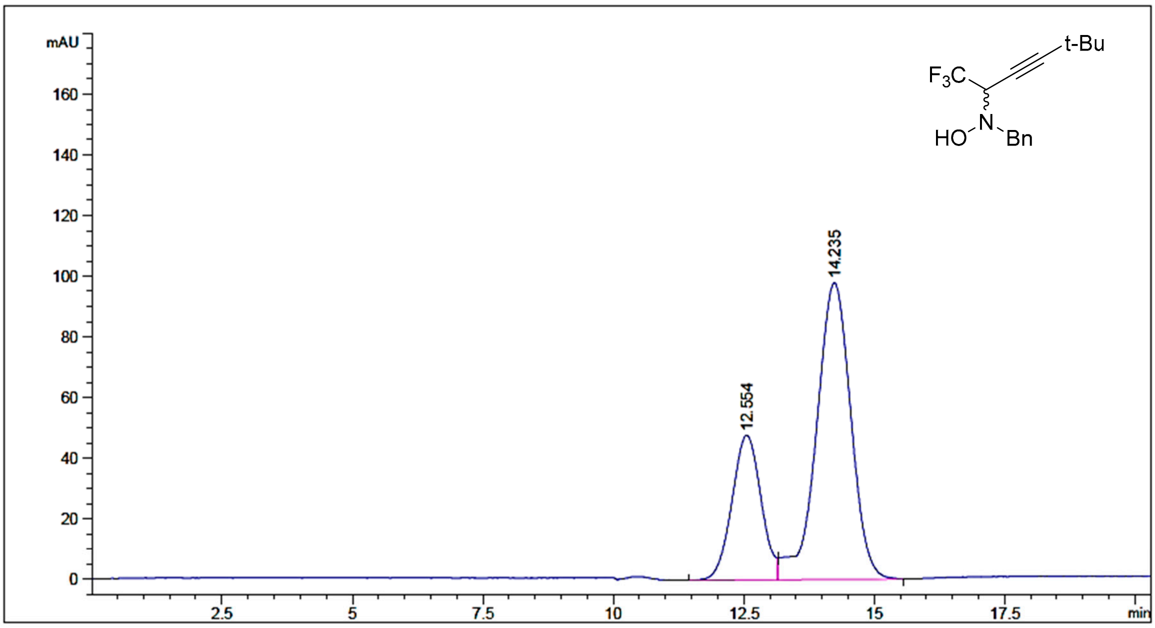

Signal 1: DAD1 A, Sig=250,4 Ref=off

Peak #	RetTime [min]	Type	Width [min]	Area [mAU*s]	Height [mAU]	Area %
1	12.554	BV	0.5949	1856.65527	47.82417	29.2484
2	14.235	VB	0.6992	4491.22363	97.90887	70.7516

***N*-Benzyl-*N*-(1,1-difluoro-4-phenylbut-3-yn-2-yl)hydroxylamine** (**5ba**): *ee* determination condition: Chiralcel AD-H, Hexanes : ^{i}PrOH = 90:10, flow = 0.7 mL/min, retention time: 12.55 (minor), 14.23 (major) min.

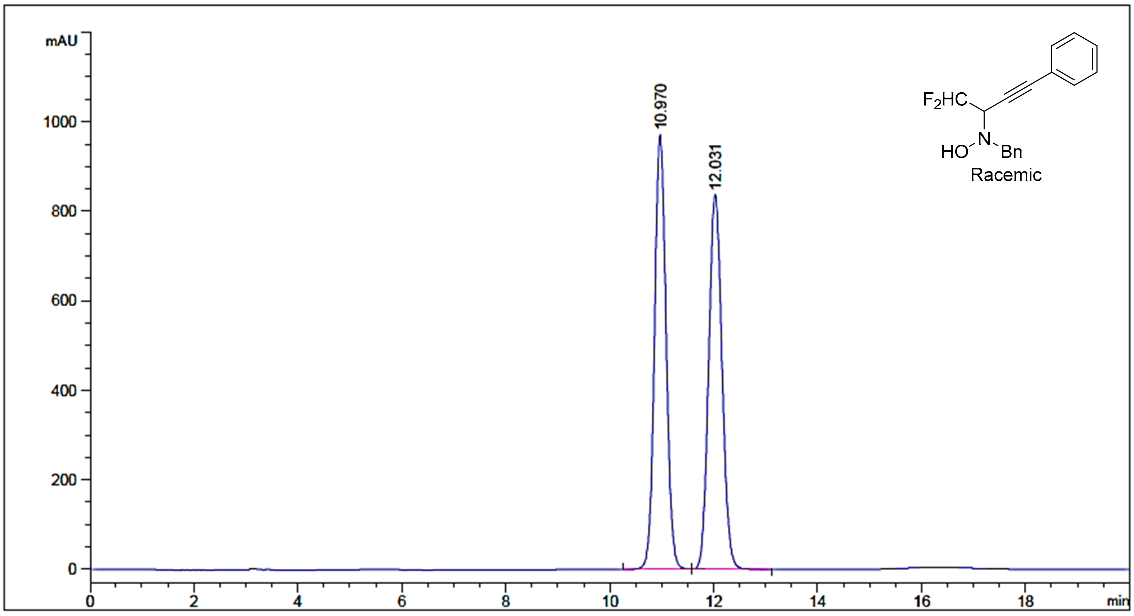

Signal 1: DAD1 A, Sig=250,4 Ref=off

Peak #	RetTime [min]	Type	Width [min]	Area [mAU*s]	Height [mAU]	Area %
1	10.970	BB	0.2397	1.49445e4	970.27203	50.0511
2	12.031	BB	0.2799	1.49140e4	837.05756	49.9489

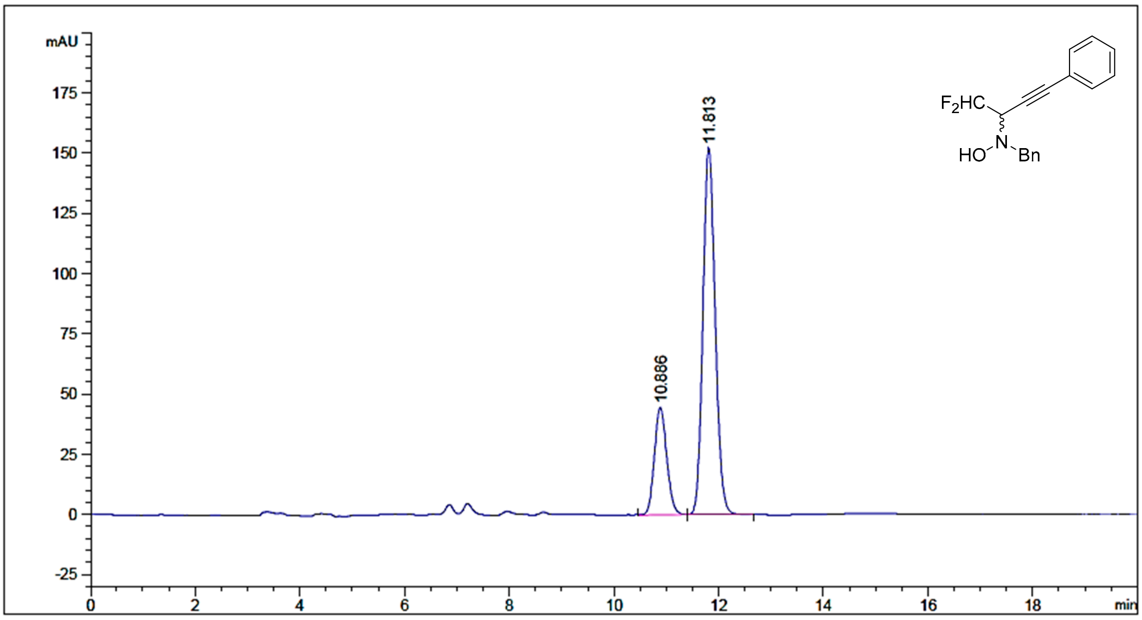

Signal 1: DAD1 A, Sig=250,4 Ref=off

Peak #	RetTime [min]	Type	Width [min]	Area [mAU*s]	Height [mAU]	Area %
1	10.886	BB	0.2552	729.34686	44.49515	22.7850
2	11.813	BB	0.2517	2471.64868	151.97061	77.2150

2-benzyl-5-phenyl-3-(trifluoromethyl)-2,3-dihydroisoxazole **(6aa):** *ee* determination condition: Chiralcel AD, Hexanes : ^{i}PrOH = 90:10, flow = 0.5 mL/min, retention time: 9.36, 10.14 min.

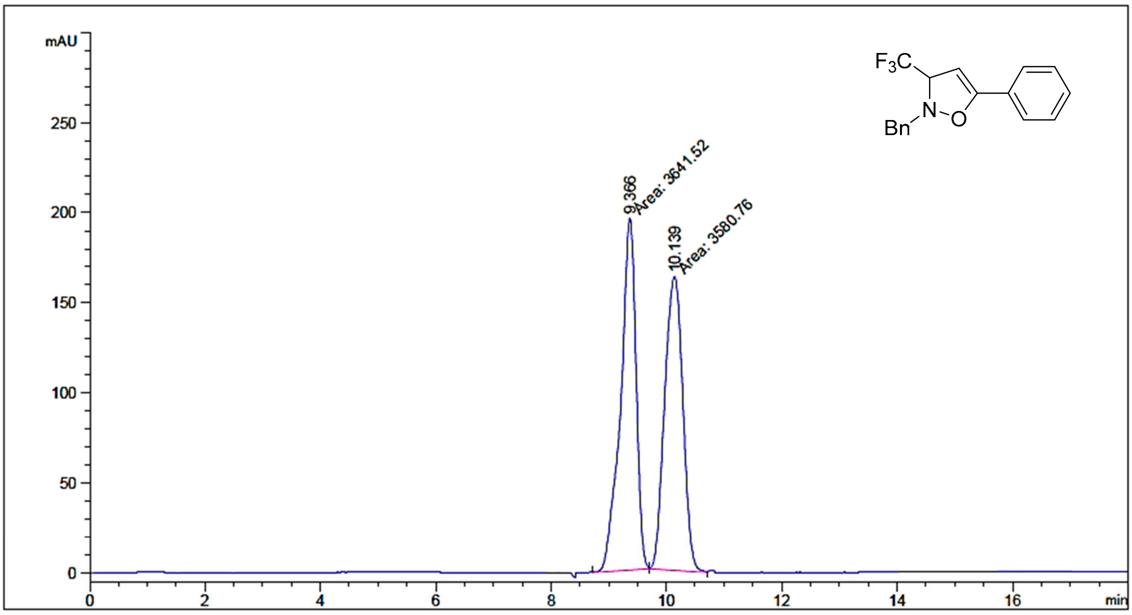

Signal 1: DAD1 A, Sig=250,4 Ref=off

Peak #	RetTime [min]	Type	Width [min]	Area [mAU*s]	Height [mAU]	Area %
1	9.366	MM	0.3104	3641.51880	195.54721	50.4206
2	10.139	MM	0.3657	3580.76050	163.19232	49.5794

2-Benzyl-5-(4′-methoxyphenyl)-3-(trifluoromethyl)-2,3-dihydroizoxazole (**6ab**): *ee* determination condition: Chiralcel AD, Hexanes : ^{i}PrOH = 95:5, flow = 0.5 mL/min, retention time: 13.23, 14.15 min.

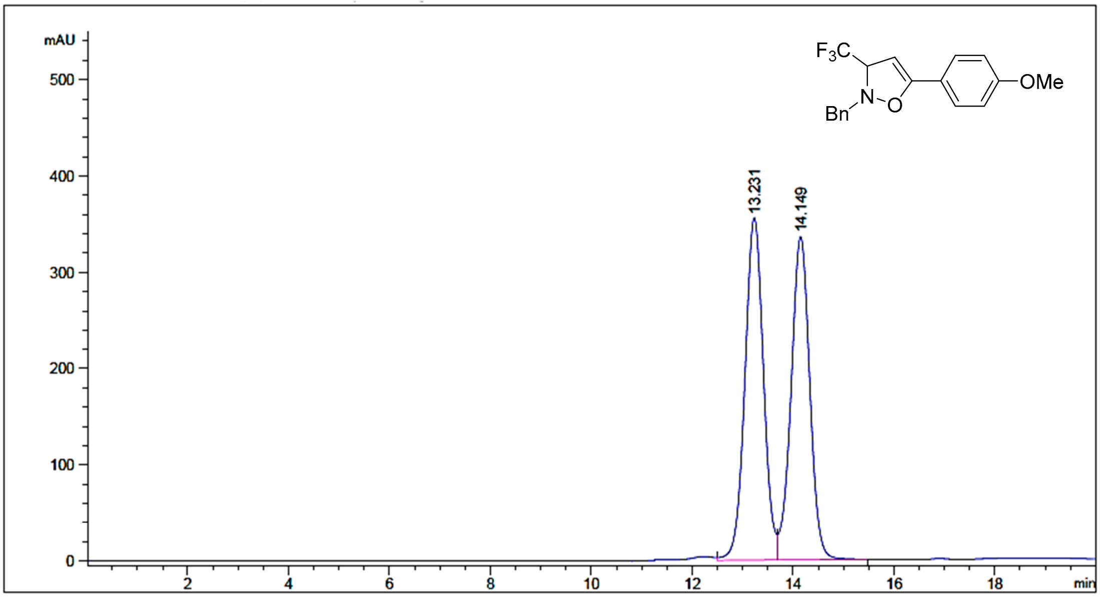

Signal 1: DAD1 A, Sig=250,4 Ref=off

Peak #	RetTime [min]	Type	Width [min]	Area [mAU*s]	Height [mAU]	Area %
1	13.231	VV	0.3964	9103.32617	355.97159	51.2411
2	14.149	VB	0.3967	8662.35645	336.04895	48.7589

2-Benzyl-5-(4′-chlorophenyl)-3-(trifluoromethyl)-2,3-dihydroizoxazole (**6ac**): *ee* determination condition: Chiralcel AD, Hexanes : ^{i}PrOH = 98:2, flow = 0.3 mL/min, retention time: 10.65, 11.24 min.

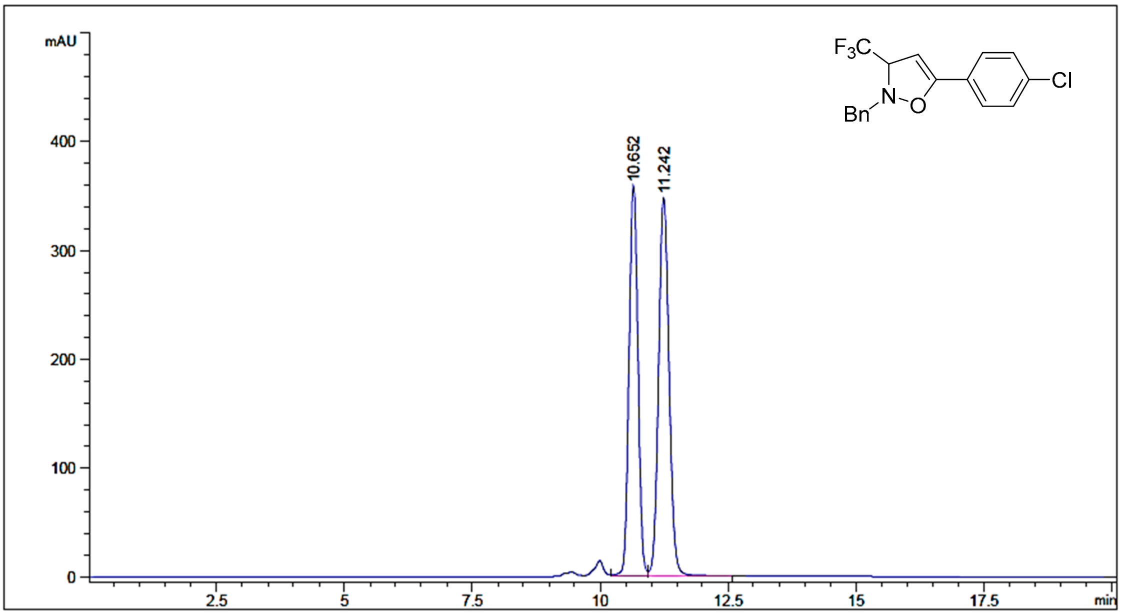

Signal 1: DAD1 A, Sig=250,4 Ref=off

Peak #	RetTime [min]	Type	Width [min]	Area [mAU*s]	Height [mAU]	Area %
1	10.652	VV	0.1951	4393.84912	358.26672	46.9812
2	11.242	VB	0.2224	4958.51270	347.61761	53.0188

2-Benzyl-5-(4′-trifluoromethylphenyl)-3-(trifluoromethyl)-2,3-dihydroizoxazole (**6ad**): *ee* determination condition: Chiralcel AD, Hexanes : ^{i}PrOH = 95:5, flow = 0.5 mL/min, retention time: 5.40, 5.78 min.

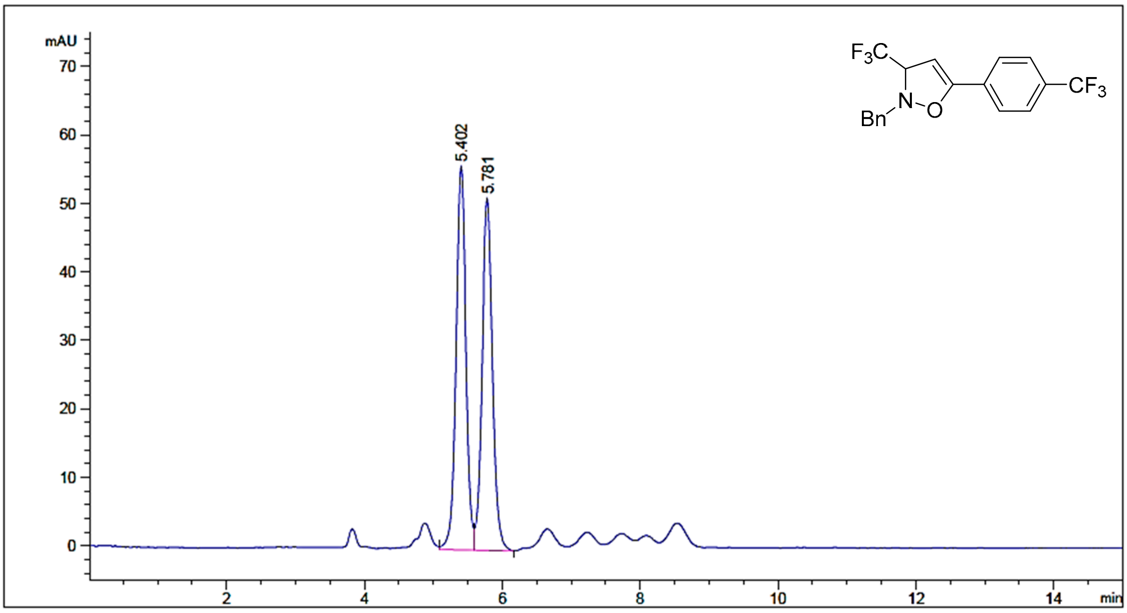

Signal 1: DAD1 A, Sig=250,4 Ref=off

Peak #	RetTime [min]	Type	Width [min]	Area [mAU*s]	Height [mAU]	Area %
1	5.402	VV	0.1537	555.96063	56.05902	50.8252
2	5.781	VB	0.1601	537.90692	51.40519	49.1748

XRD data collection and refinement

Single-crystal XRD measurements for **5aa** and **3a** were performed with a Rigaku XtaLAB Synergy, Pilatus 300K diffractometer. Measurements were conducted at 100.0(1) K using the CuK_{α} radiation (λ = 1.54184 Å). The data was integrated using CrysAlisPro program [43]. Intensities for absorption were corrected using gaussian method in SCALE3 ABSPACK scaling algorithm implemented in CrysAlisPro program.

CCDC: Files 2495338-2495339 contain the supplementary crystallographic data for this paper. These data can be obtained free of charge from The Cambridge Crystallographic Data Centre via www.ccdc.cam.ac.uk/structures

Structure solution and refinement

The structures were solved with the ShelXT [44] structure solution program using Intrinsic Phasing and refined in the ShelXle [45] by the full-matrix least-squares minimization on F^2 with the ShelXL [46] refinement package. All non-hydrogen atoms were refined anisotropically, and C–H hydrogens were generated geometrically using the HFIX command as in ShelXL. Hydrogen atoms were refined isotropically and constrained to ride on their parent atoms.

The crystal data and structure refinement descriptors are presented in Table S1. Molecular structures and partial packing diagrams for **5aa** and **3a** are shown in Figures S1 – S2.

Table S1. Selected structural data for 5aa and 3a

	**5aa** CCDC: 2495338	**3a** CCDC: 2495339
Formula	$C_{17}H_{14}F_3NO$	$C_9H_8NOF_3$
Formula Weight	305.29	203.16
Crystal System	triclinic	orthorhombic

Space Group	$P\bar{1}$	$P2_12_12_1$
a/Å	8.95570(10)	4.99803(5)
b/Å	9.34160(10)	13.17890(13)
c/Å	10.15670(10)	13.77931(15)
α/°	114.3600(10)	90
β/°	96.9920(10)	90
γ/°	100.8860(10)	90
Volume/Å^3	740.969(15)	907.623(16)
Z	2	4
2θ range for data collection/°	9.806 to 157.312	9.286 to 159.106
Index ranges	-11 ≤ h ≤ 10, -11 ≤ k ≤ 10, -12 ≤ l ≤ 12	-6 ≤ h ≤ 6, -16 ≤ k ≤ 16, -16 ≤ l ≤ 17
No. of measured, independent, and observed [$I>2\sigma(I)$] reflections	46531,3002, 2953	23731, 1923, 1914
R_{int}	0.0298	0.0296
Goodness-of-fit on F^2	1.079	1.070
Final R indexes [$F^2 > 2\sigma(F^2)$]	R_1 = 0.0327, wR_2 = 0.0814	R_1 = 0.0245, wR_2 = 0.0641
Final R indexes [all data]	R_1 = 0.0330, wR_2 = 0.0816	R_1 = 0.0246, wR_2 = 0.0641
Data/restraints/parameters	3002/0/200	1923/0/127
Largest diff. peak/hole Å^{-3}	0.32/-0.30	0.14/-0.17
Flack parameter	-	0.01(3)

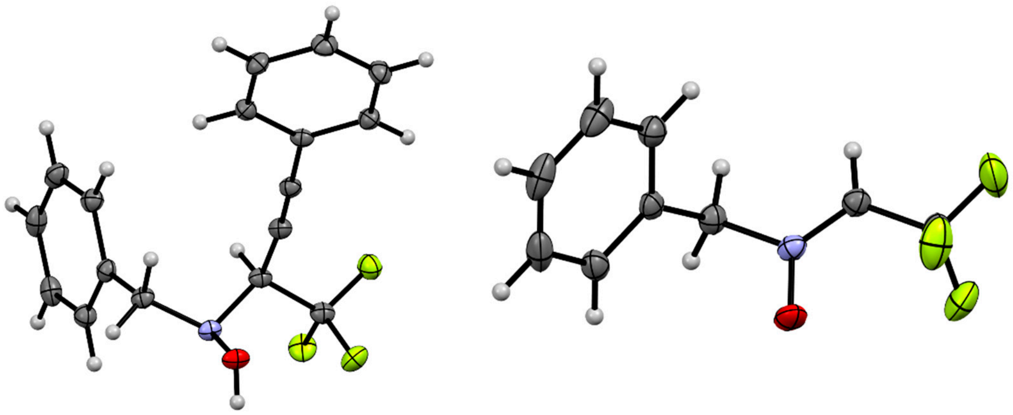

Figure S1. Molecular structure of **5aa** (left) and **3a** (right). Displacement ellipsoids are drawn at 50% probability level.

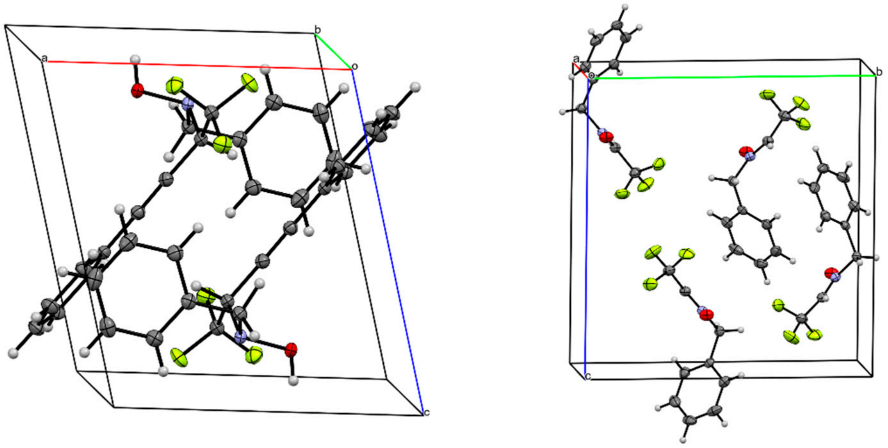

Figure S2. Partial packing diagram of **5aa** (left) and **3a** (right). Displacement ellipsoids are drawn at 50% probability level.

5aa crystallizes in triclinic $P\bar{1}$ space group while **3a** adopts orthorombic $P2_12_12_1$ setting. Both crystal structures contain one symmetry independent molecule defined by the nitrone group (**3a**) and analogous hydroxylamine group (**5aa**) linked with trifluoromethyl and benzyl groups (Figure S1). The molecular geometries of **5aa** and **3a** are defined by nearly planar C1-C2-N3-C8 fragment with torsion angles of -167° and -178°, respectively. The electron delocalization over the chain make oxygen O4 nearly coplanar with respect to the mean plane formed by the C1-C2-N3-C8 chain. In contrast, oxygen O4 in **5aa** is almost perpendicular to the chain forming C2-N3-O4 angle ~109°. Similarly, in both structures phenyl rings of benzyl group are nearly perpendicularly positioned to C1-C2-N3-C8 core with angles between mean planes of

the relevant fragments of 85° and 74° in **5aa** and **3a**, respectively. A phenylacetylene group present in **5aa** is twisted of about 54° referring to the C1-C2-N3-C8 mean plane. The molecular geometries in both structures are stabilized by intramolecular interactions between trifluoromethyl group and O4 oxygen atoms. The interactions are represented by short O…F contacts (vdW-0.19 Å in **5aa** and vdW-0.05 Å in **3a**).

The supramolecular motif defining the crystal packing in **3a** may be defined as continuous chains extending along [1 0 0] direction stabilized by non-classic hydrogen bonds C-H…O (Figure S3). The interaction is characterized by C…O distance of 3.160(2) Å and C-H…O angle 159.1°. Molecules in crystal structure of **5aa** form discrete centrosymmetric dimers associated through O-H…N hydrogen bonds between hydroxylamine groups of neighboring molecules. The hydrogen bond is characterized by O…N distance of 2.872(1) Å and O-H…N angle of 149.8°. The dimers are linked into chains by F…π interactions between trifloromethyl and phenylacetylene groups. The interactions are represented by short F…C contacts (vdW-0.01Å). Resulting chains extend along [0 0 1] direction (Figure S4).

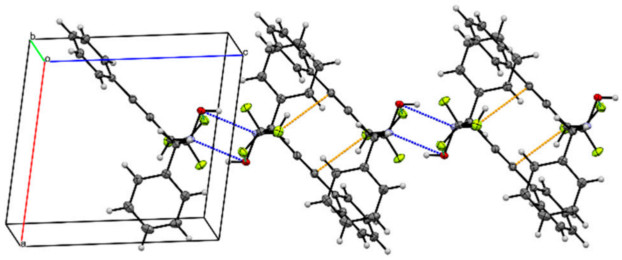

Figure S3. Selected interactions stabilizing molecular and supramolecular structure of **5aa**. Intramolecular interaction F…O - light-green dotted lines; intermolecular O-H…N hydrogen bonds -blue; F…π interactions – orange. Displacement ellipsoids are drawn at 50% probability level.

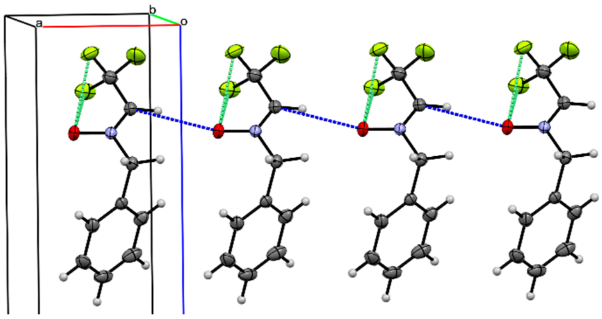

Figure S4. Selected interactions stabilizing molecular and supramolecular structure of **3a**. Intramolecular interaction F…O - light-green dotted lines; intermolecular O-H…N hydrogen bonds -blue. Displacement ellipsoids are drawn at 50% probability level.

Table S2 Bond Lengths for 3a_DEPO.

Atom	Atom	Length/Å	Atom	Atom	Length/Å
F5	C1	1.3326(19)	C8	C9	1.5069(19)
F6	C1	1.3416(18)	C9	C10	1.387(2)
F7	C1	1.3336(18)	C9	C14	1.393(2)
O4	N3	1.2791(14)	C10	C11	1.387(2)
N3	C2	1.2958(18)	C14	C13	1.393(2)
N3	C8	1.4952(17)	C11	C12	1.380(3)
C2	C1	1.4879(19)	C13	C12	1.389(3)

Table S3 Bond Angles for 5aa_DEPO.

Atom	Atom	Atom	Angle/°	Atom	Atom	Atom	Angle/°
O4	N3	C8	105.30(8)	C16	C15	C2	174.25(11)
O4	N3	C2	106.88(8)	C14	C9	C8	120.42(10)
C2	N3	C8	110.86(8)	C10	C9	C8	120.68(10)
C18	C17	C16	120.05(10)	C10	C9	C14	118.89(10)
C22	C17	C18	119.65(10)	C18	C19	C20	120.37(10)
C22	C17	C16	120.29(10)	C13	C14	C9	120.52(11)
N3	C8	C9	111.46(9)	N3	C2	C1	109.90(8)
F7	C1	F6	106.59(9)	C15	C2	N3	114.07(9)
F7	C1	C2	114.20(9)	C15	C2	C1	112.70(9)
F5	C1	F7	106.99(9)	C20	C21	C22	120.25(10)
F5	C1	F6	107.37(9)	C12	C11	C10	120.06(11)
F5	C1	C2	111.22(9)	C14	C13	C12	120.22(11)
F6	C1	C2	110.14(9)	C9	C10	C11	120.52(11)
C19	C18	C17	119.82(10)	C21	C20	C19	119.87(10)
C15	C16	C17	179.34(12)	C11	C12	C13	119.78(11)
C21	C22	C17	120.03(10)				

Table S4 Bond Angles for 3a_DEPO.

Atom	Atom	Atom	Angle/°	Atom	Atom	Atom	Angle/°
O4	N3	C2	124.48(13)	F7	C1	F5	107.43(12)
O4	N3	C8	115.74(12)	F7	C1	C2	113.37(12)
C2	N3	C8	119.78(11)	F6	C1	C2	112.98(12)
N3	C2	C1	120.33(12)	F5	C1	F6	106.62(13)
N3	C8	C9	110.23(11)	F5	C1	C2	109.47(12)
C10	C9	C8	119.24(12)	C9	C14	C13	119.62(15)
C10	C9	C14	120.00(13)	C12	C11	C10	119.93(15)
C14	C9	C8	120.76(13)	C12	C13	C14	119.85(15)
C9	C10	C11	120.18(15)	C11	C12	C13	120.41(15)
F7	C1	F6	106.61(13)				

Table S5 Torsion Angles for 5aa_DEPO.

A	B	C	D	Angle/°	A	B	C	D	Angle/°
F7	C1	C2	N3	-57.35(12)	C8	C9	C14	C13	179.24(10)
F7	C1	C2	C15	71.08(12)	C8	C9	C10	C11	-178.03(10)
F5	C1	C2	N3	-178.56(8)	C18	C17	C22	C21	0.92(16)
F5	C1	C2	C15	-50.13(12)	C18	C19	C20	C21	0.30(18)
F6	C1	C2	N3	62.53(11)	C16	C17	C18	C19	178.38(10)
F6	C1	C2	C15	-169.04(9)	C16	C17	C22	C21	-178.36(10)
O4	N3	C8	C9	-175.28(8)	C22	C17	C18	C19	-0.89(16)
O4	N3	C2	C1	78.66(10)	C22	C21	C20	C19	-0.27(18)
O4	N3	C2	C15	-49.02(11)	C9	C14	C13	C12	-1.24(17)
N3	C8	C9	C14	63.11(13)	C14	C9	C10	C11	0.75(16)
N3	C8	C9	C10	-118.12(11)	C14	C13	C12	C11	0.81(17)
C17	C18	C19	C20	0.29(17)	C2	N3	C8	C9	69.48(11)
C17	C22	C21	C20	-0.34(17)	C10	C9	C14	C13	0.45(16)
C8	N3	C2	C1	-167.09(8)	C10	C11	C12	C13	0.39(17)
C8	N3	C2	C15	65.24(11)	C12	C11	C10	C9	-1.18(17)

Table S6 Torsion Angles for 3a_DEPO.

A	B	C	D	Angle/˚	A	B	C	D	Angle/˚
O4	N3	C2	C1	2.0(2)	C8	C9	C10	C11	-179.77(13)
O4	N3	C8	C9	64.80(15)	C8	C9	C14	C13	179.67(13)
N3	C2	C1	F7	59.46(18)	C9	C10	C11	C12	0.1(2)
N3	C2	C1	F6	-61.97(18)	C9	C14	C13	C12	0.1(2)
N3	C2	C1	F5	179.39(13)	C10	C9	C14	C13	-0.1(2)
N3	C8	C9	C10	83.72(15)	C10	C11	C12	C13	0.0(2)
N3	C8	C9	C14	-96.08(15)	C14	C9	C10	C11	0.0(2)
C2	N3	C8	C9	-114.97(14)	C14	C13	C12	C11	-0.1(2)
C8	N3	C2	C1	-178.24(12)					

[43] Agilent *CrysAlis PRO*. Agilent Technologies Ltd, Yarnton, Oxfordshire, England, 2014.

[44] Sheldrick, G. M. *Acta Cryst.* **2015**, A**71**, 3–8. https://doi.org/10.1107/S2053273314026370.

[45] Hübschle, C. B.; Sheldrick, G. M.; Dittrich, B. *J. Appl. Cryst.* **2011**, *44*, 1281–1284. http://dx.doi.org/10.1107/S0021889811043202.

[46] Sheldrick, G. M. *Acta Cryst.* **2015**, C**71**, 3–8. http://dx.doi.org/10.1107/S2053229614024218.
